# GPTBioInsightor—leveraging large language models for transparent scRAN-seq cell type annotations

**DOI:** 10.1093/bioadv/vbag025

**Published:** 2026-01-22

**Authors:** Shenghui Huang, Berina Šabanović, Yuzhong Peng, Quan Zheng, Luca Alessandri, Christopher Heeschen

**Affiliations:** Department of Molecular Biotechnology and Health Sciences, University of Turin, Turin (Torino) 10126, Italy; Pancreatic Cancer Heterogeneity, Candiolo Cancer Institute - FPO - IRCCS, Candiolo (Torino) 10060, Italy; Molecular Biotechnology Center, University of Turin, Turin (Torino) 10126, Italy; Pancreatic Cancer Heterogeneity, Candiolo Cancer Institute - FPO - IRCCS, Candiolo (Torino) 10060, Italy; Faculty of Health Sciences, University of Macau, Macau SAR 999078, China; Center for Single-Cell Omics, School of Public Health, Shanghai Jiao Tong University School of Medicine, Shanghai 200025, China; State Key Laboratory of Systems Medicine for Cancer, Shanghai Jiao Tong University School of Medicine, Shanghai 200025, China; Department of Molecular Biotechnology and Health Sciences, University of Turin, Turin (Torino) 10126, Italy; Molecular Biotechnology Center, University of Turin, Turin (Torino) 10126, Italy; Pancreatic Cancer Heterogeneity, Candiolo Cancer Institute - FPO - IRCCS, Candiolo (Torino) 10060, Italy

## Abstract

**Motivation:**

Large language models (LLMs) are rapidly becoming indispensable across the life‑sciences spectrum, from literature mining through clinical decision support to experimental design. Yet, in single‑cell RNA‑sequencing (scRNA‑seq) analysis, most LLM‑enabled tools remain opaque: they output a single label per cluster without disclosing the chain‑of‑ thought that led to that decision. This opaqueness undermines reproducibility, complicates peer‑review, and ultimately slows the adoption of otherwise powerful methods.

**Results:**

We developed GPTBioInsightor, an LLM‑powered assistant that not only annotates cell types, cell states, and pathway activities but also narrates how it arrived at each conclusion, step-by-step. Across benchmark datasets—including peripheral blood mononuclear cells (PBMC3K) and pancreatic ductal adenocarcinoma—GPTBioInsightor achieved at least parity with expert manual curation while delivering transparent reasoning, confidence scores, and literature‑based evidence. By closing the “interpretability gap,” GPTBioInsightor equips wet‑lab biologists, computational scientists, and reviewers with an audit‑ready trail, thereby accelerating discovery and fostering trust in AI‑assisted bioinformatics.

**Availability and implementation:**

GPTBioInsightor is freely available on GitHub under a BSD-3-Clause license (https://github.com/huang-sh/GPTBioInsightor).

## 1 Introduction

The last decade has witnessed an explosion of single-cell technologies capable of profiling tens to hundreds of thousands of individual cells in a single experiment. These datasets capture subtle transcriptional shifts that underpin development, immune activation, and tumor evolution. However, the downstream interpretation—specifically, the assignment of a biological identity to each transcriptionally defined cluster—remains a bottleneck. Traditionally, biologists manually scour the literature for marker genes and laboriously match them to cluster-specific differential-expression (DE) lists such as CellMarker (Hu *et al.* 2023) and PanglaoDB (Franzén *et al.* 2019). This manual approach is: Time-consuming: Annotating a complex tumor microenvironment with dozens of cell states can take weeks; Knowledge-dependent: Even senior domain experts may be unfamiliar with the newest markers or rare sub-populations; Reproducibility-limited: Annotation criteria are often implicit and undocumented, hampering peer review and meta-analysis.

Automated annotators such as, e.g. singleR (Aran *et al.* 2019), SCSA (Cao *et al*. 2020), and ScType (Ianevski *et al.* 2022) alleviate some of these issues, but they still behave largely as black boxes. Recent large language model (LLM)-centric tools, e.g. GPTCelltype (Hou and Ji 2024), push the envelope by achieving near-expert accuracy. Beyond cell type annotation, LLMs exhibits outstanding capabilities across diverse biomedical applications, such as gene set summarization and discovery (Joachimiak *et al.* 2024, Hu *et al.* 2025), interpretation of scientific figures (Wang *et al.* 2024), helping to democratize medical knowledge (Zhu *et al.* 2023), and advancing biomedical discovery (Gao *et al.* 2024). Yet they too provide minimal disclosure of intermediate reasoning. Consequently, users often face a dilemma: trust the machine blindly or spend additional time validating its output. Motivated by this gap, we designed GPTBioInsightor with three guiding principles:

1. Transparency by default—every prediction is accompanied by a plain-language explanation, ranked evidence, and confidence metrics;
2. Context awareness—annotations incorporate user-specified tissue, disease stage, and experimental conditions;
3. Comprehensive insight—beyond cell-type labels, the system infers functional states and enriched biological pathways, providing a holistic cellular portrait.

## 2 Methods

Software implementation. GPTBioInsightor is written entirely in Python. Large-language-model requests are handled through the community SDKs openAI and anthropic, which provide thin wrappers around the respective REST APIs. The PerplexityAI SDK was integrated to support paper searching. Differential-expression results are converted to functional signatures with GSEApy (Fang *et al*. 2023). The resulting pathway lists are passed, together with marker genes and biological context, to the Insightor agent for integrative reasoning. Each cluster is annotated separately, after which the predicted cell type labels are integrated and standardized to achieve uniform naming across clusters. To optimize program performance, we have implemented a multi-threading running way for concurrent execution. We use “claude-3–5-sonnet-20241022” model in cast study analysis.

Prompt engineering. The system-level prompt was adapted from the open-source prompt (https://github.com/richards199999/Thinking-Claude) and tailored to the biological domain. The name_pathway and analyse_pathway prompts were imported verbatim from (Hu *et al.* 2025). Cell-type annotation, scoring, and reporting—all other prompt chains were developed in-house to support the GPTBioInsightor workflow.

Performance Evaluation. We adopted the same evaluation strategy as GPTCelltype (Hou and Ji 2024) and conducted performance comparisons on their established benchmark data. Specifically, annotation similarity was quantified using a three-level matching scheme: Full match (1.0) for identical terminology and Cell Ontology (CL) terms; Partial match (0.5) for shared hierarchical relationships or aligned broad categories; and Mismatch (0.0) for annotations with no shared CL ancestry or divergent classifications. And GPTBioInsightor will output 3 cell-type candidates, hence we will use the max agreement score for benchmark evaluation. The evaluation was performed on three models: GPT-4o, Qwen-Max, and DeepSeek-Chat.

## 3 Results

### 3.1 System overview

GPTBioInsightor is an AI assistant that leverages LLMs to extract biological insights from scRNA-seq data. As summarized in Table S1, available as supplementary data at *Bioinformatics Advances* online, the tool aids researchers in two key stages of analysis: (i) cell-type and cell-state annotation and (ii) gene-set functional interpretation. GPTBioInsightor can call APIs from multiple LLM providers (Table S2, available as supplementary data at *Bioinformatics Advances* online), including widely used models such as ChatGPT, Claude, DeepSeek, and Qwen. The primary input is an AnnData object (Virshup *et al.* 2024), the core data structure of the scverse community (Virshup *et al.* 2023). scverse offers a comprehensive computational ecosystem for single-cell omics, hosting popular packages such as scanpy (Wolf, Angerer and Theis 2018), scvi-tools (Gayoso *et al.* 2022), OmicVerse (Zeng *et al.* 2024), and tangram (Biancalani *et al.* 2021). GPTBioInsightor itself is part of this ecosystem (https://scverse.org/packages/#ecosystem), benefiting from continuous testing, thorough documentation, and a user-friendly API.

### 3.2 Cell type analysis

**Figure 1:**
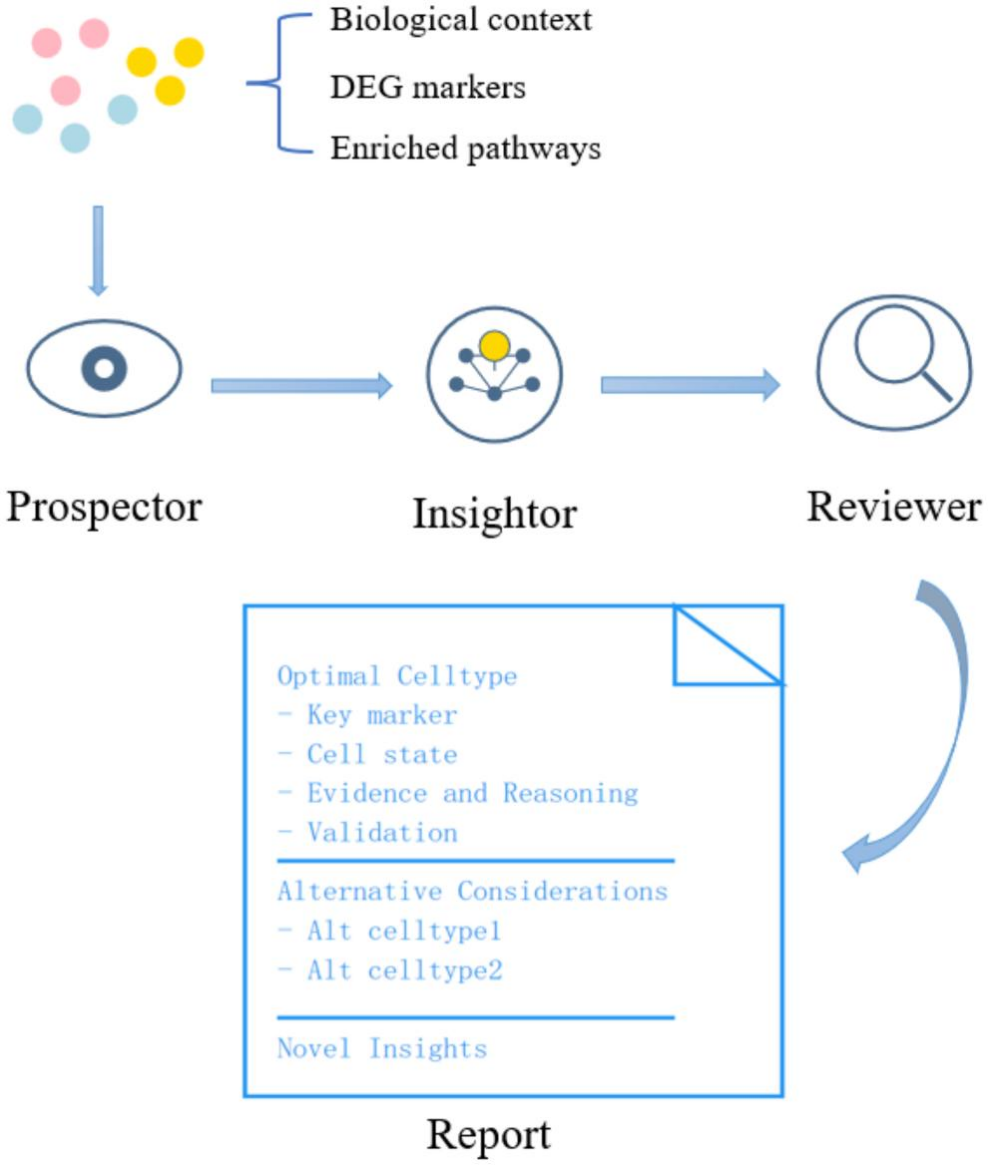
Three-stage workflow. **Prospector**—Drafts a shortlist of plausible cell identities based on tissue context and canonical markers; **Insightor**—Synthesizes DE genes, pathway enrichments, and contextual priors to produce a ranked annotation for each cluster; **Reviewer**—Performs cross-model consensus checking, assigns confidence scores, and flags low-agreement clusters for manual follow-up.

For the cell-type module (Fig. 1), we adopt a step-back prompting strategy (Zheng *et al.* 2024). Rather than asking an LLM to label each cluster outright, we first invoke a Prospector agent that, using user-supplied biological context, assembles a shortlist of plausible cell types. This targeted candidate list keeps subsequent predictions biologically meaningful. Next, the Insightor agent integrates three evidence streams—differentially expressed genes (DEGs), the same biological context, and pathway-enrichment results—to infer the most likely cell type for every cluster. Finally, a Reviewer agent evaluates and ranks each prediction.

For each cluster, GPTBioInsightor receives (i) biological context, (ii) top DEG markers, and (iii) enriched pathways. Context (tissue source, disease state, experimental condition, etc.) steers the LLM away from biologically implausible answers. Relying on DEG lists alone is risky, because factors such as the chosen DEG algorithm or residual rRNA contamination can distort the top-gene set. By contrast, pathway enrichment summarizes dozens to hundreds of genes and thus supplies a more stable, function-level signal (Heumos *et al.* 2023).

### 3.3 Cell type annotation performance

**Figure 2:**
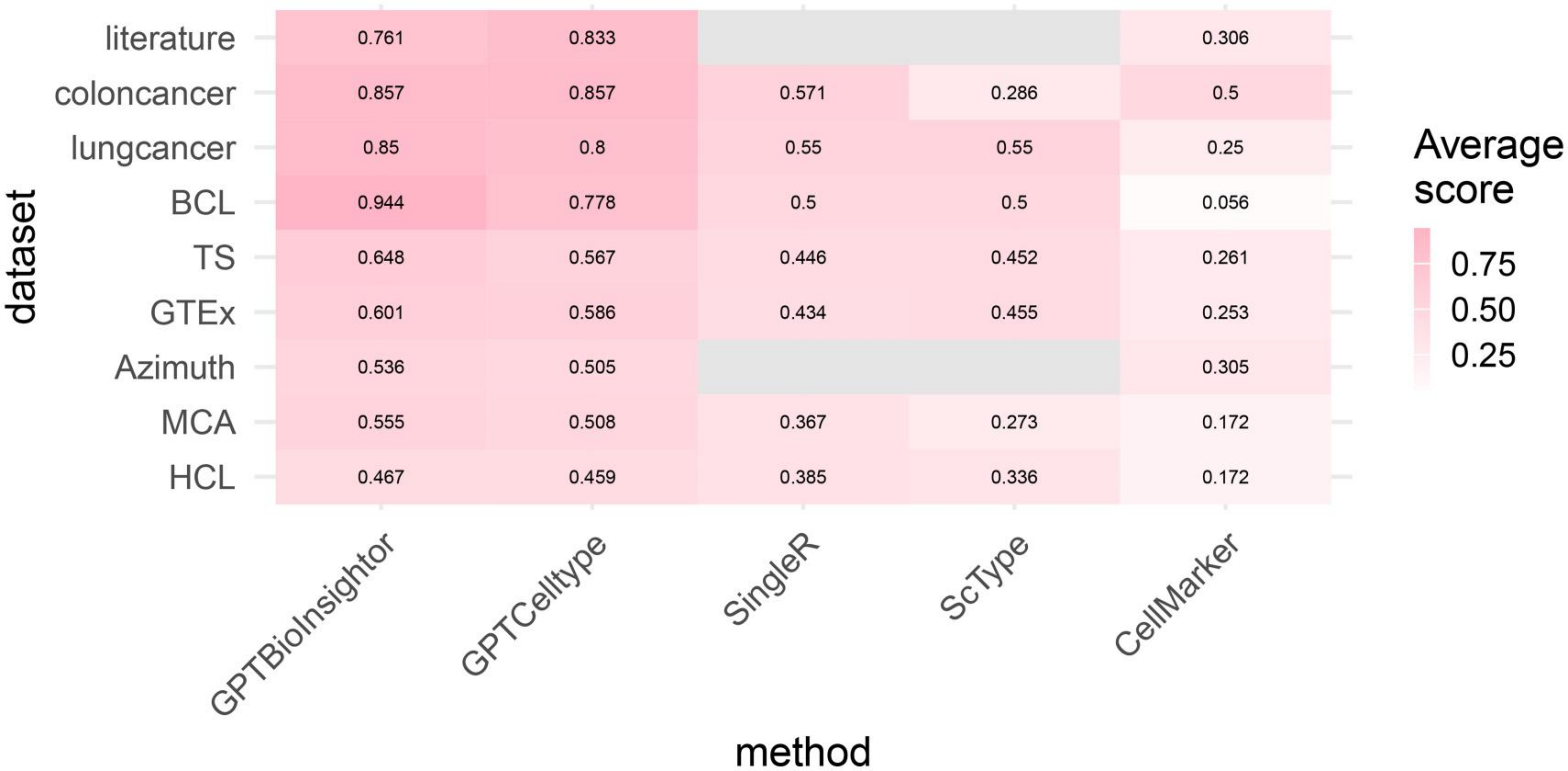
Average agreement scores. Comparison for GPTBioinsightor (gpt-4o), GPTCelltype (gpt4), singleR, scType and CellMarker.

To systematically assess cell type annotation performance, we compared the performance of GPTBioinsightor using the established GPTCelltype benchmark dataset. The benchmark encompasses five species and includes hundreds of tissues and cell types, covering both normal and cancer samples. It also provides the annotation performance of multiple reference tools, including CellMarker, SingleR, and ScType. Overall, GPTBioinsightor, powered by GPT-4o, consistently outperforms other methods based on the average agreement scores (Fig. 2 and Fig. S1, available as supplementary data at *Bioinformatics Advances* online).

We further evaluated the scalability and computational efficiency of GPTBioInsightor (Table S3, available as supplementary data at *Bioinformatics Advances* online). The model maintained stable performance on datasets containing over 50 000 cells, demonstrating its capacity to handle large-scale single-cell data. Notably, the computational complexity of GPTBioInsightor does not scale with the total number of cells, but rather with the number of clusters, as increases in cluster count lead to proportional growth in runtime and computational cost (Table S3). To assess model generalizability, we additionally evaluated the performance of Qwen-Max and DeepSeek-Chat (Fig. S1, available as supplementary data at *Bioinformatics Advances* online). The results indicate that GPTBioInsightor performs robustly across different LLMs.

**Case study 1**—PBMC3K dataset. To gauge utility of the GPTBioInsightor, we first analyzed the well-studied PBMC3K dataset, a small, well-curated reference dataset containing ∼2700 PBMCs from a single healthy donor. GPTBioInsightor’s annotations matched manual labels with no substantive discrepancies (Table S4, available as supplementary data at *Bioinformatics Advances* online). Crucially, the tool also produced a detailed report (Info 1, available as supplementary data at *Bioinformatics Advances* online) that goes beyond simple labels: for each cluster it lists candidate cell types, presents DEG evidence, provides reasoning, assigns confidence scores, and supplies additional gold-standard markers for validation. Specifically, the report provides three cell type candidates for each cell cluster. And for each candidate, a cell type name, cell type score, cell state, key markers, supportive evidence and reasoning and other current gold standard biomarkers for validation.

**Case study 2**—Pancreatic cancer dataset. We next tested the more complex CRA001160 pancreatic cancer dataset derived from 24 primary pancreatic-ductal-adenocarcinoma (PDAC) tumors and 11 histologically normal pancreases from surgical resections and includes 57 530 high-quality transcriptomes after quality control (Peng *et al.* 2019). GPTBioInsightor again produced accurate labels (Table S5, available as supplementary data at *Bioinformatics Advances* online) while adding biologically nuanced insights (Info 2, available as supplementary data at *Bioinformatics Advances* online). Notably, the authors’ labeled cluster 8 as “stellate cells” based on markers such as RGS5, ACTA2, PDGFRB, and ADIRF. Because quiescent stellate cells do not normally express fibrosis-associated ACTA2, GPTBioInsightor instead flagged the cluster as activated stellate cells or cancer-associated fibroblasts—an interpretation consistent with its strong myofibroblast signature. Likewise, cluster 3 was originally called “ductal 2” but later shown (via CNV analysis) to be cancerous; GPTBioInsightor identified it as cancer cells from the outset, offering an early clue that could guide downstream hypotheses. Such context-rich outputs are especially valuable for interdisciplinary researchers or newcomers to single-cell analysis, providing clearer functional interpretations and accelerating biological discovery.

### 3.4 Cell-type scoring system

To steer the LLM’s reasoning and give researchers transparent quality metrics, we designed two complementary frameworks for cell-type evaluation: Three-component score (Table 1) combines (i) marker-gene expression, (ii) pathway-enrichment signals, and (iii) biological context. Two-component score (Table S6, available as supplementary data at *Bioinformatics Advances* online) is used when pathway data are unavailable and therefore integrates only (i) marker genes and (ii) context. Both schemes award positive points for concordant evidence and apply deductions for conflicting signals, yielding a balanced confidence score for every cluster–cell-type pair. Beyond the default scoring implementation, users can define their own custom scoring schemes. A built-in heat-map function (Table S1 and  Figs S2 and S3, available as supplementary data at *Bioinformatics Advances* online) visualizes these scores, allowing users to compare overall confidence across clusters and to spot similarities between putative cell types.

**Table 1:** Cell‑type scoring rubric (pathway‑available version).

Evidence stream	Scoring criterion	Rationale	Points^a^
Marker profile	Matching cell‑type/state markers detected	Strong evidence the cluster represents the proposed identity	+45 (max)
	Narrow (high‑specificity) markers detected	Supports a fine‑grained subtype or activation state	+15 (max)
	Shares markers with a different cell type/state	Indicates ambiguity; lowers confidence	−10
	Negative markers (should not be expressed) present	Contradicts the assignment	−30
Pathway profile	Enriched pathways fit the cell state	Captures functional programs (e.g. “interferon response”)	+15 (max)
	Enriched pathways fit the cell type	Broad biological match (e.g. “T‑cell receptor signaling”)	+5 (max)
	Pathway also enriched in another candidate	Lowers specificity	−10
	Conflicting pathways	Functional mismatch	−20
Biological context	Cell type plausible in tissue/condition	Aligns with prior knowledge	+10 (max)
	Cell state plausible in tissue/condition	Supports activation/differentiation status	+10 (max)
	Cell type/state biologically implausible	Contradicts experimental context	−30

a Indicates the maximum positive contribution or fixed deduction applied when the criterion is met.

### 3.5 Gene-set functional analysis

Hu et al. showed that LLMs excel at deciphering the collective biological functions of gene sets, and they introduced Gene Set AI, a web server offering “Process Naming” and “Biological Process Analysis” modules (Hu *et al.* 2025). To give researchers greater flexibility, e.g. tapping into a broader roster of LLMs and processing multiple gene sets simultaneously, we have embedded Gene Set AI’s prompting strategy directly within GPTBioInsightor.

## 4 Discussion and conclusion

GPTBioInsightor transforms LLMs into transparent reasoning partners for single-cell analysis through its three-agent “step-back prompting” pipeline—Prospector → Insightor → Reviewer. By integrating DE signatures, pathway enrichment, and user-defined biological context, the system provides structured reasoning, ranked cell-type predictions, and quantitative confidence scores that bridge automation with interpretability. Applied to two representative datasets (PBMC3K and the pancreatic-cancer atlas CRA001160) GPTBioInsightor not only reproduced expert annotations but also surfaced deeper insights. Benchmarking with the GPTCelltype dataset, which spans five species and hundreds of tissues across normal and cancer samples, showed that GPTBioInsightor consistently outperformed CellMarker, SingleR, and ScType in average agreement scores (Fig. 2). The model exhibited strong scalability, maintaining stable accuracy on datasets exceeding 50 000 cells, with runtime scaling primarily with cluster number rather than total cell count. Additional evaluations with Qwen-Max and DeepSeek-Chat confirmed the robustness and cross-model generalizability of the framework.

Within Gao et al.’s AI-agent taxonomy (Gao *et al.* 2024), GPTBioInsightor aligns with class 1–2, serving both as an intelligent assistant and as an interpretable collaborator in single-cell discovery. Seamlessly integrated into the scverse ecosystem, it provides a modular, extensible, and reproducible Python framework that supports diverse LLM endpoints. By embedding transparency, quantitative rigor, and rich reporting within a familiar analysis workflow, GPTBioInsightor lowers the barrier for interdisciplinary teams while maintaining analytical depth for experts. Looking ahead, its adaptable architecture will enable integration of multi-omic and spatial transcriptomic data, further extending its analytical scope. Ultimately, GPTBioInsightor exemplifies how agentic LLM systems can already act as dependable partners in biological discovery, converting black-box predictions into explainable, data-driven insights that advance both computational and experimental single-cell research.

## Supplementary Material

vbag025_Supplementary_Data

## Data Availability

The PBMC3K single-cell RNA-sequencing dataset was obtained from 10x Genomics and is publicly available at https://support.10xgenomics.com/single-cell-gene-expression/datasets/1.1.0/pbmc3k. The pancreatic ductal adenocarcinoma (PDAC) scRNA-seq dataset analyzed in this study is available from the Genome Sequence Archive (GSA) under accession number CRA001160. Benchmark datasets used for performance evaluation were obtained from the GPTCelltype repository (Hou and Ji, 2024) and are publicly available at https://github.com/Winnie09/GPTCelltype_Paper/.

## Code availability

The complete source code and prompts can be accessed at https://github.com/huang-sh/GPTBioInsightor. GPTBioInsightor is developed in Python and is conveniently available on the Python Package Index (PyPI), the official repository for third-party Python software. Installation is straightforward and can be accomplished using pip commands (visit: https://pypi.org/project/gptbioinsightor/ for more details). For extensive online documentation and guidance, please refer to https://gptbioinsightor.readthedocs.io/en/latest/index.html.
