# Supplementary material for: GPTBioInsightor—leveraging large language models for transparent scRAN-seq cell type annotations
Source: Bioinform Adv. 2026 Jan 22;6(1):vbag025. doi: 10.1093/bioadv/vbag025 (PMC12975716; doi:10.1093/bioadv/vbag025)
Supplement: vbag025_Supplementary_Data [file vbag025_supplementary_data.zip › 31-Jan-2026_055833_Supplementary_info_2.docx]

**CellType Analysis**

**Potential CellType**

In scRNA-Seq data background of 'PDAC (Pancreatic Ductal Adenocarcinoma) patients tumor', the following Potential CellType to be identified:

| Plain Text Let me analyze all the cell types and states mentioned across previous responses... Need to integrate them while ensuring biological relevance and detectability in scRNA-seq.  Starting with malignant cells - cancer cells are definitely key, marked by KRT19, EPCAM. Their states typically show proliferation markers like MKI67 or EMT markers like VIM, SNAI1.  For immune cells... T cells are marked by CD3 genes, showing states from activated (IL2, IFNG) to exhausted (PDCD1, LAG3). B cells express CD19, MS4A1, with states ranging from memory (CD27) to plasma cells (SDC1, JCHAIN).  Myeloid compartment - macrophages express CD68, showing M1 (IL1B, TNF) vs M2 (CD163, MRC1) polarization. DCs with CD1C marker showing mature (CCR7) or immature (CD1A) states.  Stromal cells... Fibroblasts express FAP, showing myofibroblastic (ACTA2) or inflammatory (IL6) phenotypes. Endothelial cells with PECAM1, VWF markers can be angiogenic (VEGFR2) or quiescent.  Need to consider marker specificity and reliability in scRNA-seq context... Some markers might be more robust than others. Also thinking about state-specific markers that are well-validated in PDAC context.  Looking at overlap and differences between previous responses... Some cell types like mast cells and neutrophils were mentioned but might be less reliably detected. Should focus on most consistently identifiable populations.  Let me organize this into clear categories with well-established markers... |
| --- |

Candidate celltype:

Cancer Cells: KRT19, EPCAM

- Proliferative state: MKI67, TOP2A, high metabolic activity
- EMT/Invasive state: VIM, SNAI1, ZEB1, enhanced migration

T Cells: CD3D, CD3E, CD3G

- Activated state: IL2, IFNG, TNF, enhanced cytokine production
- Exhausted state: PDCD1, LAG3, TIGIT, reduced effector function

Macrophages: CD68, CD14

- M1-like state: IL1B, TNF, NOS2, pro-inflammatory
- M2-like state: CD163, MRC1, IL10, immunosuppressive

Fibroblasts: FAP, COL1A1

- Myofibroblastic state: ACTA2, TAGLN, contractile phenotype
- Inflammatory state: IL6, CXCL12, secretory phenotype

B Cells: CD19, MS4A1

- Memory state: CD27, TNFRSF13B, antigen-experienced
- Plasma cell state: SDC1, JCHAIN, antibody-secreting

Endothelial Cells: PECAM1, VWF

- Quiescent state: CLDN5, TIE1, stable vessels
- Angiogenic state: KDR, VEGFR2, vessel formation

Dendritic Cells: CD1C, CLEC10A

- Mature state: CCR7, CD83, antigen-presenting
- Immature state: CD1A, FLT3, surveillance

NK Cells: NCAM1, FCGR3A

- Cytotoxic state: GNLY, PRF1, killer function
- Exhausted state: LAG3, TIGIT, reduced cytotoxicity

Ductal Cells: KRT7, KRT19

- Normal state: CFTR, CA2, secretory function
- Stress state: SOX9, DCLK1, altered differentiation

Mast Cells: KIT, TPSAB1

- Resting state: TPSB2, CPA3, granule maintenance
- Activated state: VEGFA, IL6, degranulation

**cluster geneset 0**

**Gene List**

Top genes

| Plain Text CPA2,PLA2G1B,PRSS1,CTRB2,CTRC,PRSS3,CTRB1,PNLIPRP1,CPB1,KLK1,CEL,CELA3A,CELA3B,REG1A,CPA1,SPINK1,CLPS,GP2,SYCN,PNLIP,CELA2A,GATM,CELA2B,CTRL,AMY2A,MT1G,PDIA2,AMY2B,ALB,SERPINI2,RNASE1,ERP27,CUZD1,LGALS2,AQP8,ANPEP,MT1X,NUPR1,P4HB,SSR4,TMEM97,CTC-479C5.12,PABPC4,FGL1,GSTA2,MT1H,XBP1,RARRES2,REG1B,GAMT |
| --- |

enrichment pathway

| Plain Text  - WikiPathways_2024_Human: Zinc Homeostasis ,Copper Homeostasis ,Trans Sulfuration One Carbon Metabolism And Related Pathways ,Methionine Metabol To Sulfur Amino As And Related Disorders ,Abnormal Ca Handling And Effects On Muscle Contraction In DMD |
| --- |

**celltype thinking**

| Plain Text Hmm, looking at this gene signature from PDAC tumor... Very interesting pattern here. Let me dive deep into what these genes are telling us about cellular identity and state.  First striking observation - there's a strong enrichment of pancreatic enzymes: PRSS1, PRSS3, CPA1, CPA2, CTRB1, CTRB2... This immediately makes me think of acinar cells, the protein-producing workhorses of the pancreas. The presence of amylases (AMY2A, AMY2B) further supports this.  But wait, need to consider the disease context - this is PDAC. Could these be remnant normal acinar cells? Or could this represent acinar-to-ductal metaplasia (ADM), a key process in PDAC development? The presence of NUPR1 is particularly intriguing here - it's a stress response factor involved in ADM.  Looking at the secretory program - lots of protein processing and secretion related genes like P4HB, SSR4, XBP1. This fits with professional secretory cells. The presence of REG1A/B is interesting - these are involved in regeneration and metaplasia.  The metallothioneins (MT1G, MT1X, MT1H) suggest cellular stress response. In pancreatic context, this often indicates oxidative stress during transformation or inflammation.  The pathway enrichment is telling - zinc/copper homeostasis, sulfur metabolism... These are critical for protein folding and cellular stress management. The calcium handling pathway makes sense given the secretory nature.  Let me think about the candidate cell types from our list... While some markers overlap with ductal cells (like protein secretion), this profile is much more acinar-like. The stress/metaplasia signature suggests cells potentially undergoing transformation.  Could this also be a specialized subset of cancer cells that have retained or reactivated acinar programs? Need to consider plasticity in PDAC...  For validation, I should look for classic acinar markers not in this set - PTF1A would be gold standard. For ADM/plasticity, SOX9 or PDX1 status would be informative.  The more I analyze this, the clearer pattern emerges - this looks like acinar-derived cells in various states of stress/transformation... |
| --- |

Based on comprehensive analysis of the gene expression pattern, pathway enrichment, and disease context, here are the three most likely cell types/states:

1. Pancreatic Acinar Cells

- Primary markers: PRSS1, CPA1/2, CTRB1/2, AMY2A/B
- Functional signature: Professional protein secretion (P4HB, SSR4, XBP1)
- State: Stressed/reactive (MT1G/X/H, NUPR1)
- Validation markers: PTF1A, RBPJL, MIST1

1. Acinar-to-Ductal Metaplasia (ADM) Cells

- Key markers: REG1A/B, NUPR1
- Stress response program: Metallothioneins, protein folding machinery
- Metabolic adaptation: Altered sulfur metabolism
- Validation markers: SOX9, PDX1

1. Acinar-like Cancer Cells

- Retained acinar enzymes: PRSS1/3, CPAs
- Stress/transformation markers: NUPR1, REG proteins
- Altered metabolism: Zinc/copper homeostasis
- Validation markers: KRAS mutation status, TP53

The gene signature strongly suggests cells of acinar origin, with evidence of stress response and potential plasticity characteristic of PDAC pathogenesis. The presence of both differentiated acinar programs and stress/metaplasia markers indicates these cells may represent different stages of acinar cell transformation in the tumor microenvironment.

**Score**

| Plain Text Let me carefully evaluate each predicted cell type using the scoring criteria with pathways since we have pathway information...  Starting with Pancreatic Acinar Cells: Marker profile analysis - extremely strong match here. Multiple classical acinar enzymes like PRSS1, CPA1/2, AMY2A/B - these are highly specific acinar markers, not just pancreatic. The secretory program (P4HB, SSR4) supports but is shared with other secretory cells. No negative markers present. Marker score: ~40 for matching markers + ~12 for narrow specificity - 10 for shared = 42  Pathway match - The protein processing/folding pathways perfectly align with acinar cell biology. Calcium handling makes sense for secretory function. Zinc/copper homeostasis critical for enzyme processing. Pathway score: ~15 for cell state + ~5 for cell type = 20  Context - Acinar cells are absolutely expected in PDAC, especially showing stress response. Context score: 20  Total for Acinar: ~82  For ADM Cells: Markers - REG1A/B and NUPR1 are good ADM markers, but less specific than acinar markers. The stress response program fits but is shared. Marker score: ~35 for matching + ~8 for narrow - 10 for shared = 33  Pathways - The stress and metabolic pathways align well with ADM state. Pathway score: ~12 for state + ~3 for type = 15  Context - ADM is a key feature in PDAC development. Context score: 20  Total for ADM: ~68  For Acinar-like Cancer Cells: Markers - Retains some acinar markers but less specific for cancer state. Stress markers present but shared. Marker score: ~30 for matching + ~5 for narrow - 10 for shared = 25  Pathways - Metabolic changes fit cancer state but less specifically. Pathway score: ~10 for state + ~2 for type = 12  Context - Definitely plausible in PDAC. Context score: 20  Total for Cancer: ~57 |
| --- |

Pancreatic Acinar Cells: 82

Acinar-to-Ductal Metaplasia (ADM) Cells: 68

Acinar-like Cancer Cells: 57

**Report**

**Celltype Prediction**

**Pancreatic Acinar Cells (score: 82)**

Cell state: Stressed/reactive secretory state

**Key Markers**:

- Cell-type-specific: PRSS1, CPA1/2, CTRB1/2, AMY2A/B, PNLIP, CEL, CELA2A/B, CELA3A/B
- Cell-state-specific: MT1G/X/H, NUPR1, P4HB, SSR4, XBP1

**Evidence and Reasoning**

The gene signature shows definitive acinar cell identity through multiple highly specific pancreatic enzyme markers (PRSS1, CPA1/2, CTRB1/2) that represent the core acinar secretory program. This is reinforced by the presence of additional digestive enzymes (AMY2A/B, PNLIP) and protein processing machinery (P4HB, SSR4, XBP1). The enriched pathways strongly support active protein synthesis and secretion, with zinc/copper homeostasis pathways critical for enzyme processing and calcium handling essential for secretory function. The presence of stress response genes (metallothioneins, NUPR1) and altered metabolic pathways indicates these acinar cells are in a reactive state, likely responding to the tumor microenvironment. This comprehensive profile matches the expected biological behavior of acinar cells under stress conditions in PDAC.

**Validation**: PTF1A, RBPJL, MIST1, NR5A2

**Acinar-to-Ductal Metaplasia Cells (score: 68)**

Cell state: Transitional metaplastic state

**Key Markers**:

- Cell-type-specific: REG1A/B, NUPR1
- Cell-state-specific: MT1G/X/H, P4HB, SSR4, PDIA2

**Evidence and Reasoning**

The expression profile suggests cells undergoing acinar-to-ductal metaplasia, a critical process in PDAC development. The presence of REG1A/B and NUPR1, known markers of pancreatic metaplasia and stress response, indicates active cellular reprogramming. The retention of some secretory pathway components (P4HB, SSR4) while showing stress response (metallothioneins) and altered metabolism (sulfur amino acid pathways) is characteristic of cells in transition. The enriched pathways related to protein processing and cellular stress response support this transitional state. This molecular signature aligns with the biological process of ADM, where acinar cells undergo profound changes in cellular identity and function in response to inflammatory and oncogenic signals in the pancreatic tumor microenvironment.

**Validation**: SOX9, PDX1, HNF1B

**Acinar-like Cancer Cells (score: 57)**

Cell state: Transformed state with retained acinar features

**Key Markers**:

- Cell-type-specific: PRSS1/3, CPAs
- Cell-state-specific: NUPR1, REG1A/B, MT1G/X/H

**Evidence and Reasoning**

The molecular profile indicates cancer cells that have maintained aspects of acinar differentiation while showing characteristics of transformation. The presence of select pancreatic enzymes (PRSS1/3, CPAs) suggests retained acinar programs, while stress response elements (NUPR1, metallothioneins) and regeneration markers (REG1A/B) indicate cellular transformation. The enriched metabolic pathways, particularly alterations in zinc/copper homeostasis and sulfur metabolism, reflect the metabolic rewiring common in cancer cells. This profile is consistent with the phenomenon of incomplete differentiation often observed in PDAC, where cancer cells maintain certain tissue-specific features while acquiring malignant characteristics.

**Validation**: KRAS mutation status, TP53, SMAD4

**Novel Insights**

The analysis reveals a complex interplay between cellular identity and stress response in pancreatic cancer. The presence of strong acinar markers alongside stress response elements suggests that maintaining certain aspects of acinar function might be important in PDAC biology. The enrichment of metal homeostasis pathways, particularly zinc and copper metabolism, points to a previously underappreciated role of metal ion regulation in pancreatic cell state transitions. The co-expression of secretory programs and stress response elements indicates that these cells may be utilizing their protein processing machinery for purposes beyond traditional enzyme secretion, possibly contributing to tumor microenvironment modification.

**cluster geneset 1**

**Gene List**

Top genes

| Plain Text MS4A1,CD79A,CD37,CD52,PTPRCAP,LTB,CD74,HLA-DRA,CD79B,LAPTM5,HLA-DPB1,CXCR4,CORO1A,VPREB3,HLA-DPA1,HLA-DQB1,HLA-DQA1,EEF1B2,ARHGDIB,CD53,EEF1A1,PABPC1,FAU,EEF2,HLA-DRB1,LIMD2,RAC2,GNB2L1,IRF8,UBA52,BANK1,NCF1,SMIM14,UCP2,CD48,OAZ1,RCSD1,GPSM3,CD19,HLA-DMB,LSP1,FCRLA,LY86,CYBA,PTPRC,HLA-DMA,RHOH,SLC25A6,NPM1,BTG1 |
| --- |

enrichment pathway

| Plain Text  - WikiPathways_2024_Human: Cytoplasmic Ribosomal Proteins ,DNA Replication ,Retinoblastoma Gene In Cancer ,Extrafollicular And Follicular B Cell Activation By SARS CoV 2 ,B Cell Receptor Signaling |
| --- |

**celltype thinking**

| Plain Text Hmm, looking at this gene signature from PDAC tumor context... Let me dive deep into the molecular patterns here.  First thing that jumps out at me is the strong B cell signature - MS4A1 (CD20), CD79A/B, CD19... These are absolutely classic B cell markers. And not just any B cells - the presence of VPREB3 suggests some immature/transitional B cells might be present. The enrichment of BCR signaling pathway really reinforces this.  The HLA genes are fascinating - HLA-DRA, DPB1, DPA1, DQB1, DQA1... Such a strong antigen presentation signature. Initially makes me think of professional APCs like dendritic cells, but wait - B cells are also professional APCs. This fits perfectly with the B cell hypothesis.  PTPRC (CD45) and other immune markers like CD37, CD52 clearly indicate we're looking at immune cells. The presence of BANK1 is particularly interesting - it's specifically involved in B cell receptor signaling.  Looking at the pathway analysis... B Cell Receptor Signaling jumps out immediately. The presence of ribosomal and DNA replication pathways suggests these cells are actively proliferating and producing antibodies, which makes sense in a tumor context where B cells might be responding to tumor antigens.  Let me think about alternative cell types... While some markers like CD74 and HLA genes are shared with dendritic cells and macrophages, the complete absence of classic markers like CD14, CD68 (macrophages) or CD1C (DCs) makes these less likely. The strong co-expression of MS4A1, CD79A/B, and CD19 is really specific for B cells.  The extrafollicular and follicular B cell activation pathway enrichment is particularly telling - suggests these might be tumor-infiltrating B cells, possibly forming tertiary lymphoid structures which are known to occur in PDAC.  Looking back at the candidate cell types... The marker profile matches best with B cells, particularly showing features of both memory and activated states based on the high expression of antigen presentation machinery.  Let me consider potential states... The high expression of ribosomal proteins and DNA replication pathways suggests these are actively proliferating and differentiating B cells, likely responding to tumor antigens.  For validation, I would look for additional markers not in this set like CD27 (memory B cells), CD38 (plasma cell differentiation), or IGHD/IGHM (naive B cells) to further refine the B cell state. |
| --- |

Based on comprehensive analysis of the molecular profile, pathway enrichment, and biological context, here are the three most likely cell type possibilities ranked by confidence:

1. B Cells (Highest Confidence)

- Primary Evidence: Strong expression of definitive B cell markers (MS4A1/CD20, CD79A/B, CD19)
- Supporting Evidence:
- BCR signaling pathway enrichment
- B cell-specific transcription factor IRF8
- B cell-specific signaling molecule BANK1
- Validation Markers: CD27, CD38, IGHD/IGHM

1. Memory B Cells (Moderate Confidence)

- Primary Evidence: High expression of antigen presentation machinery (HLA class II genes)
- Supporting Evidence:
- Active protein synthesis machinery (ribosomal pathways)
- Presence of CXCR4 suggesting tissue homing
- Validation Markers: CD27, CCR6, TNFRSF13B

1. Activated B Cells (Moderate Confidence)

- Primary Evidence: DNA replication pathway activation
- Supporting Evidence:
- High expression of protein synthesis machinery
- B cell activation pathway enrichment
- Validation Markers: CD86, CD69, AICDA

The molecular profile most strongly supports these cells being B lymphocytes, with features suggesting they are likely activated and possibly differentiating in response to the tumor microenvironment.

**Score**

| Plain Text Let me carefully evaluate each predicted cell type using the scoring criteria with pathway since pathway data is provided...  Starting with B Cells: Marker Profile analysis... Strong matching markers: CD19, MS4A1(CD20), CD79A/B are highly specific B cell markers (+45) Narrow markers: BANK1, VPREB3, FCRLA are very B cell specific (+15) Common markers like PTPRC, CD74, HLA genes are shared with other immune cells (-10) No negative markers present  Pathway analysis... BCR signaling perfectly matches B cell type (+5) Extrafollicular/follicular B cell activation matches state (+15) Ribosomal/DNA replication pathways are shared with other proliferating cells (-10) No conflicting pathways  Context analysis... B cells are well-documented in PDAC tumor microenvironment (+10) Activated state makes sense in tumor context (+10) Total: 80 points  For Memory B Cells: Marker Profile... HLA class II expression matches memory state but less specific (+30) No highly specific memory markers like CD27 in dataset (+5) Shares markers with other B cell states (-10)  Pathway... Protein synthesis pathway matches activated state (+15) Pathways shared with other activated cells (-10)  Context... Memory B cells plausible in PDAC (+10) Memory state reasonable (+10) Total: 50 points  For Activated B Cells: Marker Profile... HLA and protein synthesis machinery matches activated state (+35) No highly specific activation markers like CD86 (+5) Shares markers with memory state (-10)  Pathway... DNA replication/protein synthesis matches activated state (+15) B cell activation pathway strongly supports (+15) Some pathway overlap with other activated cells (-10)  Context... Activated B cells common in tumors (+10) Activation state fits context (+10) Total: 70 points  Let me double-check my scoring... Yes, this reflects the relative confidence levels appropriately based on the marker and pathway evidence. |
| --- |

B Cells: 80

Activated B Cells: 70

Memory B Cells: 50

**Report**

**Celltype Prediction**

**Celltype1: B Cells (score: 80)**

Actively proliferating and differentiating state with enhanced antigen presentation capacity

**Key Markers**:

- Cell-type-specific: MS4A1(CD20), CD79A/B, CD19, BANK1, VPREB3, FCRLA
- Cell-state-specific: HLA-DRA, HLA-DPB1, HLA-DPA1, HLA-DQB1, EEF1A1, EEF2

**Evidence and Reasoning**

The molecular profile strongly indicates B lymphocytes based on multiple lines of evidence. The expression of canonical B cell markers (MS4A1, CD79A/B, CD19) combined with B cell-specific signaling molecules (BANK1) and developmental markers (VPREB3) provides definitive identification. The enrichment of B Cell Receptor Signaling pathway and Extrafollicular/Follicular B Cell Activation pathways strongly supports this identification. The high expression of HLA class II genes and protein synthesis machinery (EEF1A1, EEF2) suggests these B cells are actively engaged in antigen presentation and antibody production. This profile is consistent with tumor-infiltrating B cells in PDAC, where they often form tertiary lymphoid structures and participate in anti-tumor immune responses.

**Validation**: CD27, CD38, IGHD/IGHM, CD20, PAX5

**Celltype2: Activated B Cells (score: 70)**

Highly proliferative state with enhanced protein synthesis

**Key Markers**:

- Cell-type-specific: MS4A1, CD79A/B, CD19
- Cell-state-specific: HLA class II genes, EEF1A1, EEF2, FAU, NPM1

**Evidence and Reasoning**

The strong expression of protein synthesis machinery (EEF1A1, EEF2, FAU) and DNA replication pathway enrichment indicates an activated state. The presence of B cell activation pathways and high expression of antigen presentation machinery (HLA class II genes) suggests these cells are actively responding to antigenic stimulation. The molecular profile shows characteristics of B cells undergoing proliferation and differentiation, supported by the expression of NPM1 involved in ribosome biogenesis. This activated state is particularly relevant in the PDAC tumor microenvironment, where B cells encounter tumor antigens and mount immune responses. The enrichment of extrafollicular and follicular B cell activation pathways further supports this activated state.

**Validation**: CD86, CD69, AICDA, CD25, Ki-67

**Celltype3: Memory B Cells (score: 50)**

Antigen-experienced state with enhanced antigen presentation capacity

**Key Markers**:

- Cell-type-specific: MS4A1, CD79A/B, CD19
- Cell-state-specific: HLA class II genes, CXCR4

**Evidence and Reasoning**

The high expression of antigen presentation machinery (HLA class II genes) combined with the chemokine receptor CXCR4 suggests these cells might be memory B cells with tissue-homing capacity. The presence of protein synthesis and B cell receptor signaling pathways indicates maintained ability to respond to antigens. While the profile shares features with activated B cells, the expression pattern is consistent with antigen-experienced memory B cells, which are known to maintain higher levels of antigen presentation machinery compared to naive B cells. This phenotype is relevant in the PDAC context, where memory B cells can persist in the tumor microenvironment and contribute to ongoing anti-tumor immune responses.

**Validation**: CD27, CCR6, TNFRSF13B, CD80, ITGAL

**Novel Insights**

The molecular profile reveals a potentially important role for B cells in PDAC tumor microenvironment, showing features of both activation and memory formation. The strong expression of antigen presentation machinery suggests these B cells might serve as efficient antigen-presenting cells in the tumor context, beyond their traditional antibody-producing role. The presence of VPREB3, typically associated with B cell development, in combination with activation markers, might indicate local B cell differentiation within the tumor microenvironment. The enrichment of extrafollicular and follicular B cell activation pathways suggests these cells might be involved in forming tertiary lymphoid structures within the tumor, which could have implications for patient prognosis and treatment response.

**cluster geneset 2**

**Gene List**

Top genes

| Plain Text FXYD2,ANXA4,SLC4A4,CLDN10,CLU,CFTR,DEFB1,ATP1A1,PEBP1,AMBP,RBP1,SH3YL1,SERPINA5,MT1F,SCTR,GMNN,SORBS2,SLC3A1,ATP1B1,C12orf75,GATM,LEFTY1,SFRP5,CITED4,UGT2A3,SOD3,NGFRAP1,KRT18,EPCAM,HMGN3,COX7C,HOMER2,KRT8,CCND1,BEX1,GSTP1,KIF12,AKAP7,PPP1R1B,FAM150B,CLDN3,CA2,PPDPF,KCNJ16,PPAP2C,CD59,DCDC2,RP11-528G1.2,FGGY,S100A13 |
| --- |

enrichment pathway

| Plain Text  - WikiPathways_2024_Human: Ciliopathies ,Bardet Biedl Syndrome ,Genes Related To Primary Cilium Development Based On CRISPR ,Leucine Isoleucine And Valine Metabolism ,Fatty Acid Beta Oxidation |
| --- |

**celltype thinking**

| Plain Text Hmm, looking at this PDAC tumor context and the geneset... Let me dive deep into these markers and pathways to understand what cell population we're dealing with.  First striking observation - EPCAM and KRT8/18 immediately catch my attention. These are classic epithelial markers. In pancreatic context, this is particularly interesting. The presence of CFTR and CA2 is making me think about ductal identity specifically.  Going through the gene list more carefully... CLDN3, CLDN10 - tight junction components typical of epithelial cells, especially ductal cells. SLC4A4 and ATP1A1/B1 are involved in ion transport, which is a key function of ductal cells.  The pathway enrichment is fascinating - ciliopathies and primary cilium development. Primary cilia are characteristic features of ductal cells, acting as cellular antennae for signaling. This fits beautifully with a ductal identity.  But what's the state of these cells? Seeing CCND1 suggests proliferative capacity, but also noticing CLU which can be stress-related. GSTP1 often indicates cellular stress response too. These cells might be under some form of stress or adaptation.  Need to consider the PDAC context carefully... These could be: 1. Normal ductal cells 2. Malignant ductal cells 3. Precursor lesion cells  The presence of RBP1 and CITED4 is interesting - these can be involved in differentiation programs. SFRP5 as a WNT pathway modulator could indicate active developmental signaling.  Looking at metabolic signatures... FGGY and fatty acid metabolism pathways suggest altered metabolism, which could be consistent with malignant transformation.  Let me cross-reference with the candidate cell types... The marker profile aligns well with ductal cells, but shows features of both normal and transformed states. The epithelial signature is strong, but there are hints of stress/adaptation.  Need to consider what gold standard markers could validate these populations... Looking for KRT7, KRT19, SOX9 would be helpful, though they're not in our current set.  The more I look at this, the clearer it becomes - we're likely looking at ductal-derived cells, but need to carefully distinguish their exact state... |
| --- |

Based on comprehensive analysis of the markers, pathways, and biological context, here are the three most likely cell types/states:

1. Pancreatic Ductal Cells (Normal/Differentiated)

- Key markers: CFTR, CA2, CLDN3/10, SLC4A4
- Supporting evidence:
- Expression of ductal function genes (ion transport, tight junctions)
- Enrichment of cilium-related pathways
- Epithelial markers (EPCAM, KRT8/18)
- Gold standard validation markers: KRT7, KRT19, MUC1

1. Early-Stage Transformed Ductal Cells

- Key markers: CCND1, GSTP1, CLU
- Supporting evidence:
- Proliferation markers
- Stress response genes
- Altered metabolic signatures
- Gold standard validation markers: TP63, SOX9, EGFR

1. Ductal Progenitor-like Cells

- Key markers: RBP1, SFRP5, CITED4
- Supporting evidence:
- Developmental signaling components
- Plasticity markers
- Mixed differentiation states
- Gold standard validation markers: PDX1, HNF1B, SOX9

These populations likely represent a spectrum of ductal cell states within the PDAC tumor microenvironment, ranging from normal-like to early transformation states.

**Score**

| Plain Text Let me carefully evaluate each predicted cell type using the scoring criteria, considering the PDAC context and available pathways...  Starting with Pancreatic Ductal Cells (Normal/Differentiated): Marker Profile analysis... Strong matching markers: CFTR, CA2, CLDN3/10 (classical ductal markers) EPCAM, KRT8/18 (epithelial markers, though shared with other epithelial cells) Ion transport markers (SLC4A4, ATP1A1/B1) strongly support ductal function No negative markers present Calculating... ~40 for matching markers, ~12 for narrow markers, -10 for shared markers  Pathway relevance... Ciliopathies and primary cilium development directly relate to ductal cell biology Metabolic pathways are consistent with normal ductal function About 15 points for cell state pathways, 5 for cell type  Context plausibility... Completely plausible in PDAC, both type and state Full 20 points for context  For Early-Stage Transformed Ductal Cells: Markers: CCND1, GSTP1, CLU indicate transformation But these are more state markers than definitive transformed markers Some overlap with normal ductal markers ~35 for matching, ~8 for narrow, -10 for shared  Pathways: Metabolic changes fit transformation state But not highly specific ~10 for state pathways  Context: Highly plausible in PDAC Full context points  For Ductal Progenitor-like Cells: Markers: RBP1, SFRP5, CITED4 These are more general developmental markers Less specific for ductal progenitors ~30 for matching, ~5 for narrow, -10 for shared  Pathways: Some relevance to developmental state But not highly specific ~8 for state pathways  Context: Plausible but less common in PDAC ~15 for context  Let me finalize the scores... |
| --- |

Pancreatic Ductal Cells (Normal/Differentiated): 82

Early-Stage Transformed Ductal Cells: 73

Ductal Progenitor-like Cells: 58

**Report**

**Celltype Prediction**

**Pancreatic Ductal Cells (score: 82)**

Normal/differentiated state with active secretory function

**Key Markers**:

- Cell-type-specific: CFTR, CA2, CLDN3, CLDN10, SLC4A4, ATP1A1, ATP1B1
- Cell-state-specific: EPCAM, KRT8, KRT18, PPDPF

**Evidence and Reasoning**

- The expression profile strongly indicates differentiated ductal epithelial identity through multiple ion transport and secretory markers (CFTR, CA2, SLC4A4). The presence of tight junction components (CLDN3, CLDN10) and epithelial markers (EPCAM, KRT8/18) further supports ductal cell identity. The enrichment of ciliopathy-related pathways is particularly significant as primary cilia are essential features of ductal cells, serving as cellular sensors and signaling hubs. The metabolic pathway enrichment, including amino acid metabolism, aligns with the high metabolic demands of secretory ductal cells. This cell population maintains classical ductal functions while showing signs of active epithelial maintenance and secretory activity, consistent with normal ductal cells within the PDAC microenvironment.

**Validation**: KRT7, KRT19, MUC1, HNF1B

**Early-Stage Transformed Ductal Cells (score: 73)**

Stress-adaptive state with proliferative features

**Key Markers**:

- Cell-type-specific: EPCAM, KRT8, KRT18
- Cell-state-specific: CCND1, GSTP1, CLU, PEBP1

**Evidence and Reasoning**

- This population exhibits characteristics of ductal cells undergoing early transformation, evidenced by the co-expression of epithelial markers and stress-response genes. The presence of CCND1 indicates active cell cycle progression, while stress-response proteins (GSTP1, CLU) suggest cellular adaptation. The enrichment of metabolic pathways, particularly fatty acid metabolism, reflects metabolic reprogramming common in early transformation. The expression of PEBP1, a metastasis suppressor, along with maintenance of epithelial markers suggests these cells are in an early stage of transformation rather than fully transformed. This profile is consistent with ductal cells adapting to the PDAC microenvironment, potentially representing an intermediate state between normal and fully malignant phenotypes.

**Validation**: TP63, SOX9, EGFR, SMAD4

**Ductal Progenitor-like Cells (score: 58)**

Plastic state with developmental features

**Key Markers**:

- Cell-type-specific: EPCAM, KRT8, KRT18
- Cell-state-specific: RBP1, SFRP5, CITED4

**Evidence and Reasoning**

- This population shows characteristics of cells with progenitor-like features, marked by the expression of developmental regulators. The presence of SFRP5 suggests active modulation of WNT signaling, a key pathway in ductal cell plasticity. RBP1 and CITED4 expression indicates involvement in differentiation programs. The enrichment of developmental pathways and primary cilium-related genes supports a state of cellular plasticity. While maintaining basic epithelial features (EPCAM, KRT8/18), these cells express markers suggesting potential for phenotypic adaptation. This profile is consistent with a subset of ductal cells that may have acquired progenitor-like features in response to the PDAC microenvironment.

**Validation**: PDX1, SOX9, HNF1B, NOTCH1

**Novel Insights**

The analysis reveals a complex spectrum of ductal cell states within the PDAC microenvironment. The presence of ciliopathy-related pathways alongside classical ductal markers suggests a previously underappreciated role for primary cilia in maintaining ductal cell identity and function in PDAC. The co-expression of stress response genes with epithelial markers indicates adaptive mechanisms that might represent early stages of malignant transformation. The identification of cells with progenitor-like features suggests cellular plasticity may be an important feature of ductal cell populations in PDAC, potentially contributing to disease progression through phenotypic adaptation.

**cluster geneset 3**

**Gene List**

Top genes

| Plain Text C19orf33,KRT19,S100A6,FXYD3,MUC1,SMIM22,SLPI,TSPAN1,CLDN4,AGR2,LGALS3,TMC5,S100P,SPINT2,MAL2,S100A11,GPRC5A,ELF3,CTSE,KRT8,OCIAD2,LGALS4,LCN2,LSR,SFN,KLF5,PDZK1IP1,CSTB,NQO1,KRT7,HN1,CAMK2N1,S100A14,EPCAM,C15orf48,SERINC2,KRT18,TSPAN8,PHLDA2,GPX2,LAMB3,MSLN,LIPH,WFDC2,RAB11FIP1,SLC44A4,LYZ,CEACAM6,TFF1,SDCBP2 |
| --- |

enrichment pathway

| Plain Text  - WikiPathways_2024_Human: Pancreatic Cancer Subtypes ,Enterocyte Cholesterol Metabolism ,Cholesterol Biosynthesis Pathway ,Cholesterol Synthesis Disorders ,Aerobic Glycolysis Augmented |
| --- |

**celltype thinking**

| Plain Text Hmm, looking at this PDAC tumor context and the gene signature... Let me dive deep into this molecular profile.  First striking observation - strong epithelial signature with multiple keratins KRT19, KRT8, KRT18, KRT7. This immediately points to epithelial origin. And EPCAM, a classic epithelial marker is there too. This is characteristic of pancreatic ductal cells.  The presence of MUC1, AGR2, CLDN4... these are typical of pancreatic ductal adenocarcinoma cells. MUC1 is particularly interesting as it's often overexpressed in PDAC and associated with poor prognosis. CLDN4 involvement in tight junctions makes perfect sense for ductal cells.  Looking at the S100 family members - S100P, S100A6, S100A11, S100A14... This is fascinating. These calcium-binding proteins are heavily implicated in PDAC progression. S100P particularly is considered a key player in pancreatic cancer.  The metabolic pathway enrichment is telling... Cholesterol metabolism pathways, aerobic glycolysis - classic cancer cell metabolic reprogramming. This fits with the Warburg effect we typically see in cancer cells.  Wait, there's something else interesting here... MSLN (mesothelin) expression. While it's a PDAC marker, it's also expressed by mesothelial cells. But given the overall epithelial signature and PDAC context, this is more likely representing malignant cells.  The presence of CEACAM6, TFF1... these are typically expressed in gastrointestinal epithelial cells, but in PDAC they're often upregulated in cancer cells. CEACAM6 particularly is associated with pancreatic adenocarcinoma progression.  Looking at transcription factors - ELF3, KLF5... These are epithelial-specific transcription factors. ELF3 is particularly interesting as it's known to regulate epithelial cell differentiation and is often dysregulated in PDAC.  The presence of GPRC5A, a retinoic acid-inducible protein, along with markers like SPINT2 and LAMB3, strongly suggests these are epithelial-derived cancer cells rather than normal ductal cells.  Given the strong epithelial signature but with markers of malignant transformation, this most likely represents:  1. PDAC Cancer Cells (highest probability) 2. Ductal Cells (possible but less likely given malignancy markers) 3. Metastatic PDAC Cells (possible given invasive markers)  Let me double-check against some gold standard markers not in this set... Yes, we'd expect to see TP63, SOX2, or GATA6 for normal ductal cells. Their absence combined with these malignancy markers further supports these being cancer cells.  The pathway analysis showing altered metabolism and PDAC subtypes really seals it - these are most likely malignant ductal adenocarcinoma cells in different states of differentiation or progression. |
| --- |

Based on comprehensive analysis of markers, pathways, and biological context, here are the three most likely cell types in order of probability:

1. PDAC Cancer Cells

- Key markers present: KRT19, EPCAM, MUC1, CLDN4, S100P, CEACAM6
- Supporting pathways: Aerobic glycolysis, cholesterol metabolism (cancer-associated metabolism)
- Biological context: Perfect match for PDAC tumor environment
- Gold standard validation markers (not in set): Would expect GATA6, TP63

1. Malignant Ductal Cells

- Key markers present: KRT7, KRT19, AGR2, CLDN4
- Supporting pathways: PDAC subtypes pathway
- Biological context: Derived from pancreatic ducts
- Gold standard validation markers (not in set): Would expect SOX9, HNF1B

1. Metastatic PDAC Cells

- Key markers present: MSLN, LAMB3, S100 family proteins
- Supporting pathways: Cholesterol metabolism (linked to metastasis)
- Biological context: Advanced PDAC often presents with metastasis
- Gold standard validation markers (not in set): Would expect SNAI1, TWIST1

**Score**

| Plain Text Let me carefully evaluate each predicted cell type using the scoring criteria with pathway since pathways were provided...  Starting with PDAC Cancer Cells: Marker Profile analysis... Strong epithelial markers (KRT19, EPCAM) are classical PDAC markers, that's solid points. MUC1, CLDN4, S100P, CEACAM6 are narrow markers highly specific for PDAC. No negative markers present. These are pretty much textbook PDAC markers, should get nearly full points here.  Pathway Profile... Aerobic glycolysis is absolutely characteristic of cancer cells - Warburg effect. Cholesterol metabolism pathways align perfectly with cancer cell metabolic reprogramming. PDAC subtypes pathway is directly relevant. No conflicting pathways. Strong score here.  Context is perfect - these are exactly what you'd expect in PDAC tumor.  For Malignant Ductal Cells: Marker Profile... KRT7/19 and CLDN4 are shared with normal ductal cells, so some point deduction there. AGR2 is more cancer-specific. No negative markers, but less specific overall.  Pathways partially match - ductal cells can show some metabolic changes but not typically this extreme. Some point deduction needed.  Context is reasonable but less specific than pure cancer cells.  For Metastatic PDAC Cells: Marker Profile... MSLN and LAMB3 are relevant but not exclusive to metastatic cells. S100 proteins are shared with primary PDAC. No negative markers but less specific overall.  Pathways are compatible but not distinctively metastatic.  Context is possible but requires additional assumptions about metastatic state.  Let me calculate final scores...  PDAC Cancer Cells: Markers: 43 (matching) + 15 (narrow) = 58 Pathways: 15 (state) + 5 (type) = 20 Context: 10 (type) + 10 (state) = 20 Total: 98  Malignant Ductal Cells: Markers: 35 (matching) + 10 (narrow) - 10 (shared) = 35 Pathways: 10 (state) + 5 (type) - 10 (shared) = 5 Context: 10 (type) + 10 (state) = 20 Total: 60  Metastatic PDAC Cells: Markers: 30 (matching) + 5 (narrow) - 10 (shared) = 25 Pathways: 10 (state) + 5 (type) - 10 (shared) = 5 Context: 10 (type) + 5 (state) = 15 Total: 45 |
| --- |

PDAC Cancer Cells: 98

Malignant Ductal Cells: 60

Metastatic PDAC Cells: 45

**Report**

**Celltype Prediction**

**PDAC Cancer Cells (score: 98)**

Highly proliferative and metabolically active malignant state

**Key Markers**:

- Cell-type-specific: KRT19, EPCAM, MUC1, CLDN4, AGR2
- Cell-state-specific: S100P, CEACAM6, MSLN, LAMB3, S100A11, S100A14

**Evidence and Reasoning**

The strong epithelial signature is marked by multiple keratins (KRT19, KRT8, KRT18, KRT7) and EPCAM, definitively establishing the epithelial origin. The presence of MUC1, AGR2, and CLDN4 is particularly characteristic of PDAC cells. The S100 family proteins (S100P, S100A11, S100A14) are heavily implicated in PDAC progression, with S100P being a key driver. The metabolic reprogramming evident in the enriched pathways - aerobic glycolysis and cholesterol metabolism - strongly supports the malignant phenotype, reflecting the Warburg effect typical in cancer cells. The expression of CEACAM6 and MSLN further confirms the PDAC identity, as these markers are frequently upregulated during pancreatic adenocarcinoma progression.

**Validation**: GATA6, TP63, SOX9

**Malignant Ductal Cells (score: 60)**

Transformed ductal state with altered differentiation

**Key Markers**:

- Cell-type-specific: KRT7, KRT19, CLDN4
- Cell-state-specific: AGR2, SPINT2, GPRC5A

**Evidence and Reasoning**

The expression profile shows strong ductal epithelial characteristics through KRT7 and KRT19, fundamental markers of ductal identity. The presence of CLDN4 indicates maintained tight junction components typical of ductal architecture. However, the expression of AGR2 and SPINT2 suggests a transformed state rather than normal ductal cells. The enriched metabolic pathways, particularly cholesterol metabolism and aerobic glycolysis, indicate significant metabolic alterations consistent with malignant transformation. The presence of GPRC5A, a retinoic acid-inducible protein, along with other epithelial markers, supports a ductal origin but with altered differentiation state.

**Validation**: SOX9, HNF1B, FOXA2

**Metastatic PDAC Cells (score: 45)**

Invasive state with enhanced mobility

**Key Markers**:

- Cell-type-specific: MSLN, EPCAM, KRT19
- Cell-state-specific: LAMB3, S100 proteins, SPINT2

**Evidence and Reasoning**

The expression of MSLN combined with LAMB3 suggests an invasive phenotype characteristic of metastatic cells. The maintained expression of epithelial markers (EPCAM, KRT19) indicates epithelial origin, while the presence of S100 family proteins suggests enhanced mobility and invasion potential. The enriched cholesterol metabolism pathway is particularly relevant, as altered lipid metabolism often supports metastatic progression. The combination of these markers with the metabolic profile suggests cells that have maintained core PDAC identity while acquiring features supporting metastatic behavior.

**Validation**: SNAI1, TWIST1, ZEB1

**Novel Insights**

The gene expression profile reveals an interesting interplay between epithelial identity maintenance and malignant transformation. The strong presence of multiple S100 family members (S100P, S100A6, S100A11, S100A14) suggests a complex calcium-dependent signaling network potentially driving PDAC progression. The enrichment of both cholesterol metabolism and aerobic glycolysis pathways indicates a sophisticated metabolic reprogramming that goes beyond the classical Warburg effect. The co-expression of MSLN with epithelial markers might represent a novel mechanism for maintaining cellular plasticity in PDAC, potentially enabling rapid adaptation to changing tumor microenvironment conditions.

**cluster geneset 4**

**Gene List**

Top genes

| Plain Text PCSK1N,SCG5,CHGA,SCGN,TTR,ERO1LB,NEUROD1,SCG3,GNAS,SLC30A8,CPE,NKX2-2,CHGB,ABCC8,INS,PTPRN,GAD2,MIR7-3HG,PCSK2,MEG3,SYT7,FAM159B,UCHL1,PCSK1,BEX1,HADH,G6PC2,PAX6,IAPP,FAM105A,KIAA1324,SNAP25,BEX2,PAM,RASD1,MLXIPL,ASB9,ADCYAP1,MEIS2,PFN2,APLP1,SYT13,PARVB,SEZ6L2,LINC00948,PFKFB2,SCG2,ELAVL4,INSM1,QPCT |
| --- |

enrichment pathway

| Plain Text  - WikiPathways_2024_Human: Synaptic Vesicle Pathway ,Cell Lineage Map For Neuronal Differentiation ,Splicing Factor NOVA Regulated Synaptic Proteins ,NRXN1 Deletion Syndrome ,GLP 1 From Intestine And Pancreas Role In Glucose Homeostasis |
| --- |

**celltype thinking**

| Plain Text Hmm, fascinating gene signature here... Let me dive deep into this PDAC context. First, those genes are really jumping out at me - CHGA, CHGB, SCG5, NEUROD1... classic neuroendocrine markers. And look at those hormones - INS, IAPP...   Wait a minute, this is really interesting in PDAC context. The pancreas has both exocrine and endocrine components. These genes are screaming pancreatic endocrine identity. But need to be careful here - could be either normal endocrine cells caught in the tumor or tumor cells with neuroendocrine differentiation.  Looking at the pathway enrichment... synaptic vesicle pathway, neuronal differentiation... makes perfect sense given the secretory nature of endocrine cells. They use similar machinery for hormone secretion as neurons use for neurotransmitter release.  Let me trace the developmental aspects... NEUROD1, PAX6, INSM1 - these are key transcription factors in pancreatic endocrine development. NKX2-2 is particularly interesting - it's crucial for beta cell specification.  The hormone processing machinery is all here too - PCSK1, PCSK2, CPE... These are essential for prohormone processing. And look at the vesicle-related genes - SYT7, SNAP25, SCG2, SCG3... classic components of the regulated secretory pathway.  Checking against the candidate cell types... The ductal markers aren't prominent here, despite this being PDAC. Instead, we've got this strong endocrine signature. Could these be:  1. Pancreatic beta cells - the INS, IAPP, SLC30A8, ABCC8 combination is pretty specific 2. Neuroendocrine tumor cells - given the PDAC context and broad neuroendocrine program 3. Other pancreatic endocrine cells - some markers suggest non-beta endocrine identity too  Need to think about validation... For beta cells, PDX1 and UCN3 would be good independent markers. For neuroendocrine identity, ASCL1 and SYP would be useful. For general endocrine function, CHRNB2 and SST would help confirm.  The pathway analysis really supports this - GLP1 pathway is classic beta cell biology. The synaptic/neuronal pathways align perfectly with the secretory machinery these cells need.  Looking at exclusion criteria... No strong markers for other cell types like immune cells or fibroblasts. The pathway patterns are coherent with endocrine function. The combination of markers makes biological sense in pancreatic context.  This is really fascinating - could be capturing either residual normal islet cells or tumor cells with significant endocrine differentiation. The distinction would need additional context about spatial organization and malignant markers... |
| --- |

Based on comprehensive analysis of the gene signature, pathway enrichment, and biological context, the three most likely cell types are:

1. Pancreatic Beta Cells

- Key markers: INS, IAPP, SLC30A8, ABCC8, G6PC2
- Functional signature: Hormone processing (PCSK1/2, CPE) and secretory machinery (SYT7, SNAP25)
- Developmental regulators: NEUROD1, NKX2-2, PAX6
- Validation markers: PDX1, UCN3, MAFA

1. Neuroendocrine-like Tumor Cells

- Key markers: CHGA, CHGB, SCG3, SCG5
- Functional signature: Regulated secretory pathway components
- Developmental regulators: INSM1, NEUROD1
- Validation markers: ASCL1, SYP, NCAM1

1. Pancreatic Endocrine Cells (non-beta)

- Key markers: CHGA, SCG5, PCSK1N
- Functional signature: Hormone processing machinery
- Developmental regulators: NEUROD1, PAX6
- Validation markers: ARX, IRX2, SST

**Score**

| Plain Text Let me carefully evaluate each predicted cell type using the scoring criteria with pathways since we have pathway information...  Starting with Pancreatic Beta Cells: Marker profile analysis... Strong specific markers here - INS, IAPP, SLC30A8, ABCC8 are highly specific beta cell markers. G6PC2, PCSK1/2 are also characteristic. NKX2-2, NEUROD1, PAX6 are key beta cell transcription factors. This looks like a very complete beta cell signature. No negative markers present.  Pathway evaluation... GLP1 pathway is absolutely classic for beta cells - that's a direct hit. The synaptic vesicle pathway makes perfect sense for insulin secretion machinery. Neuronal differentiation pathway aligns with endocrine development. No conflicting pathways.  Context consideration... Beta cells are completely plausible in PDAC context - they're native pancreatic cells that could be captured in tumor samples.  Moving to Neuroendocrine-like Tumor Cells: Marker profile... CHGA, CHGB, SCG3, SCG5 are strong neuroendocrine markers. INSM1, NEUROD1 support this identity. However, these overlap somewhat with normal endocrine cells, so need to deduct some points for shared markers.  Pathway analysis... The synaptic/secretory pathways fit well with neuroendocrine function. Neuronal differentiation pathway is relevant. No direct conflicts.  Context... Very plausible in PDAC - neuroendocrine differentiation is a known phenomenon in pancreatic cancer.  For Pancreatic Endocrine Cells (non-beta): Marker profile... CHGA, SCG5, PCSK1N are present but less specific than the beta cell markers. Shares many markers with both other categories - significant overlap.  Pathway analysis... Similar pathway matches to the other categories, but less specific fit than beta cells.  Context... Plausible in PDAC context but less compelling given the strong beta cell signature.  Calculating final scores...  Beta Cells: Markers: 45 (strong specific markers) + 15 (narrow markers) = 60 Pathways: 15 (state) + 5 (type) = 20 Context: 10 (type) + 10 (state) = 20 Total: 100  Neuroendocrine-like Tumor: Markers: 40 (markers present) + 10 (narrow) - 10 (shared) = 40 Pathways: 15 (state) + 5 (type) = 20 Context: 10 (type) + 10 (state) = 20 Total: 80  Pancreatic Endocrine: Markers: 35 (markers present) + 5 (narrow) - 10 (shared) = 30 Pathways: 10 (state) + 5 (type) - 10 (shared) = 5 Context: 10 (type) + 10 (state) = 20 Total: 55 |
| --- |

Pancreatic Beta Cells: 100

Neuroendocrine-like Tumor Cells: 80

Pancreatic Endocrine Cells: 55

**Report**

**Celltype Prediction**

**Pancreatic Beta Cells (score: 100)**

Active hormone-secreting state with high insulin production capacity

**Key Markers**:

- Cell-type-specific: INS, IAPP, SLC30A8, ABCC8, G6PC2
- Cell-state-specific: PCSK1, PCSK2, CPE, SYT7, SNAP25

**Evidence and Reasoning**

The cell identity is strongly supported by the presence of definitive beta cell markers including insulin (INS) and islet amyloid polypeptide (IAPP), along with crucial glucose-sensing machinery (SLC30A8, ABCC8). The transcriptional program is regulated by key beta cell transcription factors (NEUROD1, NKX2-2, PAX6) that maintain cell identity. The enriched GLP1 and synaptic vesicle pathways perfectly align with the specialized secretory machinery required for insulin release. The presence of prohormone convertases (PCSK1/2) and carboxypeptidase E (CPE) indicates active hormone processing. This molecular profile represents mature, functional beta cells capable of regulated insulin secretion, which can be found within or adjacent to PDAC tissue.

**Validation**: PDX1, UCN3, MAFA, NKX6-1

**Neuroendocrine-like Tumor Cells (score: 80)**

Differentiated neuroendocrine state with active secretory machinery

**Key Markers**:

- Cell-type-specific: CHGA, CHGB, SCG3, SCG5
- Cell-state-specific: INSM1, NEUROD1, SYT7, SNAP25

**Evidence and Reasoning**

The strong expression of chromogranin proteins (CHGA, CHGB) and secretogranins (SCG3, SCG5) establishes a clear neuroendocrine identity. The presence of INSM1 and NEUROD1 indicates active maintenance of the neuroendocrine transcriptional program. The enriched synaptic vesicle and neuronal differentiation pathways support the specialized secretory apparatus characteristic of neuroendocrine cells. This profile is particularly relevant in PDAC context, where tumor cells can undergo neuroendocrine differentiation. The combination of hormone processing machinery (PCSK1/2) with vesicle-associated proteins (SYT7, SNAP25) suggests these cells maintain active peptide hormone secretion capabilities.

**Validation**: ASCL1, SYP, NCAM1, DLL3

**Pancreatic Endocrine Cells (score: 55)**

Active hormone-secreting state with mixed endocrine identity

**Key Markers**:

- Cell-type-specific: CHGA, SCG5, PCSK1N
- Cell-state-specific: NEUROD1, PAX6, CPE

**Evidence and Reasoning**

The expression profile indicates a general pancreatic endocrine identity, marked by chromogranin A (CHGA) and secretogranin V (SCG5). The presence of key transcription factors NEUROD1 and PAX6 supports endocrine differentiation, while hormone processing enzymes like CPE indicate active hormone production. The enriched synaptic vesicle and neuronal differentiation pathways align with the secretory nature of these cells. However, the lack of specific hormone markers for non-beta endocrine cells (like glucagon or somatostatin) makes this a less specific designation. These cells likely represent a mixed population of pancreatic endocrine cells captured within the PDAC microenvironment.

**Validation**: ARX, IRX2, SST, GCG

**Novel Insights**

The gene signature reveals a remarkable preservation of the complex hormone processing and secretory machinery in these cells within the PDAC context. The presence of both beta cell-specific and general neuroendocrine markers suggests potential plasticity between endocrine cell states in the tumor microenvironment. The enrichment of neuronal differentiation pathways alongside hormone processing machinery highlights the sophisticated regulatory networks maintaining endocrine cell function. This could provide new insights into both normal endocrine cell biology and the phenomenon of neuroendocrine differentiation in pancreatic cancer, potentially offering therapeutic targets for PDAC treatment.

**cluster geneset 5**

**Gene List**

Top genes

| Plain Text PLVAP,RAMP2,GNG11,EPAS1,A2M,CLEC14A,SPRY1,AC011526.1,HLA-E,SPARCL1,TGFBR2,SDPR,RAMP3,HYAL2,EMCN,GSN,EGFL7,FABP5,ENG,CRIP2,NPDC1,SLCO2A1,VWF,TMEM88,ELTD1,RDX,FKBP1A,SLC9A3R2,PPAP2A,IFI27,HSPG2,CD93,COL15A1,TCF4,GIMAP7,ECSCR,CD320,S1PR1,ESAM,RNASE1,AQP1,GIMAP4,IGFBP4,CYYR1,CALCRL,RGCC,STOM,TIMP3,JAM2,CAV1 |
| --- |

enrichment pathway

| Plain Text  - WikiPathways_2024_Human: VEGFA VEGFR2 Signaling ,Cancer Pathways ,EGF EGFR Signaling ,Ras Signaling ,Gastrin Signaling |
| --- |

**celltype thinking**

| Plain Text Hmm, looking at this gene signature from PDAC tumor context... Let me dive deep into these markers and pathways...  PLVAP, VWF, EMCN jumping out immediately - classic endothelial markers. And RAMP2/CALCRL system is crucial for vascular homeostasis. This strongly points to endothelial identity.  The pathway enrichment is fascinating - VEGFA-VEGFR2 signaling is the master regulator of angiogenesis. In PDAC context, this makes perfect sense as tumors need extensive vascularization. The presence of EPAS1 (HIF-2α) suggests these cells are responding to the hypoxic tumor microenvironment.  Looking deeper at the gene list... CLEC14A, ENG, CD93 - more endothelial-specific markers. SPARCL1 and HSPG2 indicate basement membrane interaction. The presence of CAV1 and TIMP3 suggests these are mature vessels rather than tip cells.  But wait, need to consider alternatives... Could these be cancer cells undergoing endothelial mimicry? No, the signature is too "purely" endothelial without epithelial markers. What about pericytes? No, missing canonical markers like PDGFRB or RGS5.  The immune-related genes like HLA-E, GIMAP4/7 are interesting... Could reflect immune cell interaction rather than identity. This fits with the tumor context where endothelial cells actively participate in immune regulation.  Looking at the candidate list... The markers align perfectly with endothelial cells, particularly the quiescent state markers like CLDN5 and TIE1. But the pathway analysis suggests these aren't completely quiescent - they show signs of active remodeling.  Need to think about PDAC-specific aspects... These endothelial cells appear to be mature but plastic, showing both stabilizing factors (S1PR1, TIMP3) and angiogenic potential (VEGFR2 pathway). This matches what we know about tumor vasculature.  Other possibilities? The presence of A2M and GSN could suggest fibroblasts, but the overall signature is overwhelmingly endothelial. Cancer cells themselves are ruled out by lack of epithelial markers and the strong vascular program.  The combination of specific markers, coherent pathway enrichment, and biological context makes endothelial cells the most confident prediction. The state appears to be a tumor-associated endothelial phenotype, balanced between stability and plasticity.  For validation, I'd look for additional markers not in this set - CDH5 (VE-cadherin), TEK (Tie2), or PECAM1 (CD31) would provide solid confirmation. |
| --- |

Based on comprehensive analysis of the markers, pathways, and PDAC context, here are the three most likely cell types in order of probability:

1. Endothelial Cells (Highest Confidence)

- Key Markers Present: PLVAP, VWF, CLEC14A, ENG, CD93, EMCN
- Supporting Pathways: VEGFA-VEGFR2 signaling
- Biological Context: Tumor vasculature in PDAC
- Validation Markers: CDH5, TEK, PECAM1
- State: Tumor-associated endothelial cells showing both stable and plastic features

1. Pericytes (Lower Probability)

- Key Markers Present: CAV1, TIMP3
- Supporting Evidence: Vascular association genes
- Biological Context: Vessel-associated cells in PDAC
- Validation Markers: PDGFRB, RGS5, ACTA2
- State: Vessel-supportive cells

1. Fibroblasts (Lowest Probability)

- Key Markers Present: A2M, GSN
- Supporting Pathways: EGF-EGFR signaling
- Biological Context: Stromal component of PDAC
- Validation Markers: FAP, PDPN, COL1A1
- State: Tumor-associated stromal cells

The evidence overwhelmingly supports an endothelial cell identity, with other possibilities being significantly less likely based on the overall marker profile and pathway analysis.

**Score**

| Plain Text Let me carefully score each predicted cell type based on the provided criteria and pathway information...  Starting with Endothelial Cells: Marker Profile analysis... Strong endothelial-specific markers: PLVAP, VWF, CLEC14A, ENG, CD93, EMCN - these are highly specific narrow markers, worth significant points. RAMP2/CALCRL system is also quite specific. Looking at about 40-45 points for matching markers. No negative markers present, that's good. Some markers like CAV1 are shared with other cell types but not enough to significantly penalize.  Pathway analysis for endothelial cells: VEGFA-VEGFR2 signaling - perfect match for both cell type and state in endothelial cells, full 20 points here. Other pathways like EGF-EGFR are shared but contextually relevant.  Biological context: Endothelial cells are absolutely expected in PDAC tumor context, and their state matches tumor vasculature requirements. Full 20 points.  For Pericytes: Much weaker marker profile - only CAV1 and TIMP3 are somewhat relevant but not specific. Maybe 15-20 points. Many markers shared with endothelial cells, needs penalty. Pathways aren't specifically pericyte-related. Context is plausible but not strongly supported.  For Fibroblasts: Very weak marker evidence - A2M and GSN are quite general. Missing key fibroblast markers. Pathways aren't fibroblast-specific. While context is plausible, evidence is minimal.  Let me calculate final scores... Endothelial: ~45(markers) + 20(pathways) + 20(context) = 85 Pericytes: ~15(markers) - 10(shared) + 5(pathways) + 10(context) = 20 Fibroblasts: ~10(markers) - 10(shared) + 0(pathways) + 10(context) = 10 |
| --- |

Endothelial Cells: 85

Pericytes: 20

Fibroblasts: 10

**Report**

**Celltype Prediction**

**Endothelial Cells (score: 85)**

Tumor-associated endothelial cells showing balanced phenotype between vascular stability and angiogenic plasticity

**Key Markers**:

- Cell-type-specific: PLVAP, VWF, CLEC14A, ENG, CD93, EMCN
- Cell-state-specific: EPAS1, SPARCL1, TIMP3, S1PR1, RGCC

**Evidence and Reasoning**

The cell identity is strongly supported by multiple endothelial-specific markers including PLVAP, VWF, and CLEC14A, which are canonical endothelial cell markers. The VEGFA-VEGFR2 signaling pathway enrichment provides strong evidence for endothelial identity and function. The expression of EPAS1 (HIF-2α) indicates adaptation to the hypoxic tumor microenvironment. The presence of both stabilizing factors (S1PR1, TIMP3) and angiogenic molecules suggests a dynamic state balancing vascular stability with plasticity. The RAMP2/CALCRL system, crucial for vascular homeostasis, further supports this identity. The expression of basement membrane interactors (SPARCL1, HSPG2) and mature vessel markers (CAV1) indicates established vessel formation rather than tip cells, consistent with tumor vasculature characteristics.

**Validation**: CDH5 (VE-cadherin), TEK (Tie2), PECAM1 (CD31)

**Pericytes (score: 20)**

Vessel-associated supportive cells

**Key Markers**:

- Cell-type-specific: CAV1
- Cell-state-specific: TIMP3, GSN

**Evidence and Reasoning**

The presence of CAV1 and TIMP3 suggests possible pericyte identity, as these proteins are important for vessel stabilization and maintenance. The enrichment of VEGFA-VEGFR2 signaling pathway could indicate involvement in vessel maturation processes. However, the evidence is limited by the absence of classical pericyte markers. The expression pattern could represent pericytes in a tumor-modified state, as PDAC often shows altered vessel architecture. The presence of these markers might also reflect the close physical and functional association between pericytes and endothelial cells in tumor vasculature, though the overall signature more strongly supports an endothelial identity.

**Validation**: PDGFRB, RGS5, ACTA2, NG2

**Fibroblasts (score: 10)**

Tumor-associated stromal cells

**Key Markers**:

- Cell-type-specific: A2M
- Cell-state-specific: GSN, TIMP3

**Evidence and Reasoning**

The presence of A2M and GSN provides weak evidence for fibroblast identity, as these markers are expressed in multiple cell types including stromal cells. The EGF-EGFR signaling pathway enrichment could support a fibroblast identity given their role in tumor-stroma interactions. However, the overall marker profile lacks definitive fibroblast-specific markers. While fibroblasts are a major component of the PDAC microenvironment, the absence of characteristic fibroblast markers and the predominance of endothelial-specific markers makes this the least likely identity. The observed markers might instead reflect the stromal-vascular interactions common in tumor microenvironments.

**Validation**: FAP, PDPN, COL1A1, THY1

**Novel Insights**

The gene signature reveals a sophisticated adaptation of tumor vasculature in PDAC. The balanced expression of both stabilizing factors (TIMP3, S1PR1) and plasticity markers suggests a unique vascular state that might be crucial for tumor progression. The presence of immune-regulatory molecules (HLA-E) and stress response factors (EPAS1) indicates these endothelial cells actively participate in immune modulation and hypoxia response. This dual functionality might represent a specialized adaptation to the PDAC microenvironment, potentially offering new therapeutic targets for anti-angiogenic strategies. The expression pattern suggests these vessels maintain structural integrity while retaining the ability to respond to tumor-derived angiogenic signals, a finding that could impact our understanding of PDAC vascularization strategies.

**cluster geneset 6**

**Gene List**

Top genes

| Plain Text LUM,DCN,COL1A2,COL6A3,C1S,SFRP2,FBLN1,COL3A1,RARRES2,COL6A2,C1R,CCDC80,SERPINF1,COL1A1,MMP2,AEBP1,BGN,LGALS1,PCOLCE,CTSK,CALD1,APOD,CTHRC1,COL6A1,SPARC,VCAN,FN1,NBL1,NNMT,MXRA8,FSTL1,THY1,CLEC11A,COL5A2,MGP,ISLR,TPM2,TIMP1,THBS2,HTRA1,ASPN,MFAP4,TAGLN,VIM,SELM,SPON2,MEG3,EMILIN1,PALLD,MYL9 |
| --- |

enrichment pathway

| Plain Text  - WikiPathways_2024_Human: miRNA Targets In ECM And Membrane Receptors ,Endochondral Ossification With Skeletal Dysplasias ,Endochondral Ossification ,Focal Adhesion ,Type I Collagen Synthesis Osteogenesis Imperfecta |
| --- |

**celltype thinking**

| Plain Text Hmm, looking at this gene signature from cluster 6 in PDAC context... Let me think this through carefully...  The gene signature is striking - heavy ECM and matrix components. LUM, DCN, multiple collagens (COL1A1, COL1A2, COL3A1, COL6A1/2/3)... This immediately points to cells involved in matrix production and remodeling.  In PDAC specifically, the desmoplastic reaction is a hallmark feature. The stroma can comprise up to 90% of tumor mass in some cases. Need to consider the major stromal players...  Cancer-associated fibroblasts (CAFs) are the prime suspect here. Looking at canonical CAF markers - yes, we have ACTA2/TAGLN suggesting myofibroblastic phenotype. FN1, SPARC, THY1 (CD90) are all classic CAF markers. The extensive ECM signature fits perfectly with CAF biology.  But wait - could these be stellate cells? Pancreatic stellate cells (PSCs) are the major source of CAFs in PDAC. When activated, PSCs express many of these same markers. The distinction between activated PSCs and CAFs can be quite blurry... Need to think about their relationship.  The pathway enrichment supports this - focal adhesion, ECM interactions, collagen synthesis. All hallmarks of activated fibroblasts/stellate cells.  What about other possibilities? Seeing VIM, which could suggest EMT in tumor cells... but the overall signature is too strongly stromal. Regular fibroblasts? Possible, but in PDAC context, they're likely to be activated/CAF-like.  CTHRC1 is interesting - it's induced during tissue repair and can promote migration. MMP2 shows active matrix remodeling. TIMP1 suggests regulated ECM turnover. This is a very "active" stromal signature.  Looking at the full marker panel - no strong immune markers, no endothelial signatures. This really narrows it down to stromal populations.  The presence of mechanosensing/contractile proteins like TAGLN, MYL9, CALD1 strongly suggests myofibroblastic phenotype. This is characteristic of activated PSCs/CAFs in PDAC.  Let me validate against known negative markers... No epithelial markers (except some VIM which is actually expected in stromal cells), no immune markers, no endothelial markers. This helps rule out other major cell types.  Circling back to the PDAC context - this signature perfectly matches what we know about PDAC stroma: extensive desmoplasia, activated myofibroblastic cells, active ECM remodeling.  After this deep analysis, I'm confident in identifying the most likely cell types... |
| --- |

Based on comprehensive analysis of the gene signature, pathway enrichment, and PDAC biological context, the three most likely cell types are:

1. Cancer-Associated Fibroblasts (CAFs)

- Key markers present: THY1, TAGLN, ACTA2, FN1, SPARC
- Extensive ECM production signature: Multiple collagens, LUM, DCN
- Active ECM remodeling: MMP2, TIMP1, CTHRC1
- Validation markers not in signature: FAP, PDGFRα, FSP1

1. Activated Pancreatic Stellate Cells (aPSCs)

- Overlapping markers with CAFs: ACTA2, TAGLN, ECM components
- Characteristic ECM production profile
- Contractile phenotype markers: MYL9, CALD1
- Validation markers not in signature: GFAP, DES, NESTIN

1. Myofibroblasts

- Contractile protein expression: TAGLN, MYL9, CALD1
- ECM production capacity
- Mechanosensing properties
- Validation markers not in signature: PDGFRB, TCF21

These populations likely represent a spectrum of activated stromal cells in PDAC, with significant overlap in their molecular profiles but distinct developmental origins and functions in the tumor microenvironment.

**Score**

| Plain Text Let me carefully evaluate each predicted cell type using the scoring criteria with pathway since we have pathway information...  Starting with CAFs: Marker profile analysis... - Strong matching markers: THY1, TAGLN, ACTA2, FN1, SPARC are classical CAF markers (~40 pts) - Narrow markers: ECM components like specific collagens and matrix proteins are characteristic (~12 pts) - Some marker overlap with myofibroblasts and aPSCs, but this is biologically expected (-10 pts) - No negative markers present (0 pts)  Pathway analysis... - ECM and focal adhesion pathways perfectly match CAF state (15 pts) - These pathways also match CAF cell type identity (5 pts) - Some pathway overlap with other stromal cells (-10 pts)  Context analysis... - CAFs are absolutely expected in PDAC (10 pts) - Activated state matches PDAC context (10 pts)  For aPSCs: Marker profile... - Good matching markers: ACTA2, TAGLN, ECM components (~35 pts) - Fewer narrow specific markers compared to CAFs (~8 pts) - Significant overlap with CAFs/myofibroblasts (-10 pts) - No negative markers (0 pts)  Pathway analysis... - ECM pathways match activated state (15 pts) - Pathways match cell type (5 pts) - Significant pathway overlap (-10 pts)  Context analysis... - PSCs are key players in PDAC (10 pts) - Activated state matches context (10 pts)  For Myofibroblasts: Marker profile... - Present markers: TAGLN, MYL9, CALD1 (~30 pts) - Limited narrow specific markers (~5 pts) - Heavy overlap with CAFs/PSCs (-10 pts) - No negative markers (0 pts)  Pathway analysis... - Pathways match contractile state (15 pts) - General match to cell type (5 pts) - Significant overlap (-10 pts)  Context analysis... - Present in PDAC but less specific (5 pts) - Activated state matches context (10 pts)  Adding up the scores... |
| --- |

Cancer-Associated Fibroblasts (CAFs): 72

Activated Pancreatic Stellate Cells (aPSCs): 63

Myofibroblasts: 50

**Report**

**Celltype Prediction**

**Cancer-Associated Fibroblasts (CAFs) (score: 72)**

Activated and matrix-remodeling state

**Key Markers**:

- Cell-type-specific: THY1, SPARC, FN1
- Cell-state-specific: ACTA2, TAGLN, MMP2, TIMP1, CTHRC1

**Evidence and Reasoning**

The strong expression of ECM components (COL1A1, COL1A2, COL3A1, COL6A1/2/3) combined with matrix remodeling enzymes (MMP2, TIMP1) strongly indicates CAFs. The presence of myofibroblastic markers (ACTA2, TAGLN) and mechanosensing proteins (MYL9, CALD1) suggests an activated phenotype. Enriched pathways in focal adhesion and ECM organization support CAF identity. The extensive desmoplastic reaction characteristic of PDAC is consistent with abundant CAF presence. The co-expression of contractile elements (TAGLN, MYL9) with matrix proteins (SPARC, FN1) reflects CAFs' dual role in ECM production and mechanical force generation. CTHRC1 expression suggests active involvement in tissue remodeling and potential tumor-promoting functions.

**Validation**: FAP, PDGFRα, FSP1

**Activated Pancreatic Stellate Cells (aPSCs) (score: 63)**

Myofibroblast-like activated state

**Key Markers**:

- Cell-type-specific: ACTA2, TAGLN
- Cell-state-specific: COL1A1, COL1A2, MMP2, TIMP1

**Evidence and Reasoning**

The signature shows hallmarks of activated PSCs, including strong expression of contractile proteins (ACTA2, TAGLN) and extensive ECM production (multiple collagens, LUM, DCN). The enrichment of focal adhesion and ECM-related pathways aligns with PSC activation. In PDAC, quiescent PSCs transform into activated myofibroblast-like cells, which is reflected in this gene signature. The presence of matrix remodeling factors (MMP2, TIMP1) and mechanosensing proteins (MYL9, CALD1) indicates active participation in stromal remodeling. The expression profile suggests these cells are major contributors to the desmoplastic reaction characteristic of PDAC, though there is significant overlap with CAF markers due to their similar activated phenotypes.

**Validation**: GFAP, DES, NESTIN

**Myofibroblasts (score: 50)**

Contractile activated state

**Key Markers**:

- Cell-type-specific: TAGLN, MYL9, CALD1
- Cell-state-specific: ACTA2, COL1A1, COL1A2

**Evidence and Reasoning**

The expression profile shows strong enrichment for contractile proteins (TAGLN, MYL9, CALD1) characteristic of myofibroblasts. The presence of ECM components and enrichment of focal adhesion pathways supports their role in tissue remodeling. In PDAC context, these cells likely represent a broader population of activated stromal cells with contractile properties. The combination of mechanical and matrix-related proteins suggests active participation in tissue mechanics and architecture. However, the significant overlap with CAF and PSC markers makes it challenging to distinguish these as a completely separate population, as they may represent a functional state rather than a distinct cell type.

**Validation**: PDGFRB, TCF21

**Novel Insights**

The gene signature reveals a complex stromal state in PDAC characterized by simultaneous expression of contractile and matrix-remodeling programs. The presence of CTHRC1 alongside traditional ECM components suggests active matrix reorganization rather than mere deposition. The co-expression of mechanosensing proteins (MYL9, CALD1) with matrix remodeling factors (MMP2, TIMP1) indicates a mechanically responsive stromal population that may dynamically modify the tumor microenvironment. This suggests potential mechanical feedback loops between stromal cells and their surrounding matrix, which could be crucial for PDAC progression and therapy resistance.

**cluster geneset 7**

**Gene List**

Top genes

| Plain Text TYROBP,FCER1G,AIF1,GPX1,HLA-DRA,HLA-DPA1,FTL,NPC2,HLA-DRB1,LST1,LAPTM5,HLA-DPB1,CTSS,CYBA,ITGB2,CD74,SRGN,C1orf162,HLA-DQB1,SPI1,CD68,TYMP,MS4A6A,HLA-DQA1,SAT1,LYZ,COTL1,GRN,PSAP,HLA-DMA,CAPG,CST3,CTSB,MNDA,RGS10,RGS1,OAZ1,HCST,SH3BGRL3,MS4A7,ALOX5AP,S100A4,ARPC3,FTH1,CD14,CD53,LSP1,TMSB4X,CSTA,FCGR2A |
| --- |

enrichment pathway

| Plain Text  - WikiPathways_2024_Human: TYROBP Causal Network In Microglia ,Microglia Pathogen Phagocytosis Pathway ,Chemokine Signaling ,Ebola Virus Infection In Host ,Type II Interferon Signaling |
| --- |

**celltype thinking**

| Plain Text Hmm, looking at this gene signature from cluster 7 in PDAC context... Let me dive deep into the molecular patterns here.  TYROBP and FCER1G immediately catch my attention - these are key adaptors in myeloid cells. And there's AIF1 (also known as IBA1), that's a classic microglial/macrophage marker. The strong presence of HLA class II genes (HLA-DRA, DPA1, DPB1, DQB1, DQA1) points to professional antigen-presenting cells...  The pathway enrichment is really telling - TYROBP Causal Network and Microglia Phagocytosis Pathway. In pancreatic context though, we're not talking about microglia, but rather tissue-resident macrophages and infiltrating myeloid cells.  Looking at the full gene signature... CD68, CD14, FCGR2A - these are definitive macrophage markers. The presence of phagocytosis-related genes like CYBA, ITGB2, and lysosomal components (CTSB, CTSS) fits perfectly with macrophage function.  But I need to be careful here - could these genes also fit dendritic cells? DCs do express HLA class II and some of these markers. However, the strong expression of CD68 and CD14, combined with TYROBP/FCER1G and phagocytic machinery, more strongly supports macrophages.  The SPI1 (PU.1) transcription factor is particularly interesting - it's essential for myeloid lineage development. Combined with MS4A6A and MS4A7, this really reinforces the myeloid identity.  Could these be neutrophils? No, while they express some of these genes, the strong HLA class II signature and CD68/CD14 expression rules them out. Plus, neutrophils typically show high S100A8/S100A9, which aren't prominent here.  Looking at the PDAC context, tumor-associated macrophages (TAMs) are known to be abundant and important players. These genes suggest a mixed activation state - some inflammatory markers but also tissue-remodeling factors like CTSB and PSAP.  Let me think about validation markers not in this set... CD163, MRC1 (CD206), and MSR1 would be good additional markers to confirm macrophage identity. For functional validation, phagocytosis assays would be key.  The chemokine signaling pathway enrichment makes sense - macrophages are major producers and responders to chemokines in the tumor microenvironment. The type II interferon signaling suggests some inflammatory activation.  Looking at the Candidate_celltype list, this profile best matches the Macrophage category, particularly showing features of both M1 and M2-like states based on the mixed inflammatory and tissue-remodeling signatures. |
| --- |

Based on comprehensive analysis of the gene signature, pathway enrichment, and biological context, here are the three most likely cell types, ranked by confidence:

1. Tumor-Associated Macrophages (TAMs)

- Primary Evidence:
- Core myeloid markers: CD68, CD14, FCGR2A
- Antigen-presenting machinery: HLA-DRA/DPA1/DPB1/DQB1
- Phagocytic function: CYBA, ITGB2, CTSB
- Myeloid lineage factor: SPI1 (PU.1)
- Validation markers (not in cluster): CD163, MRC1, MSR1

1. Dendritic Cells

- Primary Evidence:
- HLA class II expression
- Antigen processing machinery: CTSS, CD74
- Professional APC markers
- However, less likely due to strong macrophage-specific markers

1. Monocytes

- Primary Evidence:
- CD14, FCGR2A expression
- Basic myeloid markers
- However, the complex tissue-remodeling signature suggests tissue-resident cells rather than circulating monocytes

The gene signature most strongly supports a macrophage identity, specifically tumor-associated macrophages, which is highly consistent with the PDAC tumor microenvironment context.

**Score**

| Plain Text Let me carefully evaluate each predicted cell type using the scoring criteria with pathway since we have pathway information...  Starting with TAMs/Macrophages: Marker Profile analysis... Strong matching markers: CD68 (classic), CD14 (classic), FCGR2A, TYROBP, FCER1G, AIF1 - these are definitive macrophage markers, very high confidence. HLA class II molecules also match but are shared with other APCs. Looking at narrow markers - CD68 and CD14 combination is quite specific. No negative markers present.  Pathway Profile... TYROBP Causal Network and Microglia Phagocytosis Pathway are perfectly aligned with macrophage function. Chemokine signaling and Type II Interferon are also consistent with macrophage biology. No conflicting pathways.  Context analysis... TAMs are well-documented and expected in PDAC microenvironment. The activation state matches tumor context.  For Dendritic Cells: Marker Profile... HLA class II molecules match but are shared with macrophages. CD74, CTSS fit DC biology but aren't specific. Strong macrophage markers like CD68/CD14 somewhat conflict with pure DC identity.  Pathway Profile... Antigen presentation pathways fit, but TYROBP network is less typical for DCs. Some pathway overlap with macrophages reduces specificity.  Context... DCs are plausible in PDAC but profile fits TAMs better.  For Monocytes: Marker Profile... CD14, FCGR2A match but are shared with macrophages. Tissue-remodeling signatures (CTSB, PSAP) don't fit circulating monocytes well.  Pathway Profile... Chemokine signaling fits, but tissue-resident pathways don't match circulating monocytes.  Context... While monocytes can infiltrate tumors, the profile suggests tissue-resident cells.  Let me calculate final scores... TAMs: Markers(55/60) + Pathways(20/20) + Context(20/20) = 95 DCs: Markers(25/60) + Pathways(5/20) + Context(15/20) = 45 Monocytes: Markers(20/60) + Pathways(0/20) + Context(10/20) = 30 |
| --- |

Tumor-Associated Macrophages: 95

Dendritic Cells: 45

Monocytes: 30

**Report**

**Celltype Prediction**

**Tumor-Associated Macrophages (score: 95)**

Mixed M1/M2-like state with enhanced phagocytic and antigen-presenting activity

**Key Markers**:

- Cell-type-specific: CD68, CD14, TYROBP, FCER1G, AIF1, FCGR2A
- Cell-state-specific: HLA-DRA, HLA-DPA1, CTSB, CTSS, CYBA, ITGB2

**Evidence and Reasoning**

The gene signature strongly indicates a macrophage identity through multiple lines of evidence. Core myeloid lineage markers (CD68, CD14, FCGR2A) combined with the TYROBP/FCER1G signaling axis establish the macrophage lineage. The extensive HLA class II machinery (HLA-DRA, DPA1, DPB1, DQB1) indicates active antigen-presenting capacity. The presence of SPI1 (PU.1) transcription factor further confirms myeloid lineage commitment. Pathway analysis reveals enrichment in TYROBP Causal Network and Microglia Phagocytosis Pathway, perfectly aligned with macrophage function. The mixed expression of inflammatory mediators and tissue-remodeling factors (CTSB, PSAP) suggests a complex activation state typical of TAMs in the PDAC microenvironment. Chemokine signaling and Type II Interferon pathway enrichment indicate active involvement in immune response coordination.

**Validation**: CD163, MRC1 (CD206), MSR1, CSF1R

**Dendritic Cells (score: 45)**

Activated antigen-presenting state

**Key Markers**:

- Cell-type-specific: HLA-DRA, CD74, CTSS
- Cell-state-specific: HLA-DPA1, HLA-DPB1, HLA-DQB1

**Evidence and Reasoning**

The extensive HLA class II machinery and antigen processing components suggest professional antigen-presenting cell identity. The presence of CD74 and CTSS indicates active involvement in antigen processing and presentation. However, the strong expression of macrophage-specific markers like CD68 and CD14 makes pure DC identity less likely. The pathway analysis shows enrichment in antigen presentation pathways, but the prominent TYROBP network is more characteristic of macrophages. While DCs are known components of the PDAC microenvironment, the overall molecular profile more strongly supports a macrophage identity with enhanced antigen-presenting capabilities rather than classical DCs.

**Validation**: CD1C, CLEC10A, CCR7, FLT3

**Monocytes (score: 30)**

Inflammatory activation state

**Key Markers**:

- Cell-type-specific: CD14, FCGR2A
- Cell-state-specific: TYROBP, FCER1G

**Evidence and Reasoning**

While basic monocyte markers are present (CD14, FCGR2A), the complex tissue-remodeling signature and extensive HLA class II machinery suggest tissue-resident cells rather than circulating monocytes. The pathway enrichment in tissue-resident functions and complex immune responses is more consistent with differentiated macrophages than monocytes. Although monocytes can infiltrate the tumor microenvironment, the presence of mature macrophage markers and tissue-remodeling factors (CTSB, PSAP) indicates these cells have likely differentiated into tissue-resident macrophages. The chemokine signaling pathway enrichment could support monocyte identity, but the overall profile suggests more mature myeloid cells.

**Validation**: CCR2, CX3CR1, LYZ, S100A8/A9

**Novel Insights**

The gene signature reveals a complex myeloid cell state in PDAC that combines enhanced antigen presentation with active tissue remodeling. The simultaneous expression of TYROBP/FCER1G signaling components with extensive HLA class II machinery suggests these TAMs may serve as a bridge between innate and adaptive immunity. The enrichment of both phagocytic and antigen-presenting pathways indicates these cells might be particularly effective at processing and presenting tumor antigens. This hybrid phenotype could represent a specialized TAM population in PDAC that combines classical macrophage functions with enhanced adaptive immune activation capabilities, potentially influencing both tumor progression and immunotherapy responses.

**cluster geneset 8**

**Gene List**

Top genes

| Plain Text MYL9,CALD1,ADIRF,IGFBP7,TPM2,PDGFRB,TAGLN,ACTA2,RGS5,NOTCH3,NDUFA4L2,PPP1R14A,MAP1B,MFGE8,LHFP,SPARCL1,PLAC9,BGN,SOD3,CSRP2,GPX3,PHLDA1,CRIP1,SELM,MYLK,FRZB,MGP,TINAGL1,VIM,LGALS1,COL6A2,SPARC,COX4I2,IFITM2,IFITM3,PRKCDBP,SEPT4,SEPT7,GJA4,CRISPLD2,COL18A1,C11orf96,HIGD1B,MCAM,COX7A1,ADAMTS1,PTP4A3,COL4A2,NR2F2,MT2A |
| --- |

enrichment pathway

| Plain Text  - WikiPathways_2024_Human: miRNA Targets In ECM And Membrane Receptors ,Focal Adhesion ,miR 509 3P Alteration Of YAP1 ECM Axis ,Focal Adhesion PI3K Akt mTOR Signaling ,Cancer Pathways |
| --- |

**celltype thinking**

| Plain Text Hmm, looking at cluster 8's gene profile... This is quite an interesting set with strong ECM and contractile signatures. Let me analyze this systematically...  First, the biological context is PDAC tumor microenvironment - this immediately brings to mind several key players. The stroma is particularly important in PDAC, known for its dense fibrotic reaction.  Looking at the markers... ACTA2, TAGLN, MYL9, CALD1, TPM2 - this is a very clear smooth muscle/myofibroblast signature. These genes encode contractile proteins and cytoskeletal components. But I need to be careful here - multiple cell types can express these in different contexts.  PDGFRB, NOTCH3, RGS5 are catching my eye... These are classical markers of pericytes and vascular smooth muscle cells. But in PDAC context, cancer-associated fibroblasts (CAFs) also express these. Need to dig deeper...  The ECM signature is striking - BGN, SPARC, COL6A2, COL4A2, MFGE8. This strongly suggests a matrix-producing cell type. In PDAC, activated pancreatic stellate cells are major matrix producers. But again, CAFs share many of these features.  Looking at the pathways... Focal adhesion, ECM-related pathways, PI3K-Akt-mTOR signaling. This fits perfectly with an activated stromal phenotype. These cells are clearly engaged in matrix remodeling and mechanosensing.  VIM expression alongside ACTA2 is interesting... While this could suggest EMT in tumor cells, the overall profile doesn't support this - no evident epithelial markers like KRT19 or EPCAM. More consistent with mesenchymal stromal cells.  The presence of stress response genes (MT2A, SOD3, GPX3) and inflammatory mediators (ADAMTS1) suggests these cells are responding to the tumor microenvironment. This is typical of activated stromal cells in PDAC.  Comparing to candidate cell types... This profile aligns most strongly with fibroblast/stellate cell signatures, particularly in their activated state. But I should also consider pericytes and vascular smooth muscle cells given the marker profile.  Let me think about distinguishing features... Pericytes would typically show stronger expression of MCAM/CD146 (which is present but not dominant here) and definitive markers like CSPG4/NG2 (absent). VSMCs would show more mature smooth muscle markers like MYH11 (absent).  The complete profile best fits activated CAFs/stellate cells, with pericytes as a second possibility, and VSMCs as a third. The combination of contractile machinery, ECM production, and stress response is quintessential CAF behavior in PDAC.  For validation, I'd look for additional markers not in this set - PDPN, FAP for CAFs; CSPG4, ANGPT1 for pericytes; MYH11, CNN1 for VSMCs. This would help definitively distinguish between these possibilities. |
| --- |

Based on comprehensive analysis of the marker profile, pathway enrichment, and PDAC context, the three most likely cell types are:

1. Cancer-Associated Fibroblasts (CAFs)/Activated Pancreatic Stellate Cells

- Key markers present: ACTA2, TAGLN, VIM, ECM components (BGN, SPARC, COL6A2)
- Characteristic pathways: Focal adhesion, ECM remodeling
- Validation markers: FAP, PDPN, THY1

1. Pericytes

- Key markers present: PDGFRB, NOTCH3, RGS5, MCAM
- Associated pathways: PI3K-Akt-mTOR signaling
- Validation markers: CSPG4, ANGPT1, DES

1. Vascular Smooth Muscle Cells (VSMCs)

- Key markers present: ACTA2, TAGLN, MYL9, CALD1
- Related pathways: Focal adhesion, contractile function
- Validation markers: MYH11, CNN1, MYLK

**Score**

| Plain Text Let me carefully evaluate each cell type prediction using the scoring criteria with pathway since we have pathway information...  Starting with CAFs/Activated Stellate Cells: Marker Profile analysis... Strong matching markers: ACTA2, TAGLN, VIM, BGN, SPARC, COL6A2, PDGFRB - these are classical CAF markers in PDAC, especially when activated. Very high specificity here. I'd say around 40/45 for matching markers. For narrow markers - ACTA2 and TAGLN in combination with ECM proteins are quite specific for activated CAFs - about 12/15. Some marker overlap with other mesenchymal cells (VSMCs, pericytes) but this is expected: -10  Pathway Profile... ECM and focal adhesion pathways perfectly match CAF state: 15/15 PI3K-Akt-mTOR relevant to CAF biology: 5/5 Some pathway overlap with other stromal cells: -10  Biological Context... CAFs are absolutely central to PDAC biology: 10/10 Activated state is typical in PDAC: 10/10 Total for CAFs: ~82/100  For Pericytes: Marker Profile... PDGFRB, NOTCH3, RGS5, MCAM present: ~35/45 Less specific narrow markers: ~8/15 Significant marker overlap with CAFs/VSMCs: -10  Pathway Profile... Pathways partially match state: ~10/15 Basic cell type pathways present: 3/5 Shared pathways with other stromal cells: -10  Biological Context... Present in PDAC: 10/10 Activated state plausible: 8/10 Total: ~64/100  For VSMCs: Marker Profile... ACTA2, TAGLN, MYL9, CALD1 present: ~30/45 Limited narrow markers: ~5/15 Significant overlap with CAFs/pericytes: -10  Pathway Profile... State pathways partially match: ~8/15 Basic pathways present: 3/5 Shared pathways: -10  Biological Context... Present in PDAC: 8/10 State plausible: 8/10 Total: ~52/100 |
| --- |

CAFs/Activated Stellate Cells: 82

Pericytes: 64

VSMCs: 52

**Report**

**Celltype Prediction**

**Cancer-Associated Fibroblasts/Activated Stellate Cells (score: 82)**

Activated/Matrix-producing state

**Key Markers**:

- Cell-type-specific: PDGFRB, VIM, SPARC, BGN, COL6A2, COL4A2
- Cell-state-specific: ACTA2, TAGLN, MYL9, CALD1, TPM2

**Evidence and Reasoning**

The cell population exhibits a definitive CAF/activated stellate cell signature through multiple lines of evidence. First, the strong expression of contractile proteins (ACTA2, TAGLN, MYL9) combined with extensive ECM components (SPARC, BGN, COL6A2) is characteristic of activated stromal cells in PDAC. The enrichment of focal adhesion and ECM-related pathways, alongside PI3K-Akt-mTOR signaling, indicates active matrix remodeling and mechanosensing. The presence of stress response genes (MT2A, SOD3) and inflammatory mediators (ADAMTS1) further supports an activated state typical of CAFs in the PDAC microenvironment. This profile aligns perfectly with the known role of CAFs as primary matrix producers and mechanical regulators in pancreatic cancer stroma.

**Validation**: FAP, THY1(CD90), PDPN, POSTN

**Pericytes (score: 64)**

Activated/Stress-responsive state

**Key Markers**:

- Cell-type-specific: PDGFRB, NOTCH3, RGS5, MCAM
- Cell-state-specific: ACTA2, TAGLN, MFGE8, SOD3

**Evidence and Reasoning**

The expression profile shows significant pericyte characteristics, particularly through the presence of canonical markers PDGFRB, NOTCH3, and RGS5. The enrichment of PI3K-Akt-mTOR and focal adhesion pathways is consistent with pericyte biology in tumor vasculature. The expression of stress response genes (SOD3, GPX3) and contractile elements (ACTA2, TAGLN) suggests an activated phenotype responding to the tumor microenvironment. While sharing some markers with CAFs, the presence of MCAM and vascular-associated proteins supports a pericyte identity. This profile is consistent with the known role of pericytes in PDAC vasculature, though some marker overlap with other stromal populations indicates potential plasticity.

**Validation**: CSPG4(NG2), ANGPT1, DES, CD248

**Vascular Smooth Muscle Cells (score: 52)**

Activated/Contractile state

**Key Markers**:

- Cell-type-specific: ACTA2, TAGLN, MYL9, CALD1
- Cell-state-specific: NOTCH3, GJA4, COX4I2, COX7A1

**Evidence and Reasoning**

The expression profile shows characteristics of VSMCs through the presence of contractile machinery components (ACTA2, TAGLN, MYL9, CALD1). The enrichment of focal adhesion pathways and expression of vascular-associated genes (GJA4, NOTCH3) supports a VSMC identity. The presence of metabolic regulators (COX4I2, COX7A1) and stress response elements suggests an activated phenotype adapting to the tumor environment. While these features are consistent with VSMCs, the substantial overlap with CAF and pericyte markers, combined with the absence of some classic VSMC markers, makes this a less definitive match compared to the other cell types.

**Validation**: MYH11, CNN1, SMTN, MYLK

**Novel Insights**

The gene expression profile reveals an interesting stromal cell population with significant plasticity, suggesting potential transdifferentiation between different mesenchymal cell states in PDAC. The strong stress response signature (MT2A, SOD3, GPX3) combined with active ECM remodeling pathways indicates these cells may play a crucial role in the tumor's mechanical and oxidative stress responses. The presence of both contractile and matrix-producing characteristics suggests these cells might represent a hybrid state, potentially able to transition between different stromal functions based on microenvironmental cues. This plasticity could be therapeutically relevant for targeting the PDAC stroma.

**cluster geneset 9**

**Gene List**

Top genes

| Plain Text PTPRCAP,CD52,CD3D,ARHGDIB,CORO1A,CD2,CD3E,CXCR4,RAC2,IL32,PTPRC,HLA-C,B2M,CYTIP,GMFG,LDHB,IL2RG,HCST,CD7,EVL,SRGN,LTB,LAPTM5,LSP1,CD53,S100A4,BTG1,LCK,CD37,IL7R,CD3G,ISG20,CD48,PFN1,TMSB4X,HLA-A,ACAP1,PIK3IP1,GPSM3,CD69,FYB,HLA-E,LIMD2,ZFP36L2,EEF1D,ICAM3,TRAF3IP3,KLRB1,CD27,UBA52 |
| --- |

enrichment pathway

| Plain Text  - WikiPathways_2024_Human: Cytoplasmic Ribosomal Proteins ,Modulators Of TCR Signaling And T Cell Activation ,Extrafollicular And Follicular B Cell Activation By SARS CoV 2 ,T Cell Activation SARS CoV 2 ,T Cell Receptor Signaling |
| --- |

**celltype thinking**

| Plain Text Hmm, looking at this PDAC tumor context and the gene signature... Let me dive deep into this.  First pass at the genes - striking enrichment of immune markers. PTPRC (CD45) immediately jumps out as the pan-immune marker. CD3D, CD3E, CD3G - that's the complete CD3 complex, absolutely crucial for T cell receptor signaling.   The pathway analysis really supports this - TCR signaling pathways are prominently enriched. This is starting to paint a clear picture of T cells, but let me be thorough and consider all possibilities...  Looking at other immune markers - CD52, CD2, CD7... these could potentially be NK cells too since they share some markers with T cells. But the complete CD3 complex really points to T cells specifically. NK cells typically express CD56/NCAM1 which I'm not seeing here.  B cell markers? CD37 is present but the really definitive B cell markers like CD19, CD79A/B are absent. The B cell activation pathway might be picking up shared immune signaling components.  What about the T cell state? Seeing IL7R, CD69 - these suggest an activated/effector phenotype. But interestingly, not seeing the classical exhaustion markers like PDCD1, LAG3 that are often present in tumor-infiltrating T cells. This could be a more recently activated population.  The presence of HLA molecules (HLA-A, HLA-C, HLA-E) and B2M suggests active antigen presentation interaction capacity. CXCR4 indicates these cells likely have tissue-homing capabilities.  Let me think about other possibilities... Could these be innate lymphoid cells (ILCs)? They share some markers with T cells but again, the complete CD3 complex really defines this as T cells.  Looking at metabolic signatures - LDHB suggests these cells are metabolically active, consistent with an activated T cell state.  The combination of IL2RG (CD132), CD3 complex, and TCR signaling molecules like LCK forms an extremely strong case for T cells. The presence of CD27 specifically suggests these might be memory/effector T cells.  Time to rank the possibilities based on marker specificity and pathway evidence... |
| --- |

Based on comprehensive analysis of the markers, pathways, and biological context, here are the three most likely cell types in order of probability:

1. T Cells (Highest Confidence)

- Definitive Markers Present: Complete CD3 complex (CD3D/E/G), CD2, CD7, LCK
- Supporting Pathways: TCR Signaling, T Cell Activation
- State: Activated/Effector phenotype (CD69+, IL7R+)
- Gold Standard Validation Markers: CD28, THEMIS (not in input set)

1. NK Cells (Lower Probability)

- Supporting Markers: CD2, CD7, KLRB1
- Missing Critical Markers: NCAM1 (CD56), NKG7
- State: Would be active based on signaling markers
- Gold Standard Validation Markers: FCGR3A, GNLY (not in input set)

1. Innate Lymphoid Cells (Lowest Probability)

- Supporting Markers: IL7R, KLRB1
- Missing Critical Markers: RORγt, GATA3
- State: Would be tissue-resident based on CXCR4
- Gold Standard Validation Markers: IL23R, KIT (not in input set)

The presence of the complete CD3 complex along with TCR signaling machinery makes T cells the overwhelmingly likely identity, with other lymphoid possibilities being much less probable given the marker profile.

**Score**

| Plain Text Let me carefully score each predicted cell type using the criteria with pathway since we have pathway information...  Starting with T cells: Marker Profile analysis... Strong matching markers: CD3D/E/G complex (very specific), CD2, CD7, LCK, IL7R - these are classical T cell markers, definitely worth high points. PTPRC as pan-immune marker also supports. Looking at narrow markers - CD3 complex is extremely specific for T cells, that's worth full narrow marker points. No negative markers present that would exclude T cells. TCR pathway and T cell activation pathways are perfectly aligned. In PDAC context, T cells are absolutely expected and their activated state makes perfect biological sense.  For NK cells: Marker Profile... Some matching markers like CD2, CD7, KLRB1 but these are shared with T cells (-10 points). Missing critical NK-specific narrow markers. Pathways don't specifically support NK identity - TCR signaling actually conflicts (-20). While NK cells are plausible in PDAC, the marker/pathway profile doesn't strongly support this identity.  For ILCs: Marker Profile... Very few specific matching markers - mainly just IL7R which is shared (-10). No narrow markers specific to ILCs. TCR signaling pathway actually conflicts with ILC identity (-20). While ILCs can be present in PDAC, the evidence here is very weak.  Let me calculate final scores:  T cells: Markers: 45 (matching) + 15 (narrow) = 60 Pathways: 15 (state) + 5 (type) = 20 Context: 10 (type) + 10 (state) = 20 Total: 100  NK cells: Markers: 20 (matching) + 0 (narrow) - 10 (shared) = 10 Pathways: 0 + (-20) (conflicting) = -20 Context: 10 (type) + 5 (state) = 15 Total: 5  ILCs: Markers: 10 (matching) + 0 (narrow) - 10 (shared) = 0 Pathways: (-20) (conflicting) = -20 Context: 10 (type) + 5 (state) = 15 Total: -5 |
| --- |

T Cells: 100

NK Cells: 5

Innate Lymphoid Cells: -5

**Report**

**Celltype Prediction**

**T Cells (score: 100)**

Active effector/memory T cell state

**Key Markers**:

- Cell-type-specific: CD3D, CD3E, CD3G, CD2, CD7, LCK, PTPRC
- Cell-state-specific: CD69, IL7R, CD27, IL32, CXCR4

**Evidence and Reasoning**

The presence of the complete CD3 complex (CD3D/E/G) along with TCR signaling components (LCK) provides definitive evidence for T cell identity. The enrichment of TCR signaling and T cell activation pathways strongly supports this classification. The expression of activation markers (CD69, IL7R) and memory marker CD27 suggests an active effector/memory phenotype. The presence of CXCR4 indicates tissue-homing capability, while IL32 suggests pro-inflammatory function. In PDAC context, these likely represent tumor-infiltrating T cells engaged in anti-tumor response. The co-expression of HLA molecules (HLA-A/C/E) and B2M suggests active involvement in antigen presentation interactions. The metabolic profile (LDHB) is consistent with activated T cell state.

**Validation**: CD28, THEMIS, CD5, TRAC

**NK Cells (score: 5)**

Active cytotoxic state

**Key Markers**:

- Cell-type-specific: CD2, CD7, KLRB1
- Cell-state-specific: CXCR4, IL32

**Evidence and Reasoning**

While some markers overlap with T cell signature (CD2, CD7), the presence of KLRB1 (CD161) could suggest NK cell identity. The expression of IL32 and tissue-homing receptor CXCR4 would be consistent with activated NK cells in tumor microenvironment. However, the absence of definitive NK markers and the presence of T cell-specific markers make this classification less likely. The enriched TCR signaling pathways actually conflict with NK cell identity, as NK cells utilize different signaling cascades. While NK cells are common in PDAC tumor microenvironment, the overall expression profile more strongly supports T cell identity.

**Validation**: NCAM1, FCGR3A, GNLY, NKG7

**Innate Lymphoid Cells (score: -5)**

Tissue-resident active state

**Key Markers**:

- Cell-type-specific: IL7R, KLRB1
- Cell-state-specific: CXCR4, IL32

**Evidence and Reasoning**

The expression of IL7R and KLRB1 could potentially indicate ILC identity, as these markers are shared across innate lymphoid lineages. The presence of tissue-homing receptor CXCR4 and inflammatory mediator IL32 would be consistent with tissue-resident ILCs in tumor microenvironment. However, the presence of T cell-specific markers (CD3 complex) and TCR signaling pathways directly conflicts with ILC identity, as ILCs lack TCR expression. While ILCs can be present in PDAC tissue, the molecular evidence strongly argues against this classification for the current gene signature.

**Validation**: RORC, GATA3, IL23R, KIT

**Novel Insights**

The gene signature reveals an interesting T cell state in PDAC microenvironment that combines features of activation (CD69, IL32) with memory markers (CD27) but notably lacks classical exhaustion markers often seen in tumor-infiltrating T cells. The strong expression of tissue-homing receptor CXCR4 suggests active recruitment to the tumor site. The co-expression of HLA molecules with activation markers might indicate potential involvement in antigen presentation or T cell-T cell interactions. The metabolic profile suggests these cells maintain active functionality rather than exhaustion, which could have implications for anti-tumor immunity and immunotherapy approaches in PDAC.
