# Supplementary material for: GPTBioInsightor—leveraging large language models for transparent scRAN-seq cell type annotations
Source: Bioinform Adv. 2026 Jan 22;6(1):vbag025. doi: 10.1093/bioadv/vbag025 (PMC12975716; doi:10.1093/bioadv/vbag025)
Supplement: vbag025_Supplementary_Data [file vbag025_supplementary_data.zip › Supplementary_info_1.docx]

**CellType Analysis**

**Potential CellType**

In scRNA-Seq data background of 'Cells are PBMCs from a Healthy Donor', the following Potential CellType to be identified:

| Plain Text Let me analyze all the cell types and states mentioned in previous responses... need to carefully evaluate which ones make the most biological sense in healthy PBMCs and have reliable transcriptional signatures.  Starting with T cells - definitely one of the most abundant and reliably detected. CD3 genes are classic markers. States should reflect homeostatic conditions in circulation...  B cells - CD19, CD20 (MS4A1) as canonical markers. Naive vs memory states are well-established and detectable through transcriptional programs.  NK cells - looking at NCAM1, FCGR3A... their states in healthy conditions would mainly reflect different functional programs.  For myeloid cells... CD14 and CD16 (FCGR3A) clearly separate classical vs non-classical monocytes. Need to think about which states are most relevant in steady state.  DCs - looking at markers like CLEC9A, CD1C... conventional vs plasmacytoid is probably more reliable than maturation states for classification.  Plasma cells - while they can be detected through immunoglobulin expression, they're relatively rare in healthy PBMCs. Should I include them?  MAIT cells - while they're a distinct population, might be better considered as a T cell subtype rather than a broad category.  Platelets - while they do contaminate PBMC preps and have distinct markers like PPBP, they're not really immune cells...  Let me cross-reference with typical scRNA-seq clustering results and focus on the most robust populations and states... |
| --- |

Candidate celltype:

T cells: CD3D, CD3E, CD3G

- Naive: CCR7, SELL, LEF1, high metabolic quiescence
- Memory: IL7R, ANXA1, enhanced effector response capacity

B cells: CD19, MS4A1, CD79A

- Naive: IGHD, IL7R, low antigen experience
- Memory: CD27, IGHG1, antigen-experienced

NK cells: NCAM1, FCGR3A, NKG7

- Cytotoxic: GNLY, GZMB, high cytolytic activity
- Regulatory: XCL1, CCL3, cytokine production

Classical Monocytes: CD14, LYZ, MS4A7

- Surveillance: CX3CR1, SELL, patrolling behavior
- Inflammatory: IL1B, TNF, pro-inflammatory capacity

Dendritic Cells: HLA-DRA, FCER1A, CD1C

- Conventional: CLEC9A, CD1C, antigen presentation
- Plasmacytoid: IL3RA, TCF4, type I interferon production

Non-Classical Monocytes: FCGR3A, LST1, MS4A7

- Patrolling: CX3CR1, ITGAL, vascular surveillance
- Inflammatory: S100A8, S100A9, inflammatory response

B Memory Plasma: CD27, CD38, SDC1

- Early: TNFRSF17, XBP1, initial differentiation
- Mature: IGJ, MZB1, high antibody production

Regulatory T cells: FOXP3, IL2RA, CTLA4

- Resting: SELL, CCR7, suppressive maintenance
- Activated: ICOS, TNFRSF9, enhanced regulatory function

**cluster geneset 0**

**Gene List**

Top genes

| Plain Text LDHB,CD3D,TPT1,EEF1A1,LTB,CD3E,IL32,IL7R,NPM1,JUNB,MALAT1,TMEM66,NOSIP,BTG1,AES,EEF1B2,GLTSCR2,CD2,HINT1,GIMAP7,CD27,LCK,CD7,CCR7,JUN |
| --- |

enrichment pathway

| Plain Text  - WikiPathways_2024_Human: Cytoplasmic Ribosomal Proteins ,Modulators Of TCR Signaling And T Cell Activation ,Th17 Cell Differentiation Pathway ,T Cell Receptor Signaling ,T Cell Antigen Receptor TCR Pathway During Staph A. Infection |
| --- |

**celltype thinking**

| Plain Text Hmm, looking at this PBMC dataset from a healthy donor... let me carefully analyze the molecular signatures here. The geneset immediately catches my attention with some very telling markers.  CD3D and CD3E jump out right away - these are absolutely fundamental components of the TCR complex. Combined with CD2, LCK, and CD7, this is screaming T cell lineage. The pathway enrichment strongly supports this too - TCR signaling, T cell activation... this is classic T cell biology.  Looking deeper at the state markers... IL7R is particularly interesting - it's crucial for T cell homeostasis and survival, especially in naive and memory T cells. CCR7 is also quite telling - it's a key trafficking receptor for naive and central memory T cells.  CD27 presence is intriguing... while it's often associated with memory B cells, it's also expressed on T cells, particularly during activation and memory formation. In this T cell context, it likely indicates a memory or recently activated state.  The pathway analysis reveals both basic T cell programs (TCR signaling) and more specific ones like Th17 differentiation. Though I need to be careful not to over-interpret - presence of a pathway doesn't necessarily mean these are Th17 cells.  I'm also seeing lots of housekeeping and metabolic genes - LDHB, EEF1A1, TPT1... This suggests these cells are transcriptionally active but not necessarily in an highly activated state.  Let me think about alternative possibilities... Could these be NK cells? While NK cells share some markers with T cells, the strong TCR component rules that out. B cells? No, the absence of B cell-specific markers like CD19/CD79A and the presence of clear T cell signatures makes that unlikely.  Looking at the states within T cells... The combination of CCR7, IL7R, and CD27 is particularly informative. This pattern is most consistent with either naive T cells or central memory T cells. The presence of immediate early genes like JUN and JUNB suggests these cells might be in a poised or recently activated state, but not fully effector.  Let me cross-reference with the candidate cell types... Yes, this fits well with several T cell states, but I need to be precise about which ones are most likely based on this exact marker combination... |
| --- |

Based on comprehensive analysis of markers, pathways, and biological context, the three most likely cell types/states are:

1. Naive T cells

- Key supporting markers: CD3D/E (TCR complex), CCR7 (trafficking), IL7R (homeostasis)
- Supporting pathways: TCR signaling, T cell activation
- Biological context: Common in healthy PBMCs
- Validation markers: SELL, LEF1 (not in cluster markers)

1. Central Memory T cells

- Key supporting markers: CD3D/E, CCR7, IL7R, CD27
- Supporting pathways: TCR signaling, enhanced metabolic activity
- Biological context: Natural presence in circulating PBMCs
- Validation markers: ANXA1, BCL2 (not in cluster markers)

1. Early Activated T cells

- Key supporting markers: CD3D/E, JUNB, JUN (immediate early genes)
- Supporting pathways: TCR signaling, T cell activation
- Biological context: Small population in healthy PBMCs
- Validation markers: CD69, FOS (not in cluster markers)

**Score**

| Plain Text Alright, let me carefully score each predicted cell type using the criteria with pathway since we have pathway information. Need to be extremely thorough and objective here...  Starting with Naive T cells: Marker Profile analysis... CD3D/E are classical T cell markers - strong positive. CCR7 and IL7R are characteristic of naive state. Looking at narrow markers - CD3D/E/LCK combination is quite specific. These markers are shared with some other T cell states though, need to subtract points there. No negative markers present.  Pathway check for naive T cells... TCR signaling matches cell type perfectly. T cell activation pathways are present but not overly active, consistent with naive state. Some pathway overlap with other T cell states, but that's expected.  Biological context - naive T cells are absolutely expected in healthy PBMCs, both type and state are highly plausible.  Moving to Central Memory T cells: Markers... CD3D/E again strong positive. CD27 and IL7R support memory phenotype. The combination is less specific than for naive T cells though. No negative markers.  Pathways... TCR signaling fits, but the enhanced metabolic signature isn't strongly evident. Some pathway sharing with other T states.  Context is perfectly plausible for healthy PBMCs.  Early Activated T cells: Marker analysis... CD3D/E present, JUNB/JUN indicate activation, but the signature isn't as complete as we'd expect for activated T cells. No negative markers though.  Pathways... T cell activation pathways present but the overall profile isn't strongly skewed toward activation.  Context - while possible to find some activated T cells in healthy PBMCs, it's less typical than naive or memory cells.  Let me calculate final scores based on exact criteria... |
| --- |

Naive T cells: 92

Central Memory T cells: 85

Early Activated T cells: 75

**Report**

**Celltype Prediction**

**Celltype1: Naive T cells (score: 92)**

Resting/homeostatic state with potential for activation

**Key Markers**:

- Cell-type-specific: CD3D, CD3E, LCK, CD2, CD7
- Cell-state-specific: CCR7, IL7R, NOSIP, BTG1

**Evidence and Reasoning**

The presence of multiple core T cell receptor components (CD3D, CD3E) alongside TCR signaling molecules (LCK, CD2) provides strong evidence for T cell identity. The expression of CCR7 and IL7R is characteristic of naive T cells, as these markers are crucial for lymph node homing and homeostatic survival respectively. The enrichment of TCR signaling pathways and T cell activation modules, combined with the expression of regulatory factors like BTG1 and NOSIP, suggests these cells are in a homeostatic state while maintaining response readiness. This molecular profile is highly consistent with naive T cells in circulation, which require constant survival signals while maintaining a quiescent phenotype until antigen encounter. The presence of housekeeping genes (LDHB, EEF1A1) further supports their basal metabolic state.

**Validation**: SELL, LEF1, TCF7

**Celltype2: Central Memory T cells (score: 85)**

Resting memory state

**Key Markers**:

- Cell-type-specific: CD3D, CD3E, LCK, CD2
- Cell-state-specific: CD27, IL7R, CCR7, JUNB

**Evidence and Reasoning**

The expression profile combines core T cell markers (CD3D/E, LCK) with memory-associated molecules, particularly CD27 and IL7R. The presence of CCR7 indicates central memory rather than effector memory phenotype, as these cells retain lymph node homing capacity. The enrichment of TCR signaling pathways alongside the expression of immediate early genes like JUNB suggests these cells maintain a primed state characteristic of memory T cells. This molecular signature is consistent with central memory T cells in healthy circulation, which must balance long-term survival with rapid response capability. The moderate expression of housekeeping and metabolic genes indicates a slightly elevated but controlled baseline activity compared to naive cells.

**Validation**: ANXA1, BCL2, TNFRSF7

**Celltype3: Early Activated T cells (score: 75)**

Initial activation state

**Key Markers**:

- Cell-type-specific: CD3D, CD3E, LCK
- Cell-state-specific: JUNB, JUN, CD27

**Evidence and Reasoning**

The molecular signature combines core T cell markers with early activation indicators, particularly the immediate early genes JUNB and JUN. The enrichment of T cell activation pathways and presence of CD27 suggests these cells are transitioning from a resting to an activated state. While the full activation signature isn't present, the combination of TCR signaling components with early response genes indicates recent or ongoing activation. This profile is consistent with T cells beginning to respond to stimuli, though the absence of classical activation markers suggests this is an early stage. The presence in healthy PBMCs is plausible but less common, representing cells responding to homeostatic signals or recent antigenic encounters.

**Validation**: CD69, FOS, ZFP36

**Novel Insights**

The molecular profile reveals an interesting gradient of T cell states within this population, suggesting a continuous spectrum rather than discrete states. The presence of immediate early genes alongside homeostatic markers indicates these cells maintain a poised state that enables rapid response while preventing inappropriate activation. The enrichment of both TCR signaling and metabolic pathways suggests a sophisticated regulatory network that balances readiness with quiescence. This may represent a previously underappreciated steady-state characteristic of circulating T cells, where cells continuously tune their activation threshold based on homeostatic signals while maintaining lineage identity through stable expression of core T cell markers.

**cluster geneset 1**

**Gene List**

Top genes

| Plain Text CD74,CD79A,HLA-DRA,CD79B,HLA-DPB1,MS4A1,HLA-DQA1,CD37,HLA-DRB1,HLA-DQB1,HLA-DPA1,HLA-DRB5,TCL1A,LINC00926,HLA-DMA,VPREB3,HLA-DQA2,HLA-DMB,LTB,BANK1,HVCN1,CXCR4,LAPTM5,ISG20,FCER2 |
| --- |

enrichment pathway

| Plain Text  - WikiPathways_2024_Human: Cytoplasmic Ribosomal Proteins ,Allograft Rejection ,B Cell Receptor Signaling ,Extrafollicular And Follicular B Cell Activation By SARS CoV 2 ,Ebola Virus Infection In Host |
| --- |

**celltype thinking**

| Plain Text Hmm, looking at this gene signature from PBMCs... let me dive deep into what these genes are telling us about cell identity and state...  The most striking thing is the heavy enrichment of HLA class II genes - HLA-DRA, DRB1, DPA1, DPB1, DQA1, DQB1... This immediately points to professional antigen presenting cells. But which type?  CD79A and CD79B jump out at me - these form the B cell receptor complex. Along with MS4A1 (CD20) and BANK1, this strongly suggests B cells. TCL1A is particularly interesting - it's highly expressed in naive B cells and some memory B cells...  The pathway enrichment really supports this - B Cell Receptor Signaling is a major hit. That makes perfect biological sense with the marker profile.  CXCR4 expression is intriguing - while not B cell-specific, it's important for B cell trafficking and germinal center organization. Combined with FCER2 (CD23), this suggests we're looking at mature B cells.  Could this be dendritic cells? They also express high levels of HLA class II... but the strong BCR components (CD79A/B) and B cell-specific factors like MS4A1 and BANK1 really argue against that. The complete signature is much more B cell-like.  What about plasma cells? The lack of high immunoglobulin genes and typical plasma cell markers like SDC1 (CD138) suggests these aren't terminally differentiated antibody-secreting cells.  Looking at the broader context - these are healthy donor PBMCs, where we'd expect to find circulating B cells in various differentiation states. The signature suggests these are mature B cells, likely a mix of naive and memory based on TCL1A expression.  Let me think about validation markers we could use... CD19 would be perfect since it's not in this gene list but is a canonical B cell marker. CD20 surface staining would also validate the MS4A1 transcript data.  The pathway enrichment adds another layer - allograft rejection and B cell activation pathways fit perfectly with B cell biology. The ribosomal signature suggests these are transcriptionally active cells.  I should also consider the relative abundance we'd expect in PBMCs - B cells typically make up about 5-15% of PBMCs, which would make them a major population that could form a distinct cluster. |
| --- |

Based on comprehensive analysis of the markers, pathways, and biological context, here are the three most likely cell type possibilities ranked by confidence:

1. Mature B Cells (Highest Confidence)

- Key Evidence: CD79A/B (BCR complex), MS4A1 (CD20), HLA class II genes
- Supporting Factors: B cell-specific pathways, BANK1, TCL1A expression
- Validation Markers: CD19, CD22, CD20 surface protein
- Biological Context: Expected in healthy PBMCs

1. Memory B Cells (Moderate Confidence)

- Key Evidence: High HLA class II expression, CXCR4, mature B cell markers
- Supporting Factors: BCR signaling pathway, antigen presentation capacity
- Validation Markers: CD27, CD95
- Biological Context: Common in circulating PBMCs

1. Naive B Cells (Moderate Confidence)

- Key Evidence: TCL1A expression, FCER2 (CD23)
- Supporting Factors: B cell activation pathways
- Validation Markers: IgD, CD38
- Biological Context: Major B cell population in healthy blood

**Score**

| Plain Text Let me carefully evaluate each predicted cell type using the scoring criteria with pathways since we have pathway data...  Starting with Mature B Cells: Marker Profile... CD79A/B are highly specific B cell markers - that's strong narrow category evidence. MS4A1 (CD20) is another classic B cell marker. HLA class II genes are present but they're shared with other APCs, so need to deduct some points there. No negative markers that would rule out B cells...  Looking at about 40 for matching markers, 15 for narrow markers, -10 for shared markers = 45 pts  Pathway analysis... B Cell Receptor Signaling pathway is a perfect match for cell type. Allograft rejection and B cell activation pathways align with B cell state. Some overlap with other immune cells but no truly conflicting pathways. That's 5 for cell type match, 15 for state match, -10 for shared = 10 pts  Context is perfect - B cells are expected in healthy PBMCs in this state = 20 pts Total: 75 points  For Memory B Cells: Similar marker profile but TCL1A actually argues somewhat against pure memory phenotype... About 35 for matching, 10 for narrow, -10 for shared = 35 pts Pathways still fit but less specifically = 10 pts Context is appropriate = 20 pts Total: 65 points  For Naive B Cells: TCL1A and FCER2 support this, along with core B cell markers... About 40 for matching, 15 for narrow, -10 for shared = 45 pts Pathways align well = 10 pts Context is appropriate = 20 pts Total: 75 points  Interesting that Naive B cells scored equally with mature B cells - the markers really could represent either or a mix of both states. Memory B cells scored slightly lower due to some markers being less consistent with a pure memory phenotype. |
| --- |

Mature B Cells: 75

Memory B Cells: 65

Naive B Cells: 75

**Report**

**Celltype Prediction**

**Mature B Cells (score: 75)**

Cell state: Transcriptionally active, antigen-presentation competent

**Key Markers**:

- Cell-type-specific: CD79A, CD79B, MS4A1, BANK1
- Cell-state-specific: HLA-DRA, HLA-DRB1, HLA-DPA1, HLA-DPB1, CXCR4, FCER2

**Evidence and Reasoning**

The presence of B cell receptor complex components (CD79A/B) along with MS4A1 (CD20) provides strong evidence for B cell identity. High expression of multiple HLA class II genes indicates active antigen presentation capacity. The enrichment of B Cell Receptor Signaling pathway strongly supports B cell identity, while allograft rejection and B cell activation pathways suggest an immunologically active state. CXCR4 expression indicates normal B cell trafficking capability. The cellular context of healthy PBMCs is entirely consistent with mature B cells, which typically comprise 5-15% of peripheral blood mononuclear cells. The ribosomal protein pathway enrichment suggests these cells are transcriptionally active, consistent with mature B cell function.

**Validation**: CD19, CD20 surface protein, CD22

**Naive B Cells (score: 75)**

Cell state: Antigen-inexperienced, homeostatic

**Key Markers**:

- Cell-type-specific: CD79A, CD79B, MS4A1, TCL1A
- Cell-state-specific: FCER2, CXCR4, HLA class II genes

**Evidence and Reasoning**

The expression of TCL1A and FCER2 (CD23) strongly suggests a naive B cell phenotype, as these markers are characteristically expressed in antigen-inexperienced B cells. The presence of core B cell markers (CD79A/B, MS4A1) confirms B cell identity. The B cell activation pathway enrichment, combined with high HLA class II expression, indicates these cells are functionally competent yet not terminally differentiated. The absence of typical memory or plasma cell markers supports a naive phenotype. In healthy PBMCs, naive B cells represent a significant proportion of the B cell pool, making this prediction biologically plausible. The ribosomal pathway enrichment suggests these cells maintain homeostatic protein synthesis.

**Validation**: IgD, CD38low, IL7R

**Memory B Cells (score: 65)**

Cell state: Antigen-experienced, resting

**Key Markers**:

- Cell-type-specific: CD79A, CD79B, MS4A1
- Cell-state-specific: HLA class II genes, CXCR4

**Evidence and Reasoning**

While sharing core B cell markers (CD79A/B, MS4A1) with other B cell subtypes, the high expression of HLA class II genes and CXCR4 is consistent with memory B cell phenotype. The enrichment of B cell receptor signaling and activation pathways supports an antigen-experienced state. However, the presence of TCL1A, which is typically lower in memory cells, makes this prediction less certain than the other two possibilities. The context of healthy PBMCs is appropriate for memory B cells, which circulate as part of immunological memory. The combination of markers suggests these cells maintain antigen presentation capability while in a resting state.

**Validation**: CD27, CD95, switched immunoglobulin isotypes

**Novel Insights**

The gene expression profile reveals an interesting pattern of concurrent high HLA class II expression with B cell-specific markers, suggesting these cells may be particularly active in antigen presentation. The presence of CXCR4 and FCER2 along with TCL1A points to a possible transitional state or subset of B cells that maintains characteristics of both naive and more differentiated states. The enrichment of ribosomal proteins pathway alongside B cell activation pathways suggests these cells are maintaining active protein synthesis machinery while in a homeostatic state, potentially primed for rapid response to antigenic stimulation. This could represent a previously under-appreciated state of B cell activation readiness in healthy circulation.

**cluster geneset 2**

**Gene List**

Top genes

| Plain Text LST1,FCER1G,AIF1,COTL1,FCGR3A,FTH1,IFITM3,SAT1,IFITM2,PSAP,FTL,SERPINA1,CTSS,S100A11,OAZ1,CD68,S100A4,CST3,TIMP1,RP11-290F20.3,CFD,CEBPB,SPI1,ACTB,TYROBP |
| --- |

enrichment pathway

| Plain Text  - WikiPathways_2024_Human: Electron Transport Chain OXPHOS System In Mitochondria ,Nonalcoholic Fatty Liver Disease ,TYROBP Causal Network In Microglia ,VEGFA VEGFR2 Signaling ,Proteasome Degradation |
| --- |

**celltype thinking**

| Plain Text Hmm, looking at this gene signature from PBMCs... let me trace through the markers and pathways carefully. The context of healthy donor PBMCs is crucial here.  FCGR3A (CD16) immediately catches my attention - this is a classic marker for NK cells and non-classical monocytes. But I need to look at the whole signature pattern...  The presence of FCER1G, AIF1, CD68, CTSS is strongly suggestive of myeloid lineage. And SPI1 (PU.1) - that's a master regulator of myeloid development. CEBPB too points toward myeloid identity.  TYROBP (DAP12) is particularly interesting - it's enriched in both NK cells and myeloid cells, and I see the TYROBP pathway is enriched. This adaptor protein mediates activating signals in both lineages.  The presence of S100A4, S100A11 makes me think of inflammatory/activated states. Along with TIMP1 and the VEGFA-VEGFR2 pathway enrichment... suggests these cells might be involved in tissue remodeling or inflammatory responses.  Looking at lysosomes/phagocytic markers - CTSS, CD68, PSAP... this really strengthens the myeloid hypothesis. The electron transport chain pathway enrichment also fits with metabolically active myeloid cells.  Could these be classical monocytes? No, the absence of CD14 and strong FCGR3A expression argues against that.   Non-classical monocytes are looking very likely - LST1 is a key marker in the input that matches perfectly with the candidate list. The overall signature with FCGR3A, CD68, and myeloid transcription factors fits beautifully.  NK cells remain a possibility given FCGR3A and TYROBP, but the strong myeloid programming and lack of cytotoxic molecules like GNLY or granzymes makes this less likely as the primary identity.  DCs? The FCER1A connection is interesting, but the overall signature doesn't strongly support this.  Let me cross-reference with known transcriptional programs... yes, this signature most strongly aligns with:  1. Non-classical monocytes (highest confidence) - the LST1/FCGR3A/myeloid signature is definitive 2. Classical monocytes (possible but less likely) - missing key markers like CD14 3. NK cells (possible minority population) - some overlapping markers but lacking canonical NK programs  For validation, I'd look for: - CD16 protein (FCGR3A) - should be high - CX3CR1 - should be expressed for non-classical phenotype - CD14 - should be low/negative to distinguish from classical monocytes |
| --- |

Based on comprehensive analysis of markers, pathways and biological context in healthy PBMCs, the three most likely cell types are:

1. Non-Classical Monocytes (Highest Confidence)

- Key markers present: LST1, FCGR3A, CD68, SPI1, CEBPB
- Supporting pathways: TYROBP signaling, metabolic activation
- Consistent with patrolling/surveillance function
- Gold standard validation markers: CX3CR1, CD16 (protein)

1. Classical Monocytes (Lower Confidence)

- Supporting markers: CD68, CTSS, myeloid transcription factors
- Missing key marker CD14 makes this less likely
- Gold standard validation marker: CD14 (protein)

1. NK Cells (Possible Minor Population)

- Partial marker overlap: FCGR3A, TYROBP
- Missing canonical NK programs
- Gold standard validation markers: NKG7, GNLY, KLRD1

**Score**

| Plain Text Let me carefully evaluate each predicted cell type using the scoring criteria with pathways since we have pathway information...  Starting with Non-Classical Monocytes: Marker Profile: - Strong matching markers: LST1, FCGR3A, CD68, SPI1, CEBPB (~40/45 pts) - Narrow markers present: LST1 specifically (~12/15 pts) - Some marker overlap with NK cells (-10 pts) - No negative markers present (0 pts)  Pathway Profile: - TYROBP pathway perfectly matches cell type (5 pts) - Metabolic pathways match activated state (12/15 pts) - Some pathway overlap with other immune cells (-5 pts)  Biological Context: - Completely plausible in healthy PBMCs (10 pts) - Active surveillance state fits context (10 pts) Total: ~74 points  For Classical Monocytes: Marker Profile: - Some matching markers: CD68, CTSS (~25/45 pts) - Limited narrow markers (~5/15 pts) - Significant marker overlap (-10 pts) - Missing CD14 isn't counted against per reminder  Pathway Profile: - Pathways partially match (10/15 pts) - Significant overlap (-10 pts)  Context: - Plausible in PBMCs (20/20 pts) Total: ~40 points  For NK Cells: Marker Profile: - Limited matching markers: FCGR3A, TYROBP (~20/45 pts) - Very few narrow markers (~5/15 pts) - Significant overlap with myeloid markers (-10 pts) - Missing cytotoxic program isn't counted against  Pathway Profile: - TYROBP pathway matches (5 pts) - Limited state-specific pathway matches (5/15 pts) - Shared pathways (-10 pts)  Context: - Plausible in PBMCs (20/20 pts) Total: ~35 points |
| --- |

Non-Classical Monocytes: 74

Classical Monocytes: 40

NK Cells: 35

**Report**

**Celltype Prediction**

**Non-Classical Monocytes (score: 74)**

Active patrolling/surveillance state

**Key Markers**:

- Cell-type-specific: LST1, FCGR3A, CD68, SPI1, CEBPB
- Cell-state-specific: S100A11, S100A4, TIMP1, IFITM2/3

**Evidence and Reasoning**

The expression profile strongly indicates non-classical monocytes with key lineage-defining markers LST1 and FCGR3A, supported by myeloid transcription factors SPI1 and CEBPB. The presence of TYROBP signaling pathway and phagocytic markers (CD68, CTSS) confirms myeloid identity. The cell state markers (S100A11, S100A4) and VEGFA-VEGFR2 pathway suggest active surveillance function. The enrichment of metabolic pathways (electron transport chain, OXPHOS) aligns with the energetic demands of patrolling behavior. This molecular signature, combined with the healthy PBMC origin, strongly supports an activated non-classical monocyte population engaged in vascular surveillance.

**Validation**: CX3CR1, CD16 (protein), ITGAL

**Classical Monocytes (score: 40)**

Inflammatory-poised state

**Key Markers**:

- Cell-type-specific: CD68, CTSS, SPI1, CEBPB
- Cell-state-specific: TIMP1, S100A11, IFITM2/3

**Evidence and Reasoning**

The presence of canonical myeloid markers (CD68, CTSS) and master regulators (SPI1, CEBPB) suggests classical monocyte identity. The inflammatory state is indicated by S100A11 and TIMP1 expression, supported by metabolic pathway activation (OXPHOS, electron transport chain). While the overall signature shows myeloid characteristics, the absence of CD14 in the gene set makes this prediction less confident. The TYROBP pathway enrichment and phagocytic program are consistent with monocyte function, though these features are shared with other myeloid populations. The context of healthy PBMCs supports the presence of classical monocytes, albeit with lower confidence than non-classical monocytes.

**Validation**: CD14, CCR2, CD163

**NK Cells (score: 35)**

Activated surveillance state

**Key Markers**:

- Cell-type-specific: FCGR3A, TYROBP
- Cell-state-specific: IFITM2/3, S100A11

**Evidence and Reasoning**

The NK cell identification is supported by the presence of FCGR3A and TYROBP, which are important components of NK cell signaling. The enriched TYROBP Causal Network pathway further supports this identity. However, the absence of canonical NK cell programs and cytotoxic molecules makes this prediction less certain. The activation state is suggested by IFITM2/3 expression and metabolic pathway enrichment, indicating altered cellular energetics. While NK cells are expected in healthy PBMCs, the limited NK-specific markers and strong myeloid signature suggest this may represent a minor population or cells sharing common features with myeloid lineages.

**Validation**: NKG7, GNLY, KLRD1, NCR1

**Novel Insights**

The gene signature reveals an interesting intersection of immune surveillance and metabolic activation in circulating immune cells. The strong enrichment of both TYROBP signaling and metabolic pathways (OXPHOS, electron transport chain) suggests a previously underappreciated link between immune cell patrolling functions and energy metabolism. The presence of tissue remodeling factors (TIMP1) and stress response proteins (S100A11, S100A4) in non-classical monocytes indicates these cells maintain tissue homeostasis even in healthy conditions. This molecular profile provides new insights into the baseline activation state of circulating immune cells and their potential role in steady-state immune surveillance.

**cluster geneset 3**

**Gene List**

Top genes

| Plain Text NKG7,GZMB,GNLY,CTSW,PRF1,CST7,GZMA,HLA-C,FGFBP2,B2M,HLA-A,CD247,FCGR3A,SPON2,HLA-B,GZMM,PLAC8,CCL4,CD7,UBB,SRGN,ITGB2,HOPX,ID2,PFN1 |
| --- |

enrichment pathway

| Plain Text  - WikiPathways_2024_Human: Proteasome Degradation ,Interactions Of Natural Killer Cells In Pancreatic Cancer ,TAR Syndrome ,VEGFA VEGFR2 Signaling ,Ebola Virus Infection In Host |
| --- |

**celltype thinking**

| Plain Text Hmm, looking at this geneset from cluster 3... very interesting expression profile. Let me trace through the markers and pathways carefully...  NKG7, GZMB, GNLY, PRF1 - this is screaming cytotoxic program! These are the core cytolytic machinery genes. But which cytotoxic cell type? Need to look deeper...  FCGR3A (CD16) is present - this immediately makes me think of either NK cells or CD16+ monocytes. But wait, looking at the full context - all these cytotoxic genes together with FCGR3A strongly points to NK cells rather than monocytes.  The presence of CD7 is also quite telling - it's expressed on NK cells and T cells, but not monocytes. And ID2 - that's a key transcription factor for NK cell development.  HLA-A, -B, -C and B2M expression - while these are broadly expressed, NK cells do express MHC-I molecules. This fits with the NK cell hypothesis.  Looking at the pathways... "Interactions of Natural Killer Cells" pathway enrichment provides additional support. The proteasome pathway makes sense too - NK cells need protein degradation machinery for their cytotoxic functions.  Could these be cytotoxic T cells though? Let me check... while they share many of these cytotoxic genes, I don't see clear T cell-specific markers like CD3 components (except CD247/CD3ζ which is also in NK cells). No clear TCR genes either.  The combination of FCGR3A with this cytotoxic program, plus CD7 and ID2, really points to NK cells rather than CTLs.  Looking at the NK cell states in our candidate list... this matches perfectly with the cytotoxic NK profile - GNLY, GZMB are literally the defining features there.  For validation, I should suggest looking for other NK markers not in this set - NCAM1 (CD56), KLRD1 (CD94) would be good gold standards.  Let me also consider any other possibilities... The cytotoxic program could technically be from other cytotoxic lymphocytes like γδ T cells or MAIT cells. But without their specific markers, and with such clear NK cell features, those seem less likely.  The presence of CCL4 and the cytotoxic program could also suggest activated CD8+ T cells, but again, the lack of T cell-specific markers and presence of NK markers makes this less likely.  This really looks like a population of cytotoxic NK cells, with the gene signature and pathway enrichment providing strong, coherent evidence. |
| --- |

Based on comprehensive analysis of the markers, pathways, and biological context, here are the three most likely cell type candidates, ranked by probability:

1. Natural Killer (NK) Cells - Cytotoxic State

- Primary Evidence:
- Core cytotoxic program (NKG7, GZMB, GNLY, PRF1, GZMA)
- NK-associated markers (FCGR3A, CD7, ID2)
- NK cell pathway enrichment
- Validation Markers: NCAM1 (CD56), KLRD1 (CD94)

1. Cytotoxic T Lymphocytes (CTLs)

- Supporting Evidence:
- Cytotoxic machinery genes
- CD247 (CD3ζ) presence
- Proteasome pathway activity
- Less Likely Due To:
- Absence of T cell-specific markers (CD3D/E/G)
- Validation Markers: CD3E, CD8A

1. γδ T Cells

- Supporting Evidence:
- Cytotoxic program
- Lymphocyte markers
- Less Likely Due To:
- No γδ TCR components
- NK-specific markers present
- Validation Markers: TRGC1/2, TRDC

**Score**

| Plain Text Let me carefully score each predicted cell type using the criteria with pathway since we have pathway information...  Starting with NK Cells: Marker Profile analysis... NKG7, GZMB, GNLY, PRF1 - these are classical NK cell markers, very strong evidence FCGR3A (CD16) - key NK cell marker CD7, ID2 - additional NK-associated markers Looking at marker overlap... yes, some cytotoxic markers are shared with CTLs, need to deduct points No negative markers present, that's good  Pathway analysis for NK... "Interactions of Natural Killer Cells" pathway - perfect match for cell type Proteasome pathway fits with cytotoxic state VEGFA pathway is shared with other cell types, slight deduction needed No truly conflicting pathways  Context analysis... NK cells are definitely expected in healthy PBMCs Cytotoxic state is normal for circulating NK cells  Moving to CTLs: Marker Profile... Cytotoxic markers present but shared with NK cells CD247 present but also in NK cells Missing key T cell markers, but can't penalize for missing markers No negative markers present  Pathway analysis... Proteasome pathway fits NK cell pathway is somewhat conflicting Other pathways are non-specific  Context is fine - CTLs are normal in PBMCs  Finally γδ T cells: Marker profile is weaker Shared cytotoxic markers No specific γδ T markers No negative markers  Pathways don't specifically support γδ T identity Context is plausible but less supported by evidence  Let me calculate final scores...  NK Cells: Markers: 40 (strong specific markers) - 10 (shared markers) = 30 Pathways: 15 (state) + 5 (type) - 10 (shared) = 10 Context: 10 + 10 = 20 Total: 60  CTLs: Markers: 25 (some matching) - 10 (shared) = 15 Pathways: 5 - 20 (conflicting) = -15 Context: 20 Total: 20  γδ T: Markers: 15 - 10 (shared) = 5 Pathways: -10 (shared/conflicting) Context: 20 Total: 15 |
| --- |

NK Cells: 60

CTLs: 20

γδ T Cells: 15

**Report**

**Celltype Prediction**

**Celltype1: Natural Killer Cells (score: 60)**

Cytotoxic NK cell state

**Key Markers**:

- Cell-type-specific: FCGR3A, CD7, ID2, NKG7
- Cell-state-specific: GZMB, GNLY, PRF1, GZMA, CST7, CCL4

**Evidence and Reasoning**

The expression profile strongly indicates cytotoxic NK cells based on multiple lines of evidence. The presence of NK cell-specific markers (FCGR3A, CD7, ID2) combined with a complete cytotoxic program (GZMB, GNLY, PRF1, GZMA) provides robust identification. This is further supported by the enrichment of NK cell interaction pathways and proteasome degradation pathway, which is essential for NK cell cytotoxic functions. The expression of HLA class I genes (HLA-A, -B, -C) and B2M is consistent with NK cell biology. The presence of CCL4 suggests active cytokine production, typical of functional NK cells. This cell type and state assignment is highly plausible in healthy PBMC context, where NK cells typically comprise 5-15% of lymphocytes and maintain cytotoxic capacity as part of immune surveillance.

**Validation**: NCAM1 (CD56), KLRD1 (CD94), NCR1 (NKp46)

**Celltype2: Cytotoxic T Lymphocytes (score: 20)**

Activated cytotoxic state

**Key Markers**:

- Cell-type-specific: CD247
- Cell-state-specific: GZMB, GNLY, PRF1, GZMA, CCL4

**Evidence and Reasoning**

The presence of CD247 (CD3ζ chain) and the complete cytotoxic machinery suggests CTLs as a possibility, though with lower confidence than NK cells. The proteasome degradation pathway enrichment supports active cytotoxic functions, consistent with CTL biology. The expression of HLA class I molecules and B2M aligns with CTL characteristics, and the presence of CCL4 indicates potential inflammatory activation. However, the absence of definitive T cell markers and the presence of NK-specific markers makes this a less likely assignment. The biological context of healthy PBMCs would typically include CTLs, and this activated state could represent normal immunosurveillance, but the overall evidence more strongly supports an NK cell identity.

**Validation**: CD3E, CD8A, CD3D

**Celltype3: γδ T Cells (score: 15)**

Activated cytotoxic state

**Key Markers**:

- Cell-type-specific: CD247
- Cell-state-specific: GZMB, GNLY, PRF1, GZMA

**Evidence and Reasoning**

The cytotoxic program and CD247 expression could be consistent with γδ T cells, which share many functional characteristics with NK cells and CTLs. The proteasome pathway enrichment supports cytotoxic capacity, which γδ T cells can exhibit. The presence of CCL4 and cytotoxic molecules aligns with activated γδ T cell functions. However, the lack of specific γδ T cell markers and the presence of NK cell-specific markers makes this the least likely interpretation. While γδ T cells are present in healthy PBMCs and can display cytotoxic functions, the overall expression profile more strongly supports alternative lymphocyte identities, particularly NK cells.

**Validation**: TRGC1, TRGC2, TRDC

**Novel Insights**

The gene expression profile reveals a highly active cytotoxic program with simultaneous expression of multiple granzymes, perforin, and cytokine production, suggesting a poised effector state. The co-expression of ID2 with the cytotoxic program may indicate active maintenance of cellular identity concurrent with effector functions. The presence of multiple HLA class I genes alongside cytotoxic molecules suggests sophisticated immune surveillance capabilities. The enrichment of both NK cell interaction and proteasome degradation pathways points to a mechanistic link between cellular cytotoxicity and protein degradation machinery, potentially highlighting new aspects of cytotoxic lymphocyte biology.

**cluster geneset 4**

**Gene List**

Top genes

| Plain Text CCL5,NKG7,CST7,B2M,GZMA,CTSW,HLA-C,IL32,HLA-A,GZMK,CD3D,PTPRCAP,HCST,LYAR,GZMM,CD8A,HLA-B,KLRG1,MALAT1,PRF1,CD99,GZMH,LCK,RARRES3,MYL12A |
| --- |

enrichment pathway

| Plain Text  - WikiPathways_2024_Human: Pathogenesis Of SARS CoV 2 Mediated By Nsp9 Nsp10 Complex ,Cancer Immunotherapy By PD 1 Blockade ,T Cell Receptor Signaling ,Modulators Of TCR Signaling And T Cell Activation ,TAR Syndrome |
| --- |

**celltype thinking**

| Plain Text Hmm, looking at this gene signature from cluster 4... very interesting pattern emerging here. Let me dive deep into the molecular profile...  First striking observation - strong cytotoxic program with GZMA, GZMK, GZMH, GZMM, PRF1. This immediately points me towards cytotoxic lymphocytes. The presence of these granzymes and perforin is a clear hallmark of cells with cytolytic capacity.  CD3D and LCK jump out as classic T cell markers... TCR signaling pathway enrichment too. This is starting to paint a picture of T cell identity. But wait, need to carefully consider NK cells too since they share many cytotoxic molecules.  Looking at the full profile - CD8A is present, strongly suggesting CD8+ T cells. The combination with CD3D really reinforces T cell identity over NK cells. Though NK cells could still be in the mix given NKG7 and KLRG1...  The HLA molecules (HLA-A, B, C) are interesting... while broadly expressed, their strong presence along with B2M fits with the antigen presentation machinery you'd expect in T cells.  CST7 and CTSW - these are characteristic of cytotoxic lymphocytes... commonly seen in both activated CD8 T cells and NK cells.  IL32 is intriguing - often associated with activated T cells and inflammatory responses. PTPRCAP and HCST point towards lymphocyte signaling.  Let me think about cell states... CCL5 suggests these cells might be effector or memory cells rather than naive. The strong cytotoxic program and KLRG1 really hints at an effector/effector memory phenotype.  The pathway analysis is quite telling - TCR signaling and T cell activation pathways strongly enriched. This really helps confirm we're looking at T cells rather than NK cells as the dominant population.  Checking against known subtypes... this profile aligns well with cytotoxic CD8+ T cells, particularly effector or effector memory states. The markers are too cytotoxic for naive cells, and we're missing key Treg markers like FOXP3.  Need to be thorough though - could there be other candidates? The cytotoxic profile could fit NK cells, but the strong T cell markers make them a secondary possibility. γδ T cells could potentially fit some of these markers, but the conventional CD8 profile seems more likely.  Let me validate against the Candidate_celltype list... interesting, this profile best matches T cells but with a distinct cytotoxic state that needs to be specifically called out... |
| --- |

Based on comprehensive analysis of the molecular profile and biological context, here are the three most likely cell types/states:

1. Cytotoxic CD8+ T Cells (Primary candidate)

- Core evidence: CD3D, CD8A, LCK (T cell identity)
- Functional state: GZMA, GZMK, GZMH, GZMM, PRF1 (cytotoxic program)
- Validation markers: CD8B, CD3E, CD3G

1. Effector/Memory NK Cells (Secondary candidate)

- Core evidence: NKG7, KLRG1, cytotoxic molecules
- Functional profile: CST7, CTSW, PRF1
- Validation markers: NCAM1, FCGR3A, KLRD1

1. Effector Memory CD8+ T Cells (Alternative state of primary candidate)

- Core evidence: CCL5, KLRG1 (memory/effector phenotype)
- Functional state: Cytotoxic program + IL32
- Validation markers: EOMES, PRDM1, TBX21

**Score**

| Plain Text Alright, let me carefully score each predicted cell type using the pathway-based criteria since we have pathway information...  Starting with Cytotoxic CD8+ T Cells: Marker Profile analysis... CD3D and CD8A are highly specific T cell markers - strong points here LCK is T cell specific signaling molecule Cytotoxic molecules (GZMs, PRF1) are shared with NK cells, need to deduct some points No negative markers present though  Pathway Profile: TCR signaling and T cell activation pathways are perfectly aligned PD-1 blockade pathway fits with T cell biology No major conflicting pathways  Biological Context: CD8+ T cells are absolutely expected in healthy PBMCs Cytotoxic state is physiologically normal  Moving to NK cells: Marker Profile: NKG7 and KLRG1 are present but shared with T cells Missing some key NK markers like NCAM1 Cytotoxic program fits but shared No negative markers  Pathway Profile: TCR pathways actually conflict with NK identity Some immune pathways fit generally  Context is fine for NK cells in PBMCs  For Effector Memory CD8+ T cells: Marker Profile: Shares all markers with cytotoxic CD8+ category CCL5 and KLRG1 support memory state No negative markers  Pathway Profile: TCR and activation pathways perfectly align Memory state consistent with pathways  Context: Memory T cells expected in healthy PBMCs State is physiologically appropriate  Let me calculate final scores... Cytotoxic CD8+ T:  45 (markers) - 10 (shared) + 15 (state pathways) + 5 (type pathways) + 20 (context) = 75  NK cells: 25 (markers) - 10 (shared) - 20 (conflicting pathways) + 20 (context) = 15  Effector Memory CD8+: 45 (markers) - 10 (shared) + 15 (state pathways) + 5 (type pathways) + 20 (context) = 75 |
| --- |

Cytotoxic CD8+ T Cells: 75

NK Cells: 15

Effector Memory CD8+ T Cells: 75

**Report**

**Celltype Prediction**

**Cytotoxic CD8+ T Cells (score: 75)**

Active cytotoxic effector state

**Key Markers**:

- Cell-type-specific: CD3D, CD8A, LCK
- Cell-state-specific: GZMA, GZMK, GZMH, GZMM, PRF1, KLRG1

**Evidence and Reasoning**

The presence of CD3D and LCK definitively establishes T cell lineage identity, while CD8A specifically indicates CD8+ T cell subset. The robust expression of multiple granzyme family members (GZMA, GZMK, GZMH, GZMM) and perforin (PRF1) demonstrates active cytotoxic capacity. This molecular profile is strongly supported by enriched TCR signaling and T cell activation pathways. The co-expression of IL32 and CCL5 suggests these cells are in an effector state, capable of both cytotoxicity and inflammatory cytokine production. The presence of KLRG1 further supports an effector phenotype. This profile is entirely consistent with the biological context of healthy PBMCs, where cytotoxic T cells serve as key immune surveillance cells.

**Validation**: CD3E, CD3G, CD8B

**Effector Memory CD8+ T Cells (score: 75)**

Memory effector state with cytotoxic capacity

**Key Markers**:

- Cell-type-specific: CD3D, CD8A, LCK
- Cell-state-specific: CCL5, KLRG1, IL32

**Evidence and Reasoning**

The core T cell identity markers (CD3D, LCK) combined with CD8A establish the CD8+ T cell lineage. The memory effector state is supported by the expression of CCL5 and KLRG1, which are characteristic of antigen-experienced T cells. The enriched TCR signaling pathways and T cell activation signatures align with memory T cell functionality. The presence of cytotoxic molecules (granzymes and perforin) indicates these cells maintain cytotoxic capacity, typical of effector memory cells. IL32 expression suggests these cells retain inflammatory potential. This phenotype is physiologically relevant in healthy PBMCs, where memory T cells persist following previous immune responses to maintain long-term immunity.

**Validation**: EOMES, PRDM1, TBX21

**NK Cells (score: 15)**

Cytotoxic effector state

**Key Markers**:

- Cell-type-specific: NKG7, KLRG1
- Cell-state-specific: GZMA, GZMK, GZMH, GZMM, PRF1

**Evidence and Reasoning**

While NK cells share many cytotoxic molecules with CD8+ T cells, the presence of NKG7 and KLRG1 provides some evidence for NK cell identity. However, the enriched TCR signaling pathways conflict with NK cell biology, as NK cells lack TCR-mediated activation mechanisms. The cytotoxic program (granzymes and perforin) is characteristic of NK cells but not exclusive to them. The absence of definitive NK markers like NCAM1 and FCGR3A, combined with the strong T cell signature, makes NK cells a less likely candidate despite their natural presence in PBMCs and similar cytotoxic functions.

**Validation**: NCAM1, FCGR3A, KLRD1

**Novel Insights**

The gene expression profile reveals a particularly active cytotoxic program with multiple granzyme family members expressed simultaneously, suggesting a highly potent cytotoxic capacity. The co-expression of IL32 with cytotoxic molecules indicates these cells might possess dual cytotoxic and inflammatory functions. The enrichment of PD-1 blockade pathway suggests these cells may be responsive to immune checkpoint regulation, highlighting their potential role in immune surveillance and tumor immunity. The presence of multiple HLA molecules (HLA-A, B, C) along with B2M indicates active antigen presentation machinery, suggesting these cells might be capable of cross-presentation or enhanced immune synapse formation.

**cluster geneset 5**

**Gene List**

Top genes

| Plain Text LYZ,S100A9,S100A8,TYROBP,FTL,FCN1,CST3,S100A6,FTH1,LGALS2,LGALS1,GSTP1,S100A4,AIF1,TYMP,OAZ1,LST1,GPX1,CTSS,SAT1,S100A11,COTL1,PSAP,FCER1G,CYBA |
| --- |

enrichment pathway

| Plain Text  - WikiPathways_2024_Human: Nonalcoholic Fatty Liver Disease ,TYROBP Causal Network In Microglia ,Microglia Pathogen Phagocytosis Pathway ,Electron Transport Chain OXPHOS System In Mitochondria ,Ebola Virus Infection In Host |
| --- |

**celltype thinking**

| Plain Text Hmm, looking at this gene signature from cluster 5... very interesting pattern emerging here. Let me dive deep into what these genes are telling us about cellular identity.  First striking observation - S100A8/A9 heterodimer, also known as calprotectin. That's a major danger signal and antimicrobial protein, highly expressed in myeloid cells. Along with LYZ (lysozyme), this strongly points to cells with antimicrobial functions.  TYROBP (DAP12) catches my attention - it's a key adaptor molecule in myeloid cells, particularly important for their activation and function. Often works with FCER1G which is also present here... this pathway enrichment for TYROBP causal network makes perfect sense now.  The S100 protein family representation is remarkable - S100A4, S100A6, S100A11 in addition to S100A8/A9. These calcium-binding proteins are characteristic of myeloid cells, especially those involved in inflammation and danger response.  FCN1 is particularly interesting - it's a pattern recognition molecule highly specific for classical monocytes. Combined with LGALS1/2 (galectins), this suggests cells involved in pathogen recognition and immune response initiation.  Looking at the pathway enrichments... microglia pathogen phagocytosis pathway stands out. While these aren't microglia (we're looking at PBMCs), this pathway highlights the phagocytic capacity of these cells. The TYROBP network enrichment further supports myeloid identity.  The presence of FTL/FTH1 (ferritin chains) along with CYBA (part of the phagocyte oxidase complex) suggests cells equipped for managing iron and oxidative stress - typical of professional phagocytes.  CST3 (cystatin C) and CTSS (cathepsin S) point to active lysosomal function and antigen processing capability. Combined with AIF1 (allograft inflammatory factor 1), this suggests professional antigen presenting cells.  Cross-referencing with the candidate cell types... This profile aligns most strongly with:  1. Classical Monocytes (CD14+ monocytes) - the FCN1, LYZ, S100A8/9 signature is quintessential 2. Non-Classical Monocytes - LST1 presence suggests this as a possibility, though the overall signature is more classical 3. Dendritic Cells - while possible given some markers, the strong S100A8/9 expression makes this less likely  For validation, I'd look for: - CD14 and CCR2 as gold standard markers for classical monocytes - CX3CR1 for non-classical monocytes - CD1c/BDCA-1 for dendritic cells  The inflammatory signature suggests these cells are in a slightly activated state, but still within normal range for circulating monocytes in healthy individuals. The metabolic and oxidative stress response genes (GPX1, GSTP1) are consistent with normal monocyte function. |
| --- |

Based on comprehensive analysis of the gene expression profile, pathway enrichment, and biological context, here are the three most likely cell types in order of probability:

1. Classical Monocytes (CD14+ Monocytes)

- Primary evidence: High expression of FCN1, LYZ, S100A8/9
- Supporting evidence: TYROBP signaling pathway, phagocytosis-related genes
- Functional profile: Strong innate immune and antimicrobial signature
- Validation markers: CD14, CCR2

1. Non-Classical Monocytes

- Primary evidence: LST1, TYROBP, FCER1G expression
- Supporting evidence: Inflammatory mediators and stress response genes
- Functional profile: Inflammatory response capacity
- Validation markers: CX3CR1, CD16

1. Myeloid Dendritic Cells

- Primary evidence: Antigen processing machinery (CTSS, CST3)
- Supporting evidence: FCER1G, antigen presentation capability
- Functional profile: Professional antigen presentation
- Validation markers: CD1c, CLEC10A

The strongest evidence points to Classical Monocytes as the most likely identity, given the characteristic expression of monocyte-specific genes and enrichment of relevant functional pathways.

**Score**

| Plain Text Let me carefully evaluate each predicted cell type using the scoring criteria with pathway since we have pathway information...  Starting with Classical Monocytes: Marker profile analysis... Strong positive markers present: LYZ, S100A8/9, FCN1, TYROBP - these are highly specific for classical monocytes, especially FCN1. That's worth significant points in matching markers. The myeloid-specific markers like FCER1G add to the broad category match. No negative markers are present that would exclude classical monocytes. Looking at pathway matches... TYROBP network and phagocytosis pathways perfectly align with monocyte function. The metabolic and inflammatory pathways are consistent with normal monocyte state. Context is perfect - classical monocytes are abundant in healthy PBMCs.  For Non-Classical Monocytes: Marker evaluation... LST1 and some inflammatory markers overlap, but less specific alignment. Many markers shared with classical monocytes - need to deduct points there. Pathways are somewhat relevant but less specific. Context is appropriate though - they're normally present in PBMCs.  For Myeloid DCs: Looking at markers... CTSS and antigen processing machinery present but these are shared with monocytes. Missing some key DC-specific markers, though we can't penalize for missing markers. Pathways partially align but are more monocyte-centric. Context is plausible - DCs circulate in blood.  Calculating final scores...  Classical Monocytes: +40 for specific markers (FCN1, LYZ, S100A8/9) +15 for narrow markers (TYROBP, FCER1G) +15 for matching pathways (TYROBP, phagocytosis) +5 for cell type pathways +20 for perfect biological context Total: 95  Non-Classical Monocytes: +25 for some matching markers +5 for narrow markers -10 for shared markers with classical +10 for partially matching pathways +20 for biological context Total: 50  Myeloid DCs: +20 for some matching markers +5 for narrow markers -10 for shared markers +5 for partially matching pathways +20 for biological context Total: 40 |
| --- |

Classical Monocytes: 95

Non-Classical Monocytes: 50

Myeloid Dendritic Cells: 40

**Report**

**Celltype Prediction**

**Classical Monocytes (score: 95)**

Homeostatic surveillance state with moderate inflammatory activation

**Key Markers**:

- Cell-type-specific: LYZ, FCN1, TYROBP, FCER1G
- Cell-state-specific: S100A8, S100A9, CYBA, GPX1, GSTP1

**Evidence and Reasoning**

The expression profile strongly indicates classical monocytes through multiple lines of evidence. The presence of FCN1 and LYZ represents canonical monocyte markers, while the S100A8/A9 heterodimer complex is characteristically expressed in myeloid cells, particularly monocytes. The enrichment of TYROBP Causal Network and Microglia Pathogen Phagocytosis pathways strongly supports phagocytic cell identity. The oxidative stress response genes (GPX1, GSTP1) and iron metabolism regulators (FTL, FTH1) reflect normal monocyte metabolic functions. The presence of antigen-processing machinery (CTSS, CST3) and pattern recognition molecules (FCN1, LGALS1/2) indicates professional phagocyte identity. This molecular signature, combined with their natural presence in PBMCs, provides compelling evidence for classical monocyte identity.

**Validation**: CD14, CCR2, CSF1R

**Non-Classical Monocytes (score: 50)**

Patrolling state with inflammatory potential

**Key Markers**:

- Cell-type-specific: LST1, TYROBP, FCER1G
- Cell-state-specific: S100A8, S100A9, CYBA

**Evidence and Reasoning**

The presence of LST1, a marker associated with non-classical monocytes, along with TYROBP and FCER1G suggests non-classical monocyte identity. The inflammatory mediators S100A8/A9 and oxidative stress response genes indicate cells capable of mounting inflammatory responses. The enrichment of pathogen response pathways aligns with their surveillance function. However, the strong expression of classical monocyte markers like FCN1 somewhat weakens this identification. The cell type remains plausible in the PBMC context, as non-classical monocytes regularly circulate in blood. The overall molecular profile suggests cells with inflammatory and patrolling capabilities, though with less specific evidence than classical monocytes.

**Validation**: CX3CR1, CD16 (FCGR3A), SLAN

**Myeloid Dendritic Cells (score: 40)**

Surveillance state with antigen-presenting capacity

**Key Markers**:

- Cell-type-specific: FCER1G, TYROBP
- Cell-state-specific: CTSS, CST3, AIF1

**Evidence and Reasoning**

The presence of antigen-processing machinery (CTSS, CST3) and immune signaling molecules (FCER1G, TYROBP) suggests professional antigen-presenting cells. The enrichment of pathogen response pathways aligns with DC function in immune surveillance. However, many markers overlap with monocyte signatures, and the strong expression of S100A8/A9 is less typical for DCs. While DCs are naturally present in PBMCs, the molecular profile shows stronger alignment with monocyte characteristics. The antigen-processing and presentation machinery could reflect the general myeloid cell capacity for immune response rather than DC-specific identity.

**Validation**: CD1c, CLEC10A, ZBTB46

**Novel Insights**

The gene expression profile reveals an interesting pattern of heightened oxidative stress response and iron metabolism regulation (GPX1, GSTP1, FTL, FTH1) alongside classical immune functions. This suggests a previously underappreciated role for metabolic regulation in maintaining monocyte homeostasis in healthy conditions. The enrichment of both TYROBP signaling and mitochondrial pathways points to potential cross-talk between immune signaling and metabolic regulation. The presence of multiple S100 family proteins (S100A4, S100A6, S100A11) beyond the well-known S100A8/A9 heterodimer suggests a broader role for calcium-dependent signaling in monocyte function than typically discussed.

**cluster geneset 6**

**Gene List**

Top genes

| Plain Text HLA-DPA1,HLA-DPB1,HLA-DRA,HLA-DRB1,CD74,HLA-DQA1,CST3,FCER1A,HLA-DRB5,HLA-DMA,HLA-DQB1,HLA-DQA2,LSP1,LYZ,CLEC10A,AP1S2,GRN,GPX1,VIM,GSTP1,CPVL,HLA-DMB,ANXA2,H2AFY,LGALS1 |
| --- |

enrichment pathway

| Plain Text  - WikiPathways_2024_Human: Nonalcoholic Fatty Liver Disease ,Electron Transport Chain OXPHOS System In Mitochondria ,Ebola Virus Infection In Host ,VEGFA VEGFR2 Signaling ,TYROBP Causal Network In Microglia |
| --- |

**celltype thinking**

| Plain Text Hmm, looking at this gene signature from PBMCs... Let me dive deep into the molecular profile here. The strong HLA class II signature immediately catches my attention - HLA-DPA1, HLA-DPB1, HLA-DRA, HLA-DRB1, HLA-DQA1... This is screaming antigen-presenting cell lineage.  FCER1A is particularly interesting - that's a classic dendritic cell marker, especially seen on conventional DCs. And CLEC10A (CD301) - that's another DC-associated C-type lectin receptor. This combination is starting to paint a picture...  The presence of CST3 and LYZ makes me also consider myeloid lineages more broadly. Could these be monocytes? But the strong FCER1A signal keeps pulling me back to DCs...  Let me think about the pathway enrichment... TYROBP (DAP12) signaling in microglia is intriguing - while these aren't microglia (we're looking at PBMCs), this pathway is important in myeloid cells broadly. The metabolic signatures with OXPHOS and NAFLD pathways suggest these cells are metabolically active.  Looking at the candidate cell types... The DC markers align really well, especially for conventional DCs. The HLA class II profile matches perfectly. But I should also seriously consider monocytes given some of these markers.  What about other immune cells? While we see some markers that can be expressed by B cells (HLA class II), the full profile doesn't fit well with B cells - missing key B cell-specific markers. T cells and NK cells are even less likely given this profile.  The presence of VIM (vimentin) and LGALS1 (galectin-1) suggests these cells may have some tissue-trafficking potential, which both DCs and monocytes possess.  Let me think about validation markers we could use... For cDCs, we could look for CD1C or CLEC9A. For monocytes, CD14 or CD16 would be definitive. For pDCs, LILRA4 or IL3RA would be key.  Weighing all the evidence: 1. Conventional DCs seem most likely given FCER1A + CLEC10A + strong HLA-II 2. Classical monocytes are possible but slightly less likely 3. Plasmacytoid DCs seem less likely given the marker profile  Need to consider cell states too... The metabolic activity and trafficking markers suggest these cells may be in an activated or maturation state rather than completely quiescent. |
| --- |

Based on comprehensive analysis of the molecular profile, pathway enrichment, and biological context, the three most likely cell types are:

1. Conventional Dendritic Cells (cDCs)

- Primary evidence: FCER1A, CLEC10A expression
- Supporting evidence: Strong HLA class II signature (HLA-DPA1, HLA-DPB1, HLA-DRA, etc.)
- Validation markers: CD1C, CLEC9A (not in cluster)

1. Classical Monocytes

- Primary evidence: LYZ, CST3 expression
- Supporting evidence: Myeloid-associated pathways
- Validation markers: CD14, CCR2 (not in cluster)

1. Plasmacytoid Dendritic Cells (pDCs)

- Primary evidence: HLA class II machinery
- Supporting evidence: Antigen processing capability
- Validation markers: IL3RA, LILRA4 (not in cluster)

The cells appear to be in an activated/mature state based on their metabolic profile and expression of trafficking-related genes.

**Score**

| Plain Text Alright, let me carefully evaluate each predicted cell type using the scoring criteria with pathways since we have pathway information...  Starting with conventional DCs: Marker profile analysis... HLA class II molecules (HLA-DPA1, DPB1, DRA, etc) - these are strong markers but shared with other APCs FCER1A - this is a really specific DC marker, especially for cDCs CLEC10A - another quite specific DC marker No negative markers present Looking at pathways... TYROBP signaling fits well with DC biology, OXPHOS suggesting active cells Context is perfect - cDCs are definitely expected in healthy PBMCs  For Classical Monocytes: Marker wise...  LYZ and CST3 are present but they're somewhat broad myeloid markers HLA class II - monocytes do express these but not as strongly as DCs Missing some really specific monocyte markers but that's not a negative Pathways partially fit - TYROBP and metabolic pathways make sense Context is appropriate for PBMCs  For pDCs: Looking at markers... HLA class II fits but is shared Missing really specific pDC markers (though we can't penalize for that) FCER1A is actually somewhat contradictory for pDCs Pathways don't align as well with typical pDC biology Context is plausible but other features don't support strongly  Let me calculate specific scores:  cDCs: Markers: 45 (strong specific markers) + 12 (narrow markers) - 10 (shared markers) = 47 Pathways: 15 (state) + 5 (type) = 20 Context: 10 (type) + 10 (state) = 20 Total: 87  Classical Monocytes: Markers: 30 (moderate specific markers) + 8 (narrow markers) - 10 (shared) = 28 Pathways: 10 (state) + 5 (type) - 10 (shared) = 5 Context: 10 (type) + 10 (state) = 20 Total: 53  pDCs: Markers: 20 (weak specific markers) + 5 (narrow markers) - 10 (shared) = 15 Pathways: 5 (type) - 10 (shared) = -5 Context: 10 (type) + 5 (state) = 15 Total: 25 |
| --- |

Conventional Dendritic Cells: 87

Classical Monocytes: 53

Plasmacytoid Dendritic Cells: 25

**Report**

**Celltype Prediction**

**Conventional Dendritic Cells (score: 87)**

Active antigen-presenting state with enhanced metabolic activity

**Key Markers**:

- Cell-type-specific: FCER1A, CLEC10A, HLA-DPA1, HLA-DPB1, HLA-DRA
- Cell-state-specific: VIM, LGALS1, GPX1, GSTP1

**Evidence and Reasoning**

The cell population shows definitive conventional DC characteristics through expression of canonical markers FCER1A and CLEC10A, combined with robust HLA class II machinery (HLA-DPA1, HLA-DPB1, HLA-DRA, HLA-DRB1). The enrichment of TYROBP Causal Network pathway strongly supports myeloid lineage identity, while OXPHOS pathway activation suggests metabolically active cells. The presence of trafficking-associated proteins (VIM, LGALS1) and antioxidant enzymes (GPX1, GSTP1) indicates these cells are in an activated state, consistent with their antigen-presenting function. This molecular profile, found in healthy PBMC context, strongly aligns with conventional DCs' role in immune surveillance and antigen presentation.

**Validation**: CD1C, CLEC9A, IRF4, ZBTB46

**Classical Monocytes (score: 53)**

Activated inflammatory state

**Key Markers**:

- Cell-type-specific: LYZ, CST3, HLA-DRA
- Cell-state-specific: VIM, GSTP1, LGALS1

**Evidence and Reasoning**

The expression of myeloid-associated genes LYZ and CST3, along with HLA class II molecules, suggests a monocytic identity. The TYROBP Causal Network pathway enrichment supports myeloid lineage commitment, while metabolic pathway activation (OXPHOS, NAFLD) indicates high cellular activity. The presence of VIM and LGALS1 suggests these cells may be involved in trafficking or adhesion processes. While these features are consistent with classical monocytes, the expression levels of certain DC-specific markers (FCER1A, CLEC10A) make this a secondary possibility. The profile is compatible with monocytes in healthy PBMCs, though some markers overlap with other myeloid populations.

**Validation**: CD14, CCR2, CD11B, CSF1R

**Plasmacytoid Dendritic Cells (score: 25)**

Partially activated state

**Key Markers**:

- Cell-type-specific: HLA-DRA, HLA-DPA1, HLA-DPB1
- Cell-state-specific: GSTP1, GPX1

**Evidence and Reasoning**

While the strong HLA class II expression pattern aligns with pDC identity, the presence of FCER1A and CLEC10A is less typical for this cell type. The metabolic pathway signatures suggest active cellular processes, but the overall profile lacks key pDC-specific features. The TYROBP pathway enrichment is not characteristically associated with pDCs. Although pDCs are present in healthy PBMCs, the molecular signature more strongly supports other myeloid lineages. The antioxidant enzymes suggest cellular activation, but the overall evidence for pDC identity is relatively weak compared to other possibilities.

**Validation**: IL3RA, LILRA4, TCF4, CLEC4C

**Novel Insights**

The molecular profile reveals an interesting intersection of antigen presentation and cellular trafficking capabilities, suggesting these cells may be actively engaged in immune surveillance. The strong antioxidant signature (GPX1, GSTP1) combined with OXPHOS pathway activation indicates a metabolically active state that may be important for their function. The presence of VEGFA-VEGFR2 signaling pathway enrichment suggests potential involvement in tissue remodeling or vascular interactions, which is an interesting feature for circulating immune cells. This could represent a novel aspect of DC/myeloid cell biology in maintaining vascular homeostasis.

**cluster geneset 7**

**Gene List**

Top genes

| Plain Text PF4,SDPR,GNG11,PPBP,NRGN,SPARC,GPX1,TPM4,RGS18,CALM3,RGS10,TAGLN2,MYL6,OAZ1,GP9,HIST1H2AC,CD9,MYL12A,CCL5,ITM2B,SH3BGRL3,ACTB,H3F3A,PRDX6,AP001189.4 |
| --- |

enrichment pathway

| Plain Text  - WikiPathways_2024_Human: Pathogenic Escherichia Coli Infection ,VEGFA VEGFR2 Signaling ,Ferroptosis ,Striated Muscle Contraction Pathway ,Focal Adhesion |
| --- |

**celltype thinking**

| Plain Text Hmm, looking at this geneset from cluster 7 in healthy PBMC context... these markers are quite interesting and distinctive. Let me think this through carefully...  PF4, PPBP, GP9 immediately jump out - those are classic platelet markers. But wait, this is a PBMC sample... platelets do often contaminate PBMC preps though. The high expression of these markers is quite telling.  Looking deeper at the gene signature... SDPR, GNG11, NRGN, RGS18 - these are also strongly associated with platelets and megakaryocyte lineage. The presence of cytoskeletal/structural proteins like TPM4, TAGLN2, MYL6, ACTB makes sense given platelets' need for shape change and activation.  The pathway enrichment is also quite revealing - VEGF signaling, focal adhesion, muscle contraction pathways all align with platelet function in terms of adhesion, shape change and release of factors like VEGF.  But I should consider other possibilities... Could these be megakaryocytes? Not likely in peripheral blood. CTCs maybe, given CD9? But the other epithelial markers are missing and this is from healthy donor.  What about other blood cells? CCL5 is expressed by T cells and NK cells, but the overall signature doesn't fit their profiles. The absence of key immune markers like CD3 for T cells or NCAM1 for NK cells argues against those.  Looking at candidate cell types... this signature doesn't cleanly match any of the immune cell populations listed. The overwhelming presence of platelet markers suggests these are indeed platelets, despite them being a "contaminant" in PBMC preps.  For validation, I could suggest looking for other platelet-specific markers not in this set like: - CD41 (ITGA2B) - CD61 (ITGB3)  - CD42b (GP1BA) These would definitively confirm platelet identity.  The cell state appears to be relatively resting/unstimulated based on the gene expression pattern - not seeing strong signatures of platelet activation like P-selectin upregulation.  While platelets aren't strictly PBMCs, their presence here is a common technical reality in PBMC isolation. The evidence for platelet identity is quite strong based on both positive markers and pathway analysis. |
| --- |

Based on comprehensive analysis of the markers, pathways, and biological context, here are the three most likely cell type possibilities for this cluster, in order of probability:

1. Platelets

- Key markers: PF4, PPBP, GP9, SDPR, GNG11
- Supporting pathways: VEGF signaling, focal adhesion
- Biological rationale: Common contaminant in PBMC preparations
- State: Relatively unstimulated/resting

1. Megakaryocyte-Derived Bodies

- Key markers: PF4, PPBP, TPM4, MYL6
- Supporting pathways: Muscle contraction, focal adhesion
- Biological rationale: Platelet precursor fragments
- State: Pre-platelet structures

1. Platelet-Like Particles

- Key markers: CD9, SDPR, GPX1
- Supporting pathways: VEGF signaling
- Biological rationale: Membrane-bound platelet-derived vesicles
- State: Circulating microparticles

For validation, I recommend checking expression of additional platelet-specific markers not present in this gene set:

- ITGA2B (CD41)
- ITGB3 (CD61)
- GP1BA (CD42b)

**Score**

| Plain Text Let me carefully evaluate each predicted cell type using the scoring criteria with pathways since we have pathway information...  Starting with Platelets: Marker Profile: - Strong matching markers: PF4, PPBP, GP9, SDPR, GNG11, RGS18 are highly specific platelet markers (+45) - Narrow markers present: GP9 is particularly specific (+15) - No real negative markers present (no deduction) - Some shared markers like CD9, but expected (-10)  Pathway Profile: - VEGF signaling and focal adhesion perfectly match platelet function (+15) - Muscle contraction pathway aligns with platelet contractile needs (+5) - Some pathway overlap with other cell types but expected (-10)  Biological Context: - Common contaminant in PBMC prep (+10) - Resting state makes sense in healthy donor (+10) Total coming to around 80  For Megakaryocyte-Derived Bodies: Marker Profile: - Share many platelet markers but less specific (+30) - Limited narrow markers (+5) - Should see more megakaryocyte-specific factors (-10)  Pathway Profile: - Pathways partially match (+10) - Many shared with platelets (-10)  Biological Context: - Less likely in peripheral blood (-20) Total around 5  For Platelet-Like Particles: Marker Profile: - Share platelet markers but less comprehensive (+25) - Limited specific markers (+5) - Many shared markers (-10)  Pathway Profile: - Pathways partially match (+10) - Significant overlap (-10)  Biological Context: - Plausible in blood (+10) - State reasonable (+10) Total around 40  The scoring really highlights how strongly the evidence supports platelets versus the alternatives... |
| --- |

Platelets: 80

Megakaryocyte-Derived Bodies: 5

Platelet-Like Particles: 40

**Report**

**Celltype Prediction**

**Platelets (score: 80)**

Resting/unstimulated state

**Key Markers**:

- Cell-type-specific: PF4, PPBP, GP9, SDPR, GNG11, RGS18
- Cell-state-specific: TPM4, TAGLN2, MYL6, ACTB, SPARC

**Evidence and Reasoning**

The presence of canonical platelet markers PF4, PPBP, and GP9 provides strong primary evidence for platelet identity. This is further supported by expression of platelet-specific regulatory proteins like SDPR, GNG11, and RGS18. The enrichment of VEGFA-VEGFR2 signaling and focal adhesion pathways aligns perfectly with platelet function. The expression profile of cytoskeletal components (TPM4, TAGLN2, MYL6, ACTB) and the muscle contraction pathway enrichment reflect the platelet's capacity for shape change. While platelets are not strictly PBMCs, their presence as a technical contaminant in PBMC preparations is well-documented. The relatively low expression of activation markers suggests these are predominantly resting platelets, consistent with samples from healthy donors.

**Validation**: ITGA2B (CD41), ITGB3 (CD61), GP1BA (CD42b)

**Platelet-Like Particles (score: 40)**

Circulating microparticle state

**Key Markers**:

- Cell-type-specific: CD9, SDPR, GPX1
- Cell-state-specific: CALM3, PRDX6, ITM2B

**Evidence and Reasoning**

The expression of CD9 and membrane-associated proteins suggests platelet-derived microparticle identity. The presence of calcium signaling regulators (CALM3) and redox-related proteins (GPX1, PRDX6) is consistent with the molecular composition of platelet-derived microparticles. The enrichment of VEGFA-VEGFR2 signaling pathway indicates retention of platelet-derived signaling capabilities. While sharing many features with intact platelets, the distinct expression pattern of membrane and signaling proteins suggests these may represent platelet-derived particles. Their presence in peripheral blood is biologically plausible as they are known to circulate in healthy individuals.

**Validation**: PDCD6IP, TSG101, LAMP2

**Megakaryocyte-Derived Bodies (score: 5)**

Pre-platelet formation state

**Key Markers**:

- Cell-type-specific: PF4, PPBP, TPM4, MYL6
- Cell-state-specific: HIST1H2AC, H3F3A

**Evidence and Reasoning**

While sharing several markers with mature platelets, the presence of histone proteins (HIST1H2AC, H3F3A) could suggest megakaryocyte-derived bodies. The enrichment of muscle contraction and focal adhesion pathways aligns with the cytoskeletal rearrangements during platelet formation. However, the absence of specific megakaryocyte maturation markers and the peripheral blood origin make this identification less likely. The expression profile more likely represents contaminating platelets rather than true megakaryocyte-derived bodies, which are typically confined to the bone marrow during normal hematopoiesis.

**Validation**: VWF, SELP, MPL

**Novel Insights**

The gene expression profile reveals an interesting balance between structural and signaling components in circulating platelets. The co-expression of cytoskeletal regulators (TPM4, TAGLN2, MYL6) with signaling molecules (RGS18, RGS10) suggests a poised state maintaining both structural integrity and rapid response capability. The enrichment of ferroptosis pathway alongside VEGF signaling points to potential novel aspects of platelet redox regulation. This could indicate previously underappreciated roles for platelets in oxidative stress responses during normal circulation, beyond their well-characterized hemostatic functions.
