# Supplementary material for: GPTBioInsightor—leveraging large language models for transparent scRAN-seq cell type annotations
Source: Bioinform Adv. 2026 Jan 22;6(1):vbag025. doi: 10.1093/bioadv/vbag025 (PMC12975716; doi:10.1093/bioadv/vbag025)
Supplement: vbag025_Supplementary_Data [file vbag025_supplementary_data.zip › Supplementary_figures.docx]

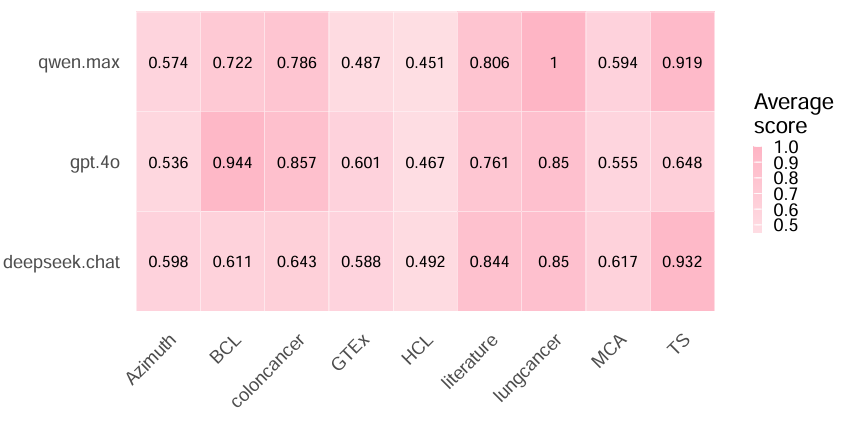


**Figure S1** – Agreement score comparison for qwen-max, gpt-4o, deepseek-chat


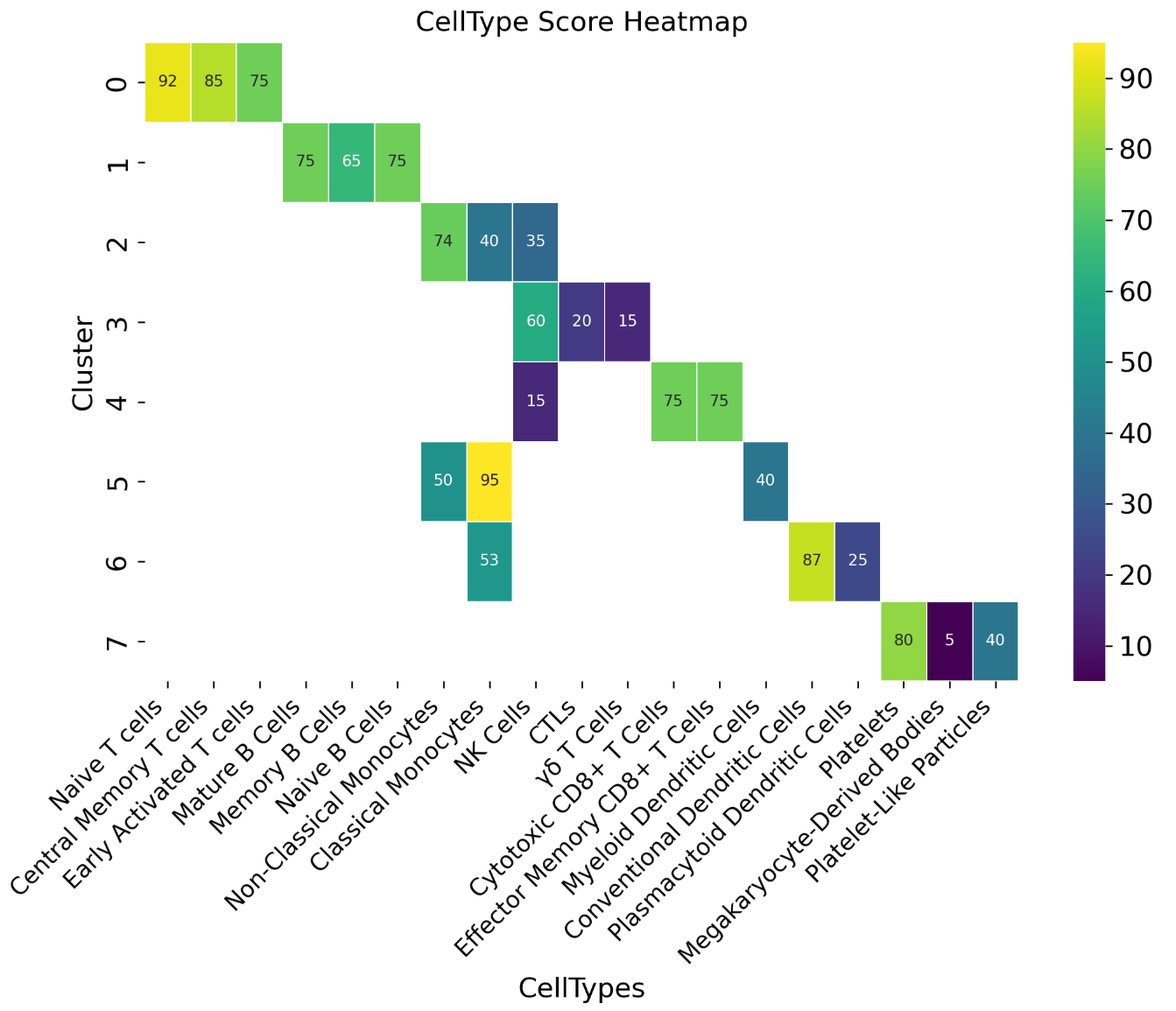


**Figure S2** – Score heatmap of PBMC3K


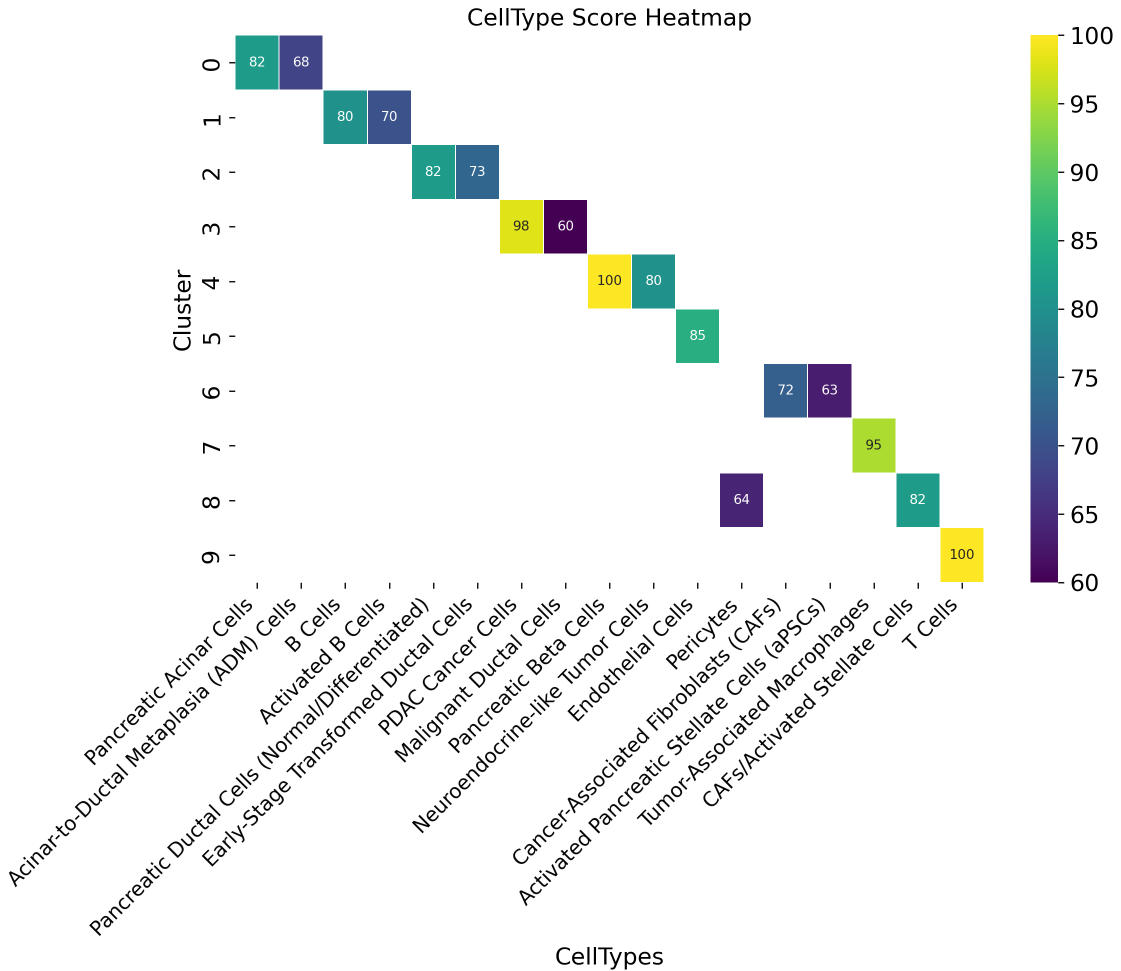


**Figure S3** – Score heatmap of CRA001160
