# Supplementary material for: GPTBioInsightor—leveraging large language models for transparent scRAN-seq cell type annotations
Source: Bioinform Adv. 2026 Jan 22;6(1):vbag025. doi: 10.1093/bioadv/vbag025 (PMC12975716; doi:10.1093/bioadv/vbag025)
Supplement: vbag025_Supplementary_Data [file vbag025_supplementary_data.zip › Supplementary_tables.docx]

**Table S1** – Summary of GPTBioInsightor’s functions

|  | Function | Description |
| --- | --- | --- |
| Cell type and state | list_celltype | get potential cell types according to user provided background information |
|  | get_celltype | get cell types annotation report, including thinking process, reason and celltype evaluation score |
|  | get_subtype | get subtypes of one cell type |
|  | check_celltype | check if cell type prediction of other software is corrected |
|  | get_cellstate | get cell state of cell types based on DEGs |
| Pathway | name_pathway | biological process naming and analysis |
|  | analyse_pathway | Biological Process Analysis |

**Table S2** – GPTBioInsightor’s supported LLM API providers

| API provider | Supported Models |
| --- | --- |
| Anthropic | Claude 3.5 Sonnet, Claude 3.5 Haiku, Claude 3 Opus |
| OpenAI | GPT-4o, GPT-4o-mini, o1 and o1-mini |
| DeepSeek | deepseek-chat |
| OpenRouter | ChatGPT, Claude, deepseek, Qwen,Gemini and more |
| Perplexity | sonar, sonar-pro |
| Groq | Llama, Mixtral, Gemma and more |
| Azure | ChatGPT, deepseek, Qwen,Gemini and more |
| Siliconflow | deepseek, Qwen,Gemini, Gemma and more |
| Aliyun | Qwen,ChatGLM, Llama and more |
| zhipuAI | GLM-4-Plus, GLM-4-Flash, glm-zero-preview and more |
| Meta | Llama |
| ollama | Llama, deepssek, Qwen |
| huggingface | Llama, deepssek, Qwen |

**Table S3** – Runtime and cost of GPTBioInsightor with GPT-4o model using 4 threads

| dataset | cell number(k) | cluster number | cost(S) | time(min) |
| --- | --- | --- | --- | --- |
| coloncancer | 64 | 7 | 0.36 | 2 |
| BCL | 50 | 9 | 0.47 | 3 |
| lungcancer | 208 | 10 | 0.5 | 3 |

**Table S4**– Manual annotation and GPTBioInsightor annotation of PBMC3K dataset

| clusterid | manual annotation | GPTBioInsightor |
| --- | --- | --- |
| 0 | Naive CD4+ T | Naive T cells |
| 1 | B | Mature B Cells |
| 2 | FCGR3A+ Monocytes | Non-Classical Monocytes |
| 3 | NK | NK Cells |
| 4 | CD8 T | Cytotoxic CD8+ T Cells |
| 5 | CD14+ Monocytes | Classical Monocytes |
| 6 | Dendritic | Conventional Dendritic Cells |
| 7 | Platelet | Platelets |

**Table S5** – Manual annotation and GPTBioInsightor annotation of PDAC dataset (CRA001160)

| clusterid | manual annotation | GPTBioInsightor |
| --- | --- | --- |
| 0 | Acinar cell | Pancreatic Acinar Cells |
| 1 | B cell | B Cells |
| 2 | Ductal cell type 1 | Pancreatic Ductal Cells (Normal/Differentiated) |
| 3 | Ductal cell type 2 | PDAC Cancer Cells |
| 4 | Endocrine cell | Pancreatic Beta Cells |
| 5 | Endothelial cell | Endothelial Cells |
| 6 | Fibroblast cell | Cancer-Associated Fibroblasts |
| 7 | Macrophage cell | Tumor-Associated Macrophages |
| 8 | Stellate cell | CAFs/Activated Stellate Cells |
| 9 | Tcell | T Cells |

**Table S6** – Scoring metrics without pathway input

| Marker Profile | Matching cell type or state markers present | max 50 |
| --- | --- | --- |
|  | Narrow markers of cell type or state present | max 20 |
|  | Share common markers with other cell type or state | -15 |
|  | Negative markers present | -30 |
| Biological Context | Plausible cell type in Context | max 15 |
|  | Plausible cell state in Context | max 15 |
|  | implausible cell type or state in Context | -30 |
